# Heterofunctional telechelic polymethylene: from precise synthesis to diverse polyolefin-like polymers

**DOI:** 10.1093/nsr/nwag471

**Published:** 2026-08-04

**Authors:** Xiukuan Yao, Yuxing Zhang, Kangkang Li, Zhongbao Jian

**Affiliations:** State Key Laboratory of Polymer Science and Technology, Laboratory of Polymer Materials Engineering, Changchun Institute of Applied Chemistry, Chinese Academy of Sciences, Changchun 130022, China; School of Applied Chemistry and Engineering, University of Science and Technology of China, Hefei 230026, China; State Key Laboratory of Polymer Science and Technology, Laboratory of Polymer Materials Engineering, Changchun Institute of Applied Chemistry, Chinese Academy of Sciences, Changchun 130022, China; State Key Laboratory of Polymer Science and Technology, Laboratory of Polymer Materials Engineering, Changchun Institute of Applied Chemistry, Chinese Academy of Sciences, Changchun 130022, China; State Key Laboratory of Polymer Science and Technology, Laboratory of Polymer Materials Engineering, Changchun Institute of Applied Chemistry, Chinese Academy of Sciences, Changchun 130022, China; School of Applied Chemistry and Engineering, University of Science and Technology of China, Hefei 230026, China

**Keywords:** precision chemistry, telechelic polyolefin, tailored polymethylene, polyhomologation, block copolymers

## Abstract

Telechelic polyolefins are key building blocks for constructing state-of-the-art polyolefins bearing in-chain functionalities such as ester groups, which have attracted significant attention over recent years. However, the precise synthesis of such α,ω-heterofunctional telechelic polyolefins remains challenging. In this study, polyhomologation is used for the synthesis of α-ester-ω-hydroxy telechelic polymethylenes (bearing hexyl side-chains) through the boron-initiated polyhomologation of ylide monomers. These telechelic polymers are structurally similar to telechelic polyolefins. This method allows for precise control over the end-group fidelity ([OH, COOMe] > 99%) and molecular weight, with particularly narrow molecular weight distributions (*Ð* = 1.05–1.21) being achieved. Consequently, this approach enables the production of diverse topologies including linear, branched (side-chain), and block structures. Notably, the molecular weight of telechelic polymethylenes was precisely tailored as required. The obtained telechelic polymethylenes serve as AB-type macromonomers and undergo direct polycondensation to afford high-molecular-weight (147–300 kDa) polyolefin-like polymers featuring in-chain ester groups, imparting diverse microstructures and topologies including high-density polyethylene (PE), polyolefin elastomer (POE), and random as well as alternating olefin block copolymers (OBC).

## Teaser

This study achieves precise control over molecular weight, distribution and branching of AB-type telechelic macromonomers, enabling to tailor diverse polyolefin-like polymers including HDPE, POE, and random as well as alternating OBCs.

## INTRODUCTION

Telechelic polymers feature reactive functional groups at both chain ends [1]. Through post-polymerization reactions, they are widely used to access polymers with diverse microstructures and topologies, such as block copolymers, star polymers, and segmented networks [2]. Generally, telechelic polyolefins are synthesized via olefin metathesis polymerization [3–6], living insertion polymerization [7–14], long-chain degradation [15–20] and other methods such as polyhomologation [21–23]. By designing telechelic polyolefins with hydroxy or ester end-groups as macromonomers (MMs), polyethylene (PE)-like and olefin random/block copolymer-like polymers containing in-chain ester groups have been synthesized via polycondensation, a strategy widely reported in recent years [5–8,24–31]. Among these, two types of MMs have been studied, A_2_- or B_2_-type telechelic polyolefins, and AB-type heterofunctional telechelic polyolefins.

Compared with A_2_- or B_2_-type telechelic polyolefins [3,26,29–31], heterofunctional telechelic polyolefins as AB-type macromonomers stand out because they avoid difficult stoichiometry and the use of additional comonomers during polycondensation to produce desired high-molecular-weight polyolefin-like polymers [32]. Current strategies for preparing AB-type telechelic polyolefins primarily involve metathesis polymerization and coordination polymerization. As shown in Fig. 1a, telechelic polyolefins can be directly synthesized via chain-transfer metathesis polymerization and subsequent hydrogenation [3–6,24–26]. Such telechelic polyolefins can also be obtained through the coordination chain transfer polymerization (CCTP) of ethylene or propylene (Fig. 1b) [7,8].

**Figure 1.**
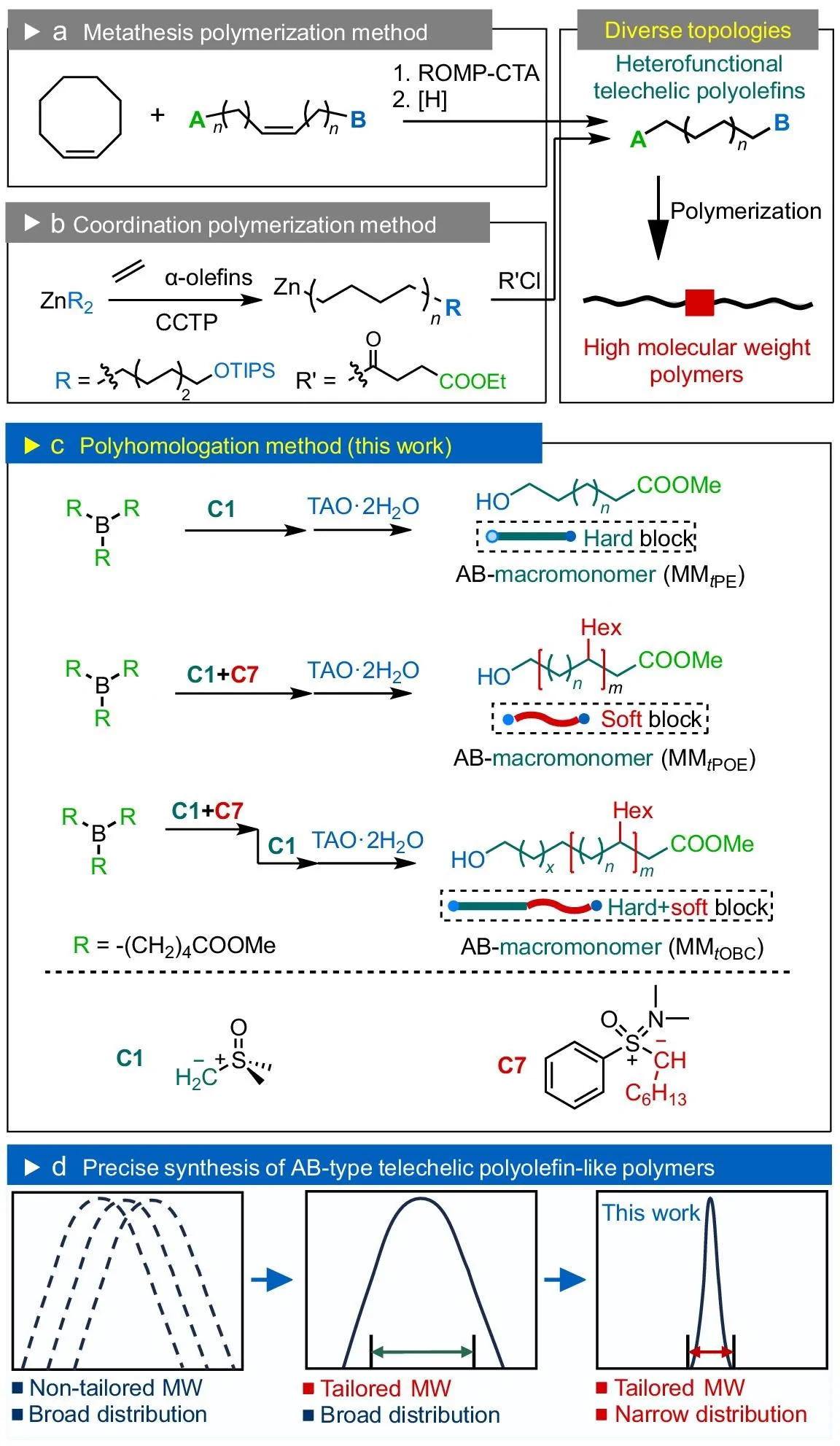
Synthesis of α,ω-heterofunctional telechelic polyolefins (macromonomers) for constructing diverse polyolefins. (a) Metathesis polymerization method. (b) Coordination polymerization method. (c) This work: living polyhomologation method. (d) Three stages of macromonomer synthesis development.

Through polycondensation, telechelic polyolefins as MMs can be polymerized into polyolefins with in-chain ester groups. In this process, the structure of MMs plays a crucial role. There are three key factors: (i) chain-end functionality of MM. MM with functionality < 2 can act as end-capped reagent, affecting the polymer molecular weight. On the other hand, MM with functionality > 2 may introduce branching or even cross-linking [4,15,33]. (ii) Topology of MM. The architecture of the MM determines the topology of the resulting polymers—whether it is branched, block-like, etc. Previous studies have extensively explored this aspect, investigating materials such as polyolefin elastomers (POE) and olefin block copolymer (OBC)-type copolymers [6–8,25,34]. (iii) Molecular weight and polydispersity of MM. This governs the density and uniformity of in-chain ester groups within the polymer [35]. Research in this area has not yet received sufficient attention, particularly regarding tailored molecular weight and controlled polydispersity. These three factors influence the thermomechanical properties of the resulting polymers.

Our group recently achieved tailored molecular weight synthesis in AB-type telechelic PE by employing a ring-strain matched metathesis polymerization approach [19]; but the molecular weight distribution of telechelic PE remained broad (*Đ* > 2.00) (Fig. 1d). Very recently, the well-defined syntheses of A_2_-type (-OH) telechelic PEs via polyhomologation or the Julia–Kocienski reaction were reported [22,36]. However, the precise synthesis of AB-type telechelic polyolefins remains elusive thus far.

Polyhomologation has been established in pioneering reports as an important method for constructing telechelic polyolefin-like polymethylenes. By designing the trialkylborane initiator to introduce specific end-groups, followed by the addition of C1 ylide monomer and subsequent oxidation to yield hydroxyl terminus, telechelic polymethylene can be synthesized [Note: polymethylene is structurally similar to polyethylene (PE), thus the term of PE-like polymer is used in text when necessary] [21,37–40]. Owing to its living polymerization characteristics, the resulting telechelic PE-like polymers typically exhibit narrow molecular weight distributions (*Đ* ≤ 1.2). However, a longstanding challenge is achieving complete and quantitative (> 99%) end-group functionalization to produce ideal α-ester-ω-hydroxy telechelic polymethylenes, which significantly affects the molecular weight of polymers from subsequent AB-type polycondensation [23].

In this study, we employ polyhomologation [23,40–44] for the precise synthesis of α,ω-heterofunctional telechelic polymethylene derivatives with various topologies from ylide monomers **C1** and **C7** (Fig. 1c). This method is anticipated to yield AB-type telechelic polymethylene derivatives with precise control over molecular weight, polydispersity (narrow), and α,ω-heterofunctional end-groups—a level of precision that is beyond those achieved by metathesis or coordination polymerization. These MMs include AB-type telechelic PE-like polymer (**MM*_t_*_PE_**), AB-type telechelic POE (polyolefin elastomer)-like polymer (**MM*_t_*_POE_**), and AB-type telechelic OBC (olefin block copolymer)-like polymer (**MM*_t_*_OBC_**), which can be polymerized into high-molecular-weight and diverse PE, POE, and random as well as alternating OBCs.

## RESULTS

### Precise synthesis of α,ω-heterofunctional telechelic polymethylene derivatives

To access the desired α,ω-heterofunctional telechelic polyolefin-like polymers, a process involving boron-initiated living ylide polyhomologation followed by functional termination was envisaged. Specifically, this protocol begins with an organoboron initiator [BR_3_, R = -(CH_2_)_4_COOMe] to introduce an ester group (-COOMe) at the α-terminus, followed by chain propagation through consecutive ylide monomer insertion into the B–C bond. Termination is subsequently achieved using trimethylamine-*N*-oxide dihydrate (TAO·2H_2_O) as an oxidizing agent, which selectively reacts with the terminal B–C bond to introduce a hydroxyl group (-OH) at the ω-terminus (Table 1).

**Table 1.** Identification of oxidation conditions for the exclusive generation of ω-OH in telechelic polymethylene derivatives.^a^

Entry	Oxidant	*x* (mmol)	*T* (°C)	[OH]%^b^
1	TAO·2H_2_O	6	80	40
2	TAO·2H_2_O	60	80	55
3^d^	TAO·2H_2_O	60	80	55
4^d^	TAO·2H_2_O + NaOH	60	80	80
5^d^	NaBO_3_·4H_2_O	60	80	60
6^e^	NaOH+H_2_O_2_	30	80	80
7^e^	NaOH+H_2_O_2_	120^c^	80	90
8^f^	TAO·2H_2_O	20	110	>99

a Reaction conditions: trialkyl borane (1.0 mmol), N_2_ atmosphere, solvent: toluene + THF, oxidation time: 12 h. ^b^Terminal hydroxyl content, calculated by ^1^H NMR analysis. ^c^H_2_O_2_ was added in several installments. ^d^Oxidation time: 24 h. ^e^Solvent: toluene, oxidation time: 24 h. ^f^This sample is **MM*_t_*_POE-2.5K(17.4%)_** listed in Table 2.

Notably, the non-quantitative formation of ω-OH terminus has been reported in pioneering reports [23], producing AB-type telechelic PE-like polymers with two non-equivalent termini and broad molecular weight distributions (*Đ* reaches 1.76), thereby affording low-molecular-weight polymers (*M*_w_ < 20 kDa) after polycondensation. This indicates the difficulty in achieving quantitative ω-OH terminus. Herein, the highly efficient oxidation conditions (6 equiv. of TAO·2H_2_O at 80°C) that were typically previously applied to B(*t*PE)_3_ were initially tested for the oxidation of B(*t*POE)_3_, producing a relatively low [OH] content of 40% (Table 1, entry 1). Altering the type and amount of oxidizing agent increased the [OH] content by 90% (Table 1, entries 2–7). However, this increase remained insufficient, ultimately producing a polymer with a low molecular weight. After significant optimization, the reaction temperature was identified as a key parameter, with the ω-OH terminus being quantitatively generated (> 99%) at a high temperature of 110°C (exceeding the melting point of TAO·2H_2_O). Consequently, precise α-COOMe-ω-OH telechelic polymethylene derivatives were obtained via this route [ester group at α-terminus (-CH_2_COOCH_3_: CH_2_ (*t*) 2.35 ppm/32.97 ppm; CH_3_ (*s*) 3.71 ppm/51.00 ppm) and hydroxyl group at ω-terminus (-CH_2_OH: CH_2_ (*t*) 3.67 ppm/62.98 ppm)], as confirmed by ^1^H and ^13^C nuclear magnetic resonance (NMR) spectroscopy (Fig. 2a–c and Figs S15–S25). It must be noted that an expected signal at 13.85 ppm (C_2_D_2_Cl_4_, 110°C, 74.00 ppm as the reference) in the ^13^C NMR spectrum will be observed in the partly oxidized telechelic polymethylene derivatives. This signal is attributed to the undesired methyl group at ω-terminus (-CH_3_), originating from incomplete oxidization of the terminal B–C bond. Clearly, there is no signal of ω-CH_3_-terminus in the completely oxidized sample (see compared spectra in Fig. 2b and Fig. S16). This addresses the long-standing challenges associated with the precise synthesis of AB-type telechelic polyolefin-like polymers.

**Figure 2.**
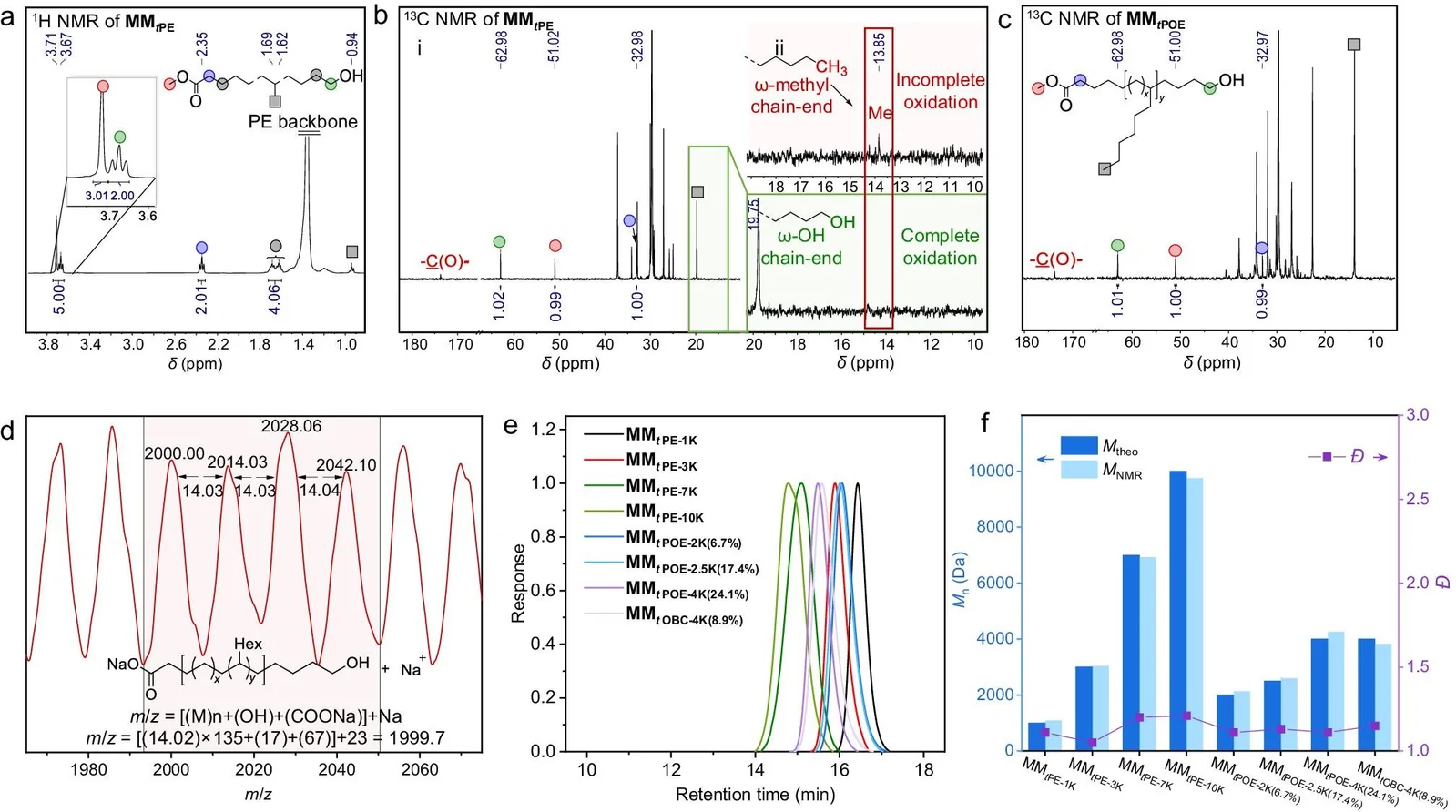
Comprehensive identification of α-ester-ω-hydroxy telechelic polymethylene derivatives. (a) ^1^H NMR and (b) ^13^C NMR spectra of **MM*_t_*_PE-__1K_** listed in Table 2. (c) ^13^C NMR spectrum of **MM*_t_*_POE-2.5K(17.4%)_** listed in Table 2. (d) MALDI-TOF mass spectrum of **MM*_t_*_POE-2.5K(17.4%)_** after hydrolysis. (e) GPC curves of telechelic polymethylene derivatives. (f) Molecular weight distributions (*Đ*) and comparison between theoretical molecular weights (*M*_theo_) and molecular weights (*M*_NMR_) based on NMR data.

To produce distinct macromonomer architectures, dimethylsulfoxonium methylide (**C1**) and the newly designed (dimethylamino)-S-phenylsulfoxonium heptyl ylide (**C7**) were synthesized as comonomers for polyhomologation. Initially, the polyhomologation of **C1** formed an AB-type linear telechelic PE-like polymer as a hard block (**MM*_t_*_PE_**), while the co-polyhomologation of **C1** with **C7** yielded an AB-type telechelic POE-like polymer as a soft block with less crystallinity (**MM*_t_*_POE_**), where telechelic POE-like polymer is defined as ‘soft’ block relative to the telechelic PE-like polymer as ‘hard’ block. Notably, the initial co-polyhomologation of **C1** with **C7** followed by the subsequent block polyhomologation of **C1** generated an AB-type telechelic OBC-like polymer as a hard + soft block (**MM*_t_*_OBC_**). Building on boron-initiated ylide polyhomologation, a series of α-ester-ω-hydroxy telechelic polymethylene derivatives (Table 2) was systematically synthesized to serve as AB-type macromonomers for preparing polyolefin-like polymers with in-chain ester groups. The synthetic procedures employed to produce the monomers, initiators, and (co)polymers are detailed in the Supplementary Information (SI), along with comprehensive data from NMR spectroscopy, mass spectrometry (MS), gel permeation chromatography (GPC), differential scanning calorimetry (DSC), and thermogravimetric analysis (TGA). This polyhomologation approach allowed for precise synthesis of α,ω-heterofunctional telechelic polymethylene derivatives. Specifically, the molecular weights of the AB-type telechelic polymethylene derivatives were precisely tailored as required, depending solely on the [monomer]/[B] feed ratio (i.e., [**C1**]/[B] and [**C1**] + [**C7**]/[B]). In the AB-type telechelic PE-like polymer (**MM*_t_*_PE_**), the molecular weights calculated based on the NMR spectra (*M*_NMR_) were equal to the theoretical molecular weights (*M*_theo_) of 1, 3, 7, and 10 kDa (Fig. 2e–f). The number-average molecular weights calculated based on the GPC data (*M*_n, GPC_) also matched the theoretical molecular weights, and the molecular weight distributions were particularly narrow (*Ð* = 1.05–1.21) (Fig. 2e–f). Importantly, the ^1^H NMR spectra also identified the equivalent α-COOMe (2H, -CH_2_COOMe) and ω-OH (2H, -CH_2_OH) termini (Figs S15–S18), indicating perfect fidelity. This excellent agreement between the theoretical and experimental molecular weights confirms the precision of the developed synthetic approach.

**Table 2.** Precise synthesis of α-ester-ω-hydroxy telechelic polyolefin-like polymers via polyhomologation.^a^

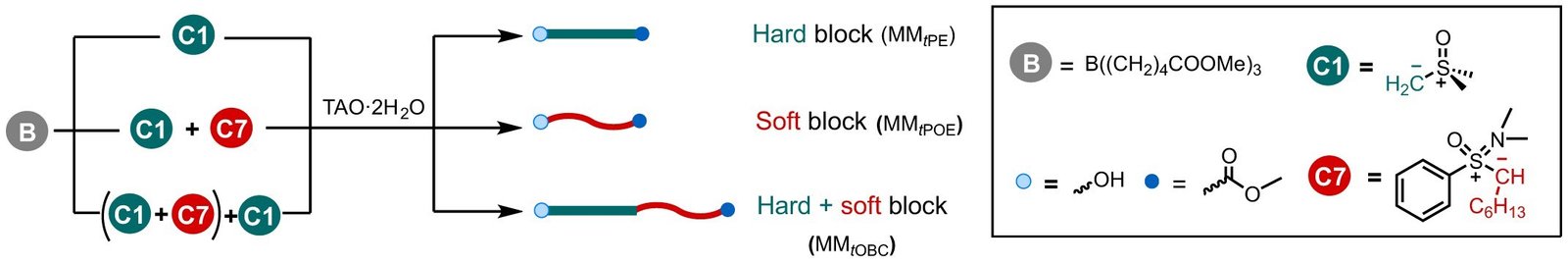								
**MMs**	[C1]/[B]^b^	**C1:C7** (Feeding)	**C1:C7** ^c^ (Macromonomer)	*X*_octene_ (mol%)^c,d^	*M*_theo_ (kDa)^e^	*M*_NMR_ (Da)^c^	*M*_n,GPC_ (Da)^f^	*Ð* ^f^
**MM*_t_*_PE-1K_**	139:0.747	1:0	1:0	-	1	1100	700	1.11
**MM*_t_*_PE-3K_**	150:0.244	1:0	1:0	-	3	3000	2400	1.05
**MM*_t_*_PE-7K_**	156:0.106	1:0	1:0	-	7	6900	7000	1.20
**MM*_t_*_PE-10K_**	157:0.074	1:0	1:0	-	10	9800	9600	1.21
**MM*_t_*_POE-2K(6.7%)_**	120:0.371	30:1	29:1	6.7	2	2100	2500	1.11
**MM*_t_*_POE-2.5K(17.4%)_**	89:0.300	10:1	10.5:1	17.4	2.5	2600	2700	1.13
**MM*_t_*_POE-4K(24.1%)_**	79:0.185	7.5:1	7.3:1	24.1	4	4300	4500	1.11
**MM*_t_*_OBC-4K(8.9%)_** ^g^	115:0.186	20.8:1	21.4:1	8.9	4	3800	3700	1.15

a General polyhomologation conditions: temperature (70°C). Note that two significant figures are used for molecular weights of *M*_NMR_ and *M*_n,GPC_ based on the raw value. ^b^Feed molar ratio of monomer to initiator. ^c^Calculated by ^1^H and ^13^C NMR spectra. ^d^The equivalent octene content was calculated by correlating the branching density of the copolymers with that of standard ethylene-octene copolymer, calculated using Formula III in Supplementary Information. ^e^Theoretical molecular weight, *M*_theo._= 132 + $\frac{{{\mathrm{14\ \times \ [}}{N}_{{\mathrm{C1}}}{\mathrm{]\ + \ 99}}{\mathrm{.2\ \times \ [}}{N}_{{\mathrm{C7}}}{\mathrm{]}}}}{{{\mathrm{3\ \times \ [}}{N}_{\mathrm{B}}{\mathrm{]}}}}$. ^f^Determined by GPC in 1,2,4-trichlorobenzene at 150°C, calibrated by standard parameters of *k* = 40.6 and *α* = 0.725. ^g^The soft block of **MM*_t_*_OBC-4K(8.9%)_** is the same as **MM*_t_*_POE-2.5K(17.4%)._**

Polyhomologation enabled structural diversification through the co-polyhomologation of **C1** and **C7**. Three distinct telechelic polymethylene derivative topologies were obtained, namely hard block **MM*_t_*_PE_** (**C1** homopolymer), soft block **MM*_t_*_POE_** [**C1**/**C7** copolymer: the structure is α-COOMe-ω-OH telechelic polymethylene bearing hexyl side-chain, which is akin to telechelic ethylene/1-octene copolymer (POE), namely telechelic polyethylene bearing hexyl branching], and hard + soft block **MM*_t_*_OBC_** combining both hard and soft segments (Table 2). Steric hindrance of ylide monomer plays a critical role in polyhomologation [42]. The **C7** ylide exhibited substantially lower reactivity than **C1** and could not undergo homo-polyhomologation by itself. Consequently, initial direct co-polyhomologation attempts resulted in almost linear PE-like polymer structures (**MM*_t_*_PE_**) with trace of **C7** incorporation, highlighting the necessity for a controlled monomer addition procedure. To achieve a dispersed distribution of hexyl side-chain (the so-called hexyl branching), the alternating feeding of **C1** and **C7** was performed at set ratios to ensure complete consumption of **C7**. The **C7** monomer is bright yellow and becomes colorless upon complete consumption, allowing visual monitoring of the reaction progress. During a given feeding sequence, addition of **C7** alone did not result in polymerization or molecular weight increase; only upon subsequent addition of **C1** did the molecular weight continue to grow (Fig. S10). After polymerization, termination with TAO·2H_2_O yielded the desired **MM*_t_*_POE_** with dispersed hexyl side-chain (branching) (confirmed by ^13^C NMR, Fig. S22). Further extension of the co-polyhomologation reaction by introducing an additional **C1** monomer prior to the termination of **MM*_t_*_POE_** produced **MM*_t_*_OBC_**, consisting of a hard block and a soft block, indicating a living feature of polyhomologation (*Đ*: 1.13 vs 1.15). Notably, although the block-type **MM*_t_*_OBC__-4K(8.9%)_** contains a higher **C7** content (i.e., more branching), it exhibits a higher melting point than **MM*_t_*_POE__-2K(6.7%)_** (*T*_m_ = 111°C vs. 94°C) and a higher enthalpy change, indicating higher crystallinity (Δ*H* = −75 J/g vs. −9.5 J/g, Figs S36 and S37). This demonstrates the importance of controlling the polymerization sequence and thus the distribution of branching along the polymer chain via monomer feeding, which significantly influences the thermal properties of the products.As shown in Table 2 and Fig. 2, the molecular weights derived from the NMR and GPC in the **MM*_t_*_POE_** and **MM*_t_*_OBC_** data matched the theoretical molecular weights, and the molecular weight distributions were relatively narrow (*Ð* = 1.11–1.15). Once again, the equivalent α-COOMe (2H, -CH_2_COOMe) and ω-OH (2H, -CH_2_OH) termini (Figs S15–S25) were detected by NMR spectroscopy. Besides, matrix-assisted laser desorption/ionization time-of-flight (MALDI-TOF) mass spectrometry further confirmed the >99% end-capped structures. The spectrum of **MM*_t_*_POE-2.5K(17.4%)_** exhibited a single population of peaks with a uniform 14 g/mol mass difference, consistent with methylene unit spacing. The *m*/*z* values adhered to the formula *m*/*z* = 14.02*n* + 17 + 67 + 23 (where 17 accounts for -OH termini, 67 accounts for -COONa formed by hydrolysis of -COOMe and following MALDI process, and 23 for Na⁺), thereby verifying an α-COOMe-ω-OH-terminated structure (Fig. 2d). These results confirm the precise synthesis of not only the typical telechelic PE-like polymer, but also the more challenging telechelic POE-like and OBC-like polymers, identifying the polyhomologation as a powerful and versatile tool for the on-demand synthesis of telechelic polymethylene derivatives with both tailored topology, molecular weights and narrow polydispersities.

### Synthesis of diverse polyolefin-like polymers with in-chain esters

Based on α-COOMe-ω-OH telechelic polymethylene derivatives as AB-type macromonomers bearing inherently equivalently reactive hydroxyl and ester termini, four types of diverse polyolefin-like polymers with in-chain ester group (*d*PO) were synthesized via Ti(O*^n^*Bu)_4_-catalyzed polycondensation. These included in-chain ester HDPE ***d*PE-***n***%** (where *n*% denotes the molar percentage of ester groups in the polymers, calculated using Equations IV–VI in SI), in-chain ester POEs ***d*POE-***n***%**, in-chain ester alternating OBC ***d*(*a*-OBC-***n***%)** and random OBC ***d*(*r*-OBC-***n***%)**, as detailed in Table 3. The high molecular weights (*M*_w_ = 147–300 kDa) achieved for these polymers were attributed to >99% AB-type α-OH-ω-COOMe termini, giving values that were one order of magnitude higher than those achieved in previous reports [23]. Notably, the high molecular weight nature of the polymer plays a critical role in imparting tensile strength to the material.

**Table 3.** Synthesis of diverse polyolefin-like polymers with in-chain ester group (*d*PO) from telechelic polymethylene derivatives (MM).^a^

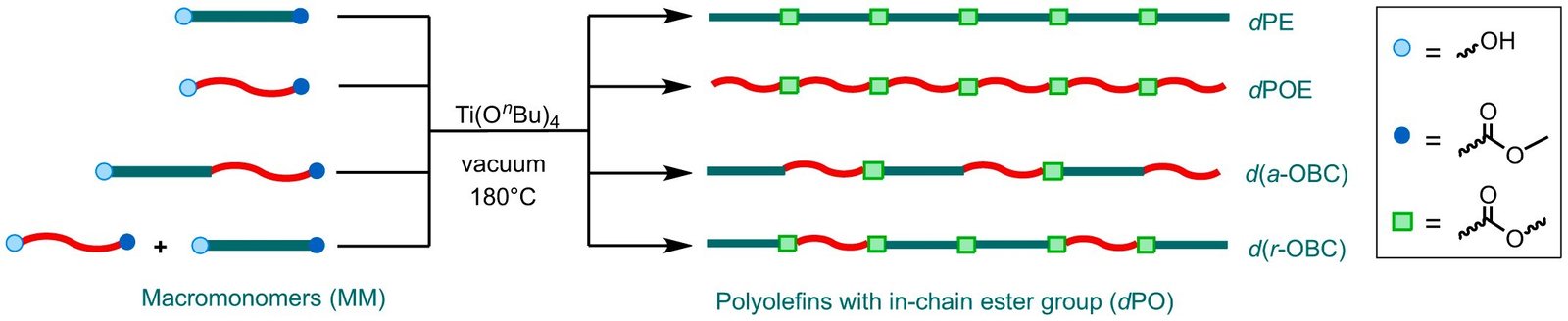								
*d*PO	Telechelic polymethylene derivatives	*X*_octene_ (mol%)^b^	*X*_-COO-_ (mol%)^b^	*M*_n_,_GPC_ (kDa)^c^	*M*_w,GPC_ (kDa)^c^	*Ð* ^c^	*T*_m_ (°C)^d^	*T*_g_ (°C)^d^
***d*PE-0.4%**	**MM*_t_*_PE-7K_**	-	0.4	151	300	1.99	134	n.d.
***d*POE-1.6%**	**MM*_t_*_POE-2K(6.7%)_**	6.7	1.6	75	235	3.13	78	-44
***d*POE-1.7%**	**MM*_t_*_POE-2.5K(17.4%)_**	17.4	1.7	112	232	2.08	35	-59
***d*(*a-*OBC-0.9%)**	**MM*_t_*_OBC-4K(8.9%)_**	8.9	0.9	70	167	2.38	92	-46
***d*(*r-*OBC-0.6%)**	**MM*_t_***_**POE-4K(24.1%)**:_ **MM*_t_*_PE-10K_** = 1:1^e^	12.0	0.6	68	147	2.17	121	-37

a Conditions: The telechelic macromonomer in a flask, a toluene solution of Ti(O*^n^*Bu)_4_ (0.05 mol%) was added, then the temperature was raised to 180°C. Typically, the polymerization was conducted at 180°C under vacuum for 18 h. ^b^Calculated by ^1^H NMR analysis. ^c^Determined by GPC in 1,2,4-trichlorobenzene at 150°C, calibrated by standard parameters of *k* = 40.6 and *α* = 0.725. ^d^Determined by DSC (second heating cycle). ^e^Feeding in a mass ratio.

It was also found that variations in the polymer microstructures imparted diverse macroscopic properties. Specifically, the ***d*PE** series, derived from the homopolymerization of the hard block **MM*_t_*_PE_**, exhibited thermal properties similar to those of HDPE, with a high melting point of 134°C (Table 3, ***d*PE-0.4%**, *T*_m_ = 134°C). ***d*POE** materials prepared from the soft **MM*_t_*_POE_** blocks showed significantly reduced melting points [Table 3, ***d*POE-1.6%**, *T*_m_ = 78°C (*X*_octene_ = 6.7 mol%)], while a higher hexyl side-chain (branching) content further reduced the melting temperature [Table 3, ***d*POE-1.7%**, *T*_m_ = 35°C (*X*_octene_ = 17.4 mol%)] and glass transition temperature (*T*_g_: −59°C vs. −44°C). Specifically, the OBC-based materials exhibited distinctive behaviors. Owing to the concentrated distribution of hexyl side-chains (branchings) along the polymer chain, even with high **C7** incorporation (similar to a high octene content in ethylene/octene-type OBCs), the continuous methylene sequences (-CH_2_-) remained sufficiently long for crystallization. As a result, ***d*(*a*-OBC-0.9%)** (*X*_octene_ = 8.9 mol%) exhibited a higher melting point (*T*_m_ = 93°C) than its random counterpart ***d*POE-1.6%** (*X*_octene_ = 6.7 mol%, *T*_m_ = 78°C), despite having a higher overall hexyl side-chain (branching) content. This observation is consistent with the wide-angle X-ray diffraction (WAXD) results, in which typical signals corresponding to -CH_2_CH_2_- crystallization were observed (Fig. 3a).

**Figure 3.**
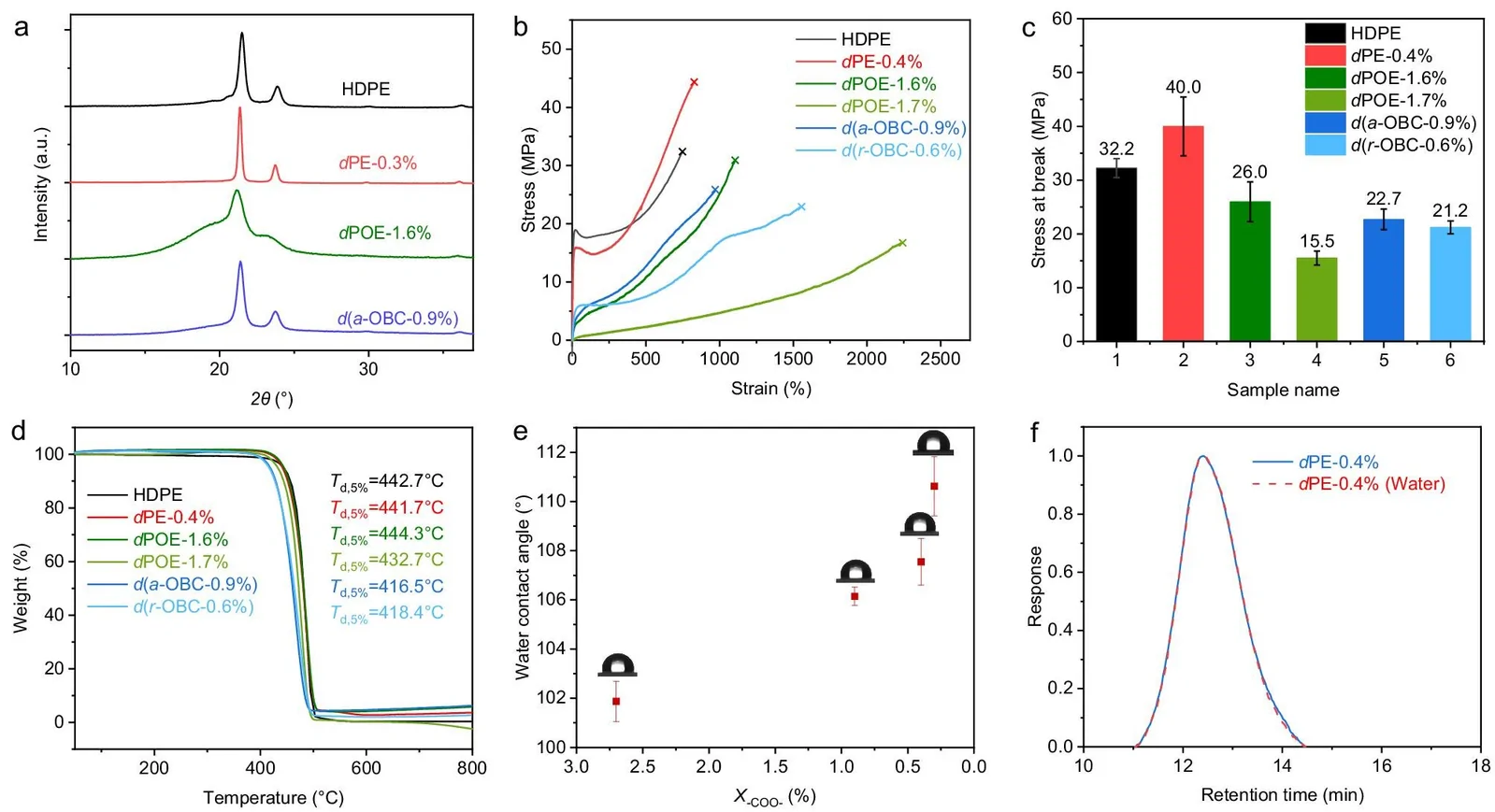
Comprehensive properties of polyolefin-like polymers with in-chain esters. (a) Stacked wide-angle X-ray diffraction (WAXD) profiles. (b) Stress-strain curves. (c) Tensile strengths [data are mean ± s.d. (*n*  =  5 replicates): from left to right: HDPE (32.2 ± 1.8 MPa), ***d*PE-0.4%** (40.0 ± 5.5 MPa), ***d*POE-1.6%** (26.0 ± 3.7 MPa), ***d*POE-1.7%** (15.5 ± 1.3 MPa), ***d*(*a*-OBC-0.9%)** (22.7 ± 1.9 MPa), ***d*(*r*-OBC-0.6%)** (21.2 ± 1.2 MPa). (d) Thermogravimetric analysis (TGA) curves. (e) Water contact angle (WCA) photos [data are mean ± s.d. (*n*  =  3 replicates): from right to left: ***d*PE-0.3%** (110.6 ± 1.2°), ***d*PE-0.4%** (107.6 ± 1.0°), ***d*PE-0.9%** (106.2 ± 0.4°), ***d*PE-2.7%** (101.9 ± 0.8°)]. (f) Gel permeation chromatography (GPC) curves of ***d*PE-0.4%** before and after water treatment (immersed in boiling water for 12 h).

Notably, two types of OBCs with in-chain ester group were constructed, namely ***d*(*a*-OBC)**, which was prepared from the homo-polycondensation of **MM*_t_*_OBC-4K(8.9%)_**, and ***d*(*r*-OBC)**, which was obtained from the random co-polycondensation of **MM*_t_*_POE-4 K_** and **MM*_t_*_PE-10 K_** in a 1:1 mass ratio. The former featured a well-defined alternating hard-*alt*-soft block sequence, a direct consequence of the head-to-tail linkage of the AB-type telechelic macromonomers, where the lengths of the hard and soft segments were precisely controlled by the macromonomer architecture (this soft segment was identical to that in ***d*POE-1.7%**). In contrast, the latter emerged from the random copolymerization of soft and hard telechelic macromonomers, resulting in longer average hard segments. Therefore, although ***d*(*r*-OBC-0.6%)** possessed a higher hexyl side-chain (branching) content (*X*_octene_ = 12.0 mol%), it exhibited a notably higher melting point (*T*_m_ = 121°C) [6,8,25,45]. Combining high *T*_m_ and low *T*_g_, these two OBCs containing in-chain ester groups exhibit thermoplastic elastomeric behavior, which is reminiscent of conventional OBCs synthesized through coordination polymerization [46,47].

Variations in the microstructure were also found to significantly influence the mechanical properties of the materials. Specifically, the linear polymer ***d*PE-0.4%** (*M*_w_ = 300 kDa, *Đ* = 1.99), which possesses a low ester group content (Fig. 3b, red line), exhibited high strength and toughness similar to those of HDPE (*M*_w_ = 263 kDa, *Đ* = 2.07, Fig. S11), albeit with lower crystallinity (Δ*H* = −118 vs. −170 J/g, Figs S38 and S44), which accounts for its reduced modulus. Nevertheless, ***d*PE-0.4%** possessed a tensile strength (stress at break) of 40.0 ± 5.5 MPa (Fig. 3c, red column vs. black column) and an elongation at break of 820% (Fig. 3b, red line vs. black line). The superior stress at break of ***d*PE-0.4%** is likely attributable to enhanced interchain interactions from the in-chain ester groups, which suppress chain slippage during large deformation. It is noted that the molecular weight of polymers with in-chain ester groups plays a decisive role in their mechanical properties. The previously reported ester-containing polymers with *M*_w_ < 20 kDa exhibited poor mechanical performance with tensile strength <10.1 MPa and an extremely low elongation at break <2% [23]. The introduction of side-chain (branching) structures led to a reduction in tensile strength to 26.0 ± 3.7 MPa for ***d*POE-1.6%** and 15.5 ± 1.3 MPa for ***d*POE-1.7%**, although ductility of these materials increased significantly, demonstrating elongations at break of 1100% and 2240%, respectively. This significant difference between these two ***d*POEs** featuring similar ester group densities highlights the critical role of side-chain (branching) in polymer mechanical properties. In contrast, block copolymers combined the features of HDPE and POE to achieve a balance between strength and toughness. Consequently, the alternating block copolymer ***d*(*a*-OBC-0.9%)** (Fig. 3b–c, blue line) exhibited a tensile strength of 22.7 ± 1.9 MPa, which is higher than that of its soft-segment analog ***d*POE-1.7%** (Fig. 3b–c, light green line, 15.5 ± 1.3 MPa). Moreover, owing to the dispersed distribution of hard segments in the alternating structure, ***d*(*a*-OBC-0.9%)** did not exhibit a distinct yield point (Fig. 3b, blue line). On the other hand, the random block copolymer ***d*(*r*-OBC-0.6%)** displayed an obvious yield point with a yield strength of 6.0 MPa despite the slightly decreased tensile strength of 21.2 ± 1.2 MPa (Fig. 3b, light blue line). In a preliminary result, both OBCs with in-chain esters exhibited similar elastic recovery ratio of 36% and 38% after ten repetitive stress–strain cycles at 300% strain (Figs S51–S52).

The effect of in-chain ester groups on polyolefin-like materials has attracted considerable attention within the sustainable polymer research community. Notably, the introduction of ester groups did not compromise the thermal stabilities of the materials in the current systems. Specifically, all synthesized (co)polymers exhibited high thermal decomposition temperatures > 410°C (Fig. 3d). Furthermore, the presence of ester groups in the backbone slightly enhanced the surface hydrophilicity (Fig. 3e). Upon increasing the ester content (Table 2, ***d*PE-0.4%**; Table S1, ***d*PE-0.3%, *d*PE-0.9%, *d*PE-2.7%**), the water contact angle (WCA) progressively decreased from 110.6 ± 1.2° to 107.6 ± 1.0°, 106.2 ± 0.4°, and 101.9 ± 0.8°, respectively. Interestingly, compared to ethylene-methyl acrylate copolymers (namely on-chain ester PE) with a similar ester-group content [48–50], these ***d*PEs** (namely in-chain-ester PE) exhibited higher WCA values. This is likely because the polar ester groups were buried within PE backbone, preventing their presentation at the surface of PE material and thus minimizing the influence of these higher surface-energy groups. Despite its enhanced hydrophilicity, ***d*PE-0.4%** retained a consistent molecular weight without degradation after treatment in boiling water for 12 h under an air atmosphere (Fig. 3f and Fig. S56). Furthermore, under basic catalysis, the in-chain ester bonds of these polymers can undergo solvolysis, leading to chain cleavage and degradation into α-ester-ω-hydroxy terminated MMs. These MMs can then be re-converted back to the parent polymers through polycondensation, thereby enabling a closed-loop recycling pathway (see Section 4.12 in SI).

## CONCLUSION

In summary, polyhomologation was demonstrated for the precise synthesis of α,ω-heterofunctional telechelic polymethylene derivatives that act as AB-type macromonomers to produce diverse polyolefin-like polymers. This method differs significantly from the typical metathesis polymerization and coordination polymerization methods reported previously, contributing a precise method for synthesizing the α-COOMe-ω-OH telechelic macromonomers. Diverse telechelic macromonomers including telechelic HDPE-like, POE-like, and OBC-like macromonomers were thus accessed, exhibiting tailored molecular weights, narrow molecular weight distributions, and perfect end-group fidelity, thereby overcoming the long-standing issue of uncontrolled telechelic polyolefin synthesis. These telechelic polymethylene derivatives possess well-defined molecular weights similar to small-molecule monomers, while also offering greater molecular weight diversity through careful control of the ratio between the ylide monomer and the boron initiator. In addition, their narrow molecular weight distributions enabled the in-chain ester group to be evenly distributed along the backbone of the polymer, while their equivalent AB-type termini ensured the formation of high-molecular-weight polymers.

Consequently, high-molecular-weight poly-olefin-like polymers with in-chain ester groups, including HDPE-, POE-, and OBC-like materials, were quantitatively prepared from precise macromonomers without the requirement for either precise stoichiometry or additional monomers, in which the resulting polyolefin-like polymers exhibit diverse topologies, including linear, side-chain (branched), and block architectures.

Although the relatively low preparation efficiency of this strategy limits its suitability for large-scale production of telechelic polyolefin-like polymers, it remains highly significant for obviating traditional trial-and-error approaches. It provides precise access to α,ω-heterofunctional telechelic polymethylene derivatives with near-ideal molecular weights, which can be converted into diverse polyolefin-like polymers featuring highly controlled in-chain ester group distributions. These polymers could serve as well-defined templates for potentially probing structure–property relationships. Theoretically, by mixing telechelic polymethylene derivatives of different molecular weights, each with a narrow molecular weight distribution, one can obtain a mixture exhibiting broader overall molecular weight distributions. Molecular weight distributions strongly affect the physical properties of resulting polymers. This would allow for the tuning of the molecular weight distribution from narrow to broad nature, providing a potential pathway to access polyolefin-like materials with tailored polydispersity for user-specified properties [51].

## MATERIALS AND METHODS

All materials and methods can be found in the online Supplementary Data.

## Supplementary Material

nwag471_Supplemental_File

## Data Availability

All data needed to evaluate the conclusions in the paper are present in the paper and/or the Supplementary Data. Additional data related to this paper may be requested from the authors.
