# Supplementary material for: Heterofunctional telechelic polymethylene: from precise synthesis to diverse polyolefin-like polymers
Source: Natl Sci Rev. 2026 Aug 4;13(19):nwag471. doi: 10.1093/nsr/nwag471 (PMC13715437; doi:10.1093/nsr/nwag471)
Supplement: Supplementary file 1 [file nwag471_supplemental_file.pdf]

Supplementary Information

Heterofunctional Telechelic Polymethylene: From Precise Synthesis to Diverse Polyolefin-like Polymers

Xiukuan Yao,[a,b,#] Yuxing Zhang,[a,#] Kangkang Li,[a] and Zhongbao Jian[a,b,*]

[a] State Key Laboratory of Polymer Science and Technology, Laboratory of Polymer Materials Engineering, Changchun Institute of Applied Chemistry, Chinese Academy of Sciences, Changchun 130022, China

[b] School of Applied Chemistry and Engineering, University of Science and Technology of China, Hefei 230026, China

*E-mail: zbjian@ciac.ac.cn

Table of Contents

1. Formulas **2**

2. Supplementary Methods **3**

3. Supplementary Tables **6**

4. Supplementary Figures **7**

5. Supplementary References **37**

1. Formulas

1.1 Calculation of M_{NMR} of MMs (AB-type telechelic polyolefins) is performed based on ^{1}H NMR spectra using **Formula I**.

$$\textbf{Formula I:}\ M_{\mathrm{NMR}} = 104.1 + \frac{I_{tot}-7}{I_x} \times 14$$

$$I_{tot} = Integral\ of\ all\ revelent\ signals\ of\ MMs\ in\ {}^1H\ NMR$$
$$I_x = Integral\ of\ signals\ at\ 2.35\ ppm\ in\ {}^1H\ NMR$$

Taking Figure S13 as example, $M_{\mathrm{NMR}} = 104.1 + \frac{I_{tot}-7}{I_x} \times 14 = 104.1 + \frac{148.46-7}{2} \times 14 = 1094\ Da$

1.2 Calculation of the ratio of **C1**:**C7** of MMs (AB-type telechelic polyolefins) is performed based on ^{1}H NMR spectra and ^{13}C NMR spectra using **Formula II**.

$$\textbf{Formula II:}\ C1:C7 = [\frac{I_{tot}}{2} - \frac{7\times I_a}{3\times(I_b+I_c)}]: \frac{I_a}{3\times(I_b+I_c)}$$

$$I_{tot} = Integral\ of\ all\ revelent\ signals\ in\ {}^1H\ NMR$$
$$I_a = integral\ of\ signal\ at\ 0.97\ ppm\ in\ {}^1H\ NMR$$
$$I_b = Integral\ of\ signal\ at\ 19.88\ ppm\ in\ {}^{13}C\ NMR$$
$$I_c = Integral\ of\ signal\ at\ 13.99\ ppm\ in\ {}^{13}C\ NMR$$

Taking Figure S17-18 as example, $C1:C7 = [\frac{I_{tot}}{I_x} - \frac{7\times2\times I_a\times I_c}{3\times(I_b+I_c)\times I_x}]: \frac{2\times I_a\times I_c}{3\times(I_b+I_c)\times I_x} = [\frac{297.44}{2.05} - \frac{7\times2\times17.62\times1}{3\times(0.41+1)\times2.05}]:$

$$\frac{2\times17.62\times1}{3\times(0.41+1)\times2.05} = 116.64 : 4.06 = 29 : 1$$

1.3 Calculation of octene molar content (X_{octene}) is performed based on ratio of C1:C7 of MMs using **Formula III**.

$$\textbf{Formula III:}\ X_{\mathrm{octene}} = \frac{2\times1}{y+1} \times 100\%$$

$$y = C1:C7$$

Taking **MM*t*POE-2K(6.7%)** as example, $y = C1:C7 = 29:1,\ X_{\mathrm{octene}} = \frac{2\times1}{29+1} \times 100\% = 6.7\ \%$

1.4 Calculation of Ester group content (X_{COO}) is performed based on M_{NMR} of *d*PE using **Formula IV**.

$$\textbf{Formula IV:}\ X_{\mathrm{COO}} = \frac{1}{\frac{M_{\mathrm{NMR}}-32-44}{28}+1} \times 100\% = \frac{28}{M_{\mathrm{NMR}}-48} \times 100\%$$

$$M_{\mathrm{NMR}} = The\ M_{\mathrm{NMR}}\ of\ the\ macromonomer\ that\ used\ for\ synthesizing\ dPE$$
32 = MeOH, 44 = ester group

Taking ***d*PE-0.4%** as example, $X_{\mathrm{COO}} = \frac{28}{M_{\mathrm{NMR}}-48} \times 100\% = \frac{28}{6922-48} \times 100\% = 0.4\%$

Taking ***d*PE-0.3%** as example, $X_{\mathrm{COO}} = \frac{28}{M_{\mathrm{NMR}}-48} \times 100\% = \frac{28}{9758-48} \times 100\% = 0.3\%$

1.5 Calculation of Ester group content (X_{COO}) is performed based on M_{NMR} of *d*POE and *d*(*a*-OBC) using **Formula V**.

$$\textbf{Formula V:}\ X_{\mathrm{COO}} = \frac{1}{\frac{(y+1)\times\ (M_{NMR}-32-44)}{28\times(y+7)}+1} \times 100\%$$

$$y = \text{C1:C7}$$

Take ***d*****POE-1.6%** as example, $X_{\text{COO}} = \frac{1}{\frac{(y+1)\times(M_{NMR}-32-44)}{28\times(y+7)}+1}\times 100\% = \frac{1}{\frac{(29+1)\times(2137-76)}{28\times(29+7)}+1}\times 100\% = 1.6\%$

1.6 Calculation of Ester group content (X_{COO}) is performed based on M_{NMR} of *d*(*r*-OBC) using **Formula VI**.

$$\text{Formula V: } X_{\text{COO}} = \frac{\frac{1}{MM_{tPOE}}+\frac{1}{MM_{tPE}}}{\frac{1}{MM_{tPOE}}\times\left[\frac{(y+1)\times(MM_{tPOE}-32-44)}{28\times(y+7)}+1\right]+\frac{1}{MM_{tPE}}\times(\frac{MM_{tPE}-32-44}{28}+1)}\times 100\%$$

$$y = \text{C1:C7 of } \boldsymbol{MM_{tPOE}}$$

For *d*(*r*-OBC-0.6%), $X_{\text{COO}} = \frac{\frac{1}{MM_{tPOE}}+\frac{1}{MM_{tPE}}}{\frac{1}{MM_{tPOE}}\times\left[\frac{(y+1)\times(MM_{tPOE}-32-44)}{28\times(y+7)}+1\right]+\frac{1}{MM_{tPE}}\times(\frac{MM_{tPE}-32-44}{28}+1)}\times 100\% =$

$$\frac{\frac{1}{4260}+\frac{1}{9758}}{\frac{1}{4260}\times(\frac{(7.3+1)\times(4260-32-44)}{28\times(7.3+7)}+1)+\frac{1}{9758}\times(\frac{9758-32-44}{28}+1)} = \mathbf{0.6\%}$$

2. Supplementary Methods

2.1 General experimental details: All syntheses involving air- and moisture-sensitive compounds were carried out using standard Schlenk-type glassware (or in a glove box) under an atmosphere of nitrogen.

2.2 Materials: Trimethylsulfoxonium iodide, sodium hydride, benzyltri(n-butyl)ammonium chloride, trimethoxonium tetrafluoroborate, tetrabutyl titanate [Ti(O^{n}Bu)$_4$], trimethylamine-*N*-oxide dihydrate (TAO·2H_2O), diphenyldisulfane, 1-bromoheptane, sodium formaldehyde sulfoxylate (rongalite), ammonium carbamate, [bis(acetoxy)iodo]benzene and borane-tetrahydrofuran complex (1.0 M in THF) were purchased from Energy Chemical and used as received. Dichloromethane (DCM) and toluene (Tol) were purified by the MBraun SPS system. HDPE was obtained from a plastic bottle bought from Titan.

2.3 Characterization methods: NMR spectra for the monomers and polymers were recorded on a Bruker AV400 (^{1}H: 400 MHz, ^{13}C: 100 MHz) or a Bruker AV500 (^{1}H: 500 MHz, ^{13}C: 125 MHz). The molecular weights (M_n) and molecular weight distributions ($Đ$) of polymers were measured utilizing gel permeation chromatography (GPC) with 1,2,4-trichlorobenzene containing 0.0125 wt% butylated hydroxytoluene (BHT) as inhibitor (1 mL min^{-1} at 150 °C) on a PL-GPC 220-type high-temperature chromatograph equipped with three PL-gel 10 μm Mixed-BLS type columns. Mass spectrometry of synthesized compounds was recorded on an Acquity UPLC & Quattro Premier. A series of low polydispersity polystyrene standards was used for calibration. Melting point temperature (T_m) of polymers were measured through DSC analyses using a Mettler TOPEM TM DSC Instruments under a nitrogen atmosphere at heating and cooling rates of 10 °C/min (temperature range: -80~180 °C). Thermogravimetric analyses (TGA) were performed on a METTLER TOLEDO TGA 2 thermal analyser at a heating rate of 10 °C/min (temperature range: 40~800 °C). Wide angle X-ray diffractograms (WAXD) were performed on a Rigaku SmartLab instrument. Analysis of water contact angles was performed on a Krüss Drop Shape Analyzer DSA100 with water. ATR-IR spectra were acquired on a Bruker VERTEX 70 Fourier Transform Infrared spectrometer.

2.4 Synthesis of the organoborane initiator

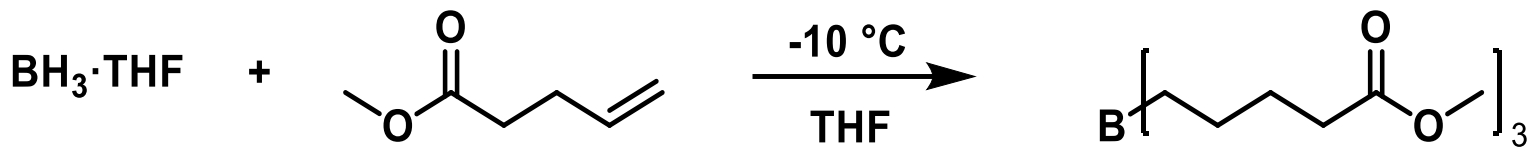

Preparation of tris(5-methoxy-5-oxopenty) borane

The compound was prepared according to the reported method.[1] A 100 mL round-bottom flask, dried overnight at 110 °C, was equipped with a stir bar. Under nitrogen (N_2) atmosphere, methyl 4-pentenoate (6.5 g, 57 mmol, 3.4 equiv) and tetrahydrofuran (THF, 72 mL) were introduced into the flask, which was cooled to 0 °C. Subsequently, a solution of $BH_3 \cdot THF$ (1.0 M in THF, 16.8 mL, 16.8 mmol) was added to the flask over 10 min. The reaction was allowed to stir at room temperature for 2 h. The resultant solution was stored at -20 °C under N_2 and used directly for polymerization without further processing.

2.5 Preparation of Monomer

2.5.1 Preparation of dimethylsulfoxonium chloride: The compound was prepared according to the reported method.[2]

2.5.2 Preparation of dimethylsulfoxonium methylide (C1): The compound was prepared according to the reported method.[2] Note that this compound is stored in a toluene solution. 1 mL of 4M $HCl \cdot Et_2O$ solution is added to 2 mL of the solution to obtain dimethylsulfoxonium chloride. After removing the solvent under reduced pressure, the concentration of the solution is determined based on the mass of dimethylsulfoxonium chloride.

Note that a small amount of methyl branches is incorporated into the polyethylene backbone during the polyhomologation of **C1**. This side reaction originates from the trace presence of the **C2** homolog

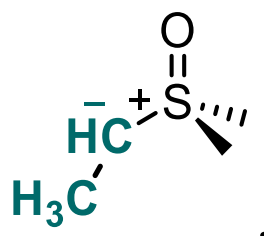

, which is formed as a minor byproduct during the synthesis of the **C1** monomer itself and is subsequently introduced during chain growth[3]. The formation of these methyl branches is confirmed by distinct resonances at 0.94 ppm in the ^{1}H NMR and 19.75 ppm in the ^{13}C NMR spectra.

2.5.3 Preparation of (dimethylamino)-*S*-phenylsulfoxonium heptyl ylide (C7)

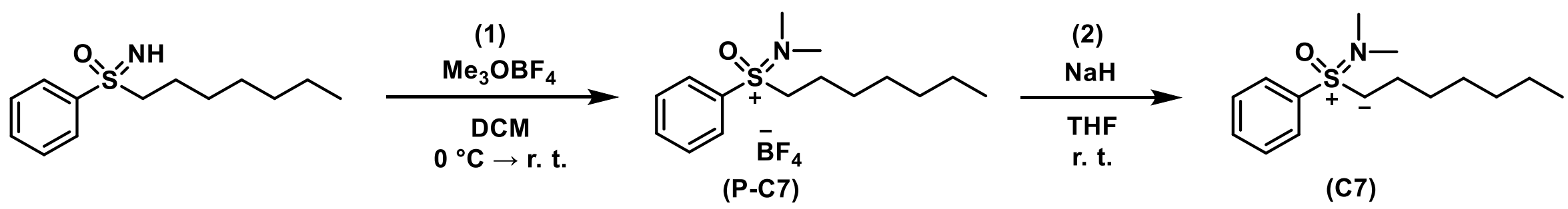

(1) Preparation of (dimethylamino)heptyl-*S*-phenylsulfoxonium tetrafluoroborate (**P-C7**)**:** The compound was prepared according to the reported method.[4] A 500 mL of round-bottom flask was equipped with a stir bar after being dried overnight at 110 °C. Then *S*-heptyl-*S*-phenylsulfoximine (12.0 g, 50 mmol, 1.0 equiv) and dichloromethane (DCM) (100 mL) were added to the flask. Trimethyloxonium tetrafluoroborate (8.1 g, 55 mol, 1.1 equiv) was added to the above reaction mixture at 0 °C. After 10 mins, the reaction was stirred at room temperature for 3 h and the excess Meerwein's salt was filtered off. The filtrate was made basic (pH 9) with 1 M NaOH and extracted with DCM (3×50 mL).The organic layer was dried over anhydrous Na_2SO_4, the crude product was obtained by removing the solvent under reduced pressure, and subsequently, the methylating process was repeated two more times to improve the yield. The product was recrystallized in EtOH and hexane.

The product was obtained by removing the solvent under reduced pressure as white needles (60% yield).

^{1}H NMR (500 MHz, 298 K, $CDCl_3$, 7.26 ppm): δ = 8.22-8.19 (m, 2H), 7.97-7.93 (m, 2H), 7.87-7.82 (m, 2H), 4.35-4.28 (m, 1H), 4.19-4.12 (m, 1H), 3.15 (s, 6H), 1.80-1.70 (m, 1H), 1.50-1.38 (m, 3H), 1.28-1.13 (m, 6H), 0.83 (t, 3H) ppm.

^{13}C{^{1}H}[5] NMR (125 MHz, 298 K, $CDCl_3$, 77.16 ppm): δ =137.55, 131.50, 129.89, 127.05, 51.70, 38.34, 31.27, 28.61, 27.58, 22.46, 22.27, 14.04 ppm.

MS (ESI$^+$): m/z: calcd for $C_{15}H_{26}NOS[M]^+$: 268.44, found 268.2.

MS (ESI$^-$): m/z: calcd for $BF_4[M]^-$: 86.80, found 86.6.

(2) Preparation of (dimethylamino)-*S*-phenylsulfoxonium heptyl ylide (**C7**): A 100 mL of round-bottom flask was equipped with a stir bar after being dried overnight at 110 °C. (Dimethylamino)heptyl-S-phenylsulfoxonium tetrafluoroborate (1 equiv) and THF were added to the flask, the NaH (3 equiv, which was washed with hexanes and dried under N_2) was added to the flask. Evolution of H_2 and the solution changes from colorless to bright yellow. The reaction was stirred at room temperature until no more H_2 releasing. The mixture was filtered through a Schlenk filter containing Celite 545 to remove $NaBF_4$ salt. The resulting solution was used directly for polymerization without purification.

2.6 General polymerization procedure

2.6.1 Preparation of the telechelic linear macromonomers

In a typical experiment for **C1** homopolymerization, a 100 mL of round-bottom flask was equipped with a stir bar after being dried overnight at 110 °C. Dimethylsulfoxonium methylide (dissolved in toluene, **C1**) was added to the flask under N_2 atmosphere and the flask was heated to 70 °C. Tris(5-methoxy-5-oxopenty) borane (dissolved in THF) was rapidly injected via syringe under N_2 into the flask. After approximately 20 min, when **C1** was fully consumed and polymerization was complete, trimethylamine-N-oxide dihydrate (TAO·$2H_2O$, typically 20 equiv. to borane) was added. The flask was then heated to 110 °C for 12 h. The reactor was cooled to room temperature. The reaction mixture was precipitated from methanol to isolate the telechelic PE macromonomer, which was then dried under vacuum to constant weight at 40 °C.

2.6.2 Preparation of the telechelic branched macromonomers

In a typical experiment for **C1** and **C7** copolymerization, a 100 mL of round-bottom flask was equipped with a stir bar after being dried overnight at 110 °C. Tris(5-methoxy-5-oxopenty) borane (dissolved in THF) was injected via syringe under N_2 into the flask, which was then heated to 70 °C. (Dimethylamino)-S-phenylsulfoxonium heptyl ylide (**C7**) and **C1** were alternatively and slowly added in dropwise fashion. Upon completion of the addition, the polymerization process was finished, then trimethylamine-N-oxide dihydrate (TAO·$2H_2O$, typically 20 equiv. to borane) was added to the flask, which was subsequently heated to 110 °C. After 12 h, the reactor was then cooled to room temperature. The reaction mixture was precipitated in methanol to isolate the telechelic POE macromonomer, which was then dried under vacuum to constant weight at 40 °C.

2.7 General polymerization and repolymerization procedure

In a typical experiment, a 50 mL of round-bottom flask was equipped with a stir bar after being dried overnight at 110 °C. The telechelic PE macromonomer (1.0 g) and $Ti(O^nBu)_4$ (0.05 mol %, 1 mM in toluene) were introduced into the flask under N_2 atmosphere. The mixture was heated to 180 °C and then applied to a high vacuum. After 18 h, the flask was cooled to 110 °C and the reaction mixture

was dissolved in 50 mL of toluene. The polymer was then precipitated from methanol and subsequently dried under vacuum to constant weight at 40 °C.

2.8 General degradation procedure

In a typical experiment, a 100 mL of glass pressure reactor was equipped with a stir bar after being dried overnight at 110 °C. The polymer sample (1 g), KOH (0.5 g), 2,6-di-*tert*-butyl-4-methylphenol (BHT) (20 mg), MeOH (10 mL) and toluene (20 mL) were then added to the reactor. Then the reactor was heated to 120 °C for 12 h, then allowed to cooled to room temperature. The reaction mixture was acidified with a concentrated HCl solution, precipitated in methanol to isolate the recovered telechelic PE macromonomer, which was then dried under vacuum to constant weight at 40 °C.

2.9 Degradation of *d*(*r*-OBC) and separation of the solid and oil fractions

In a typical experiment, a 100 mL of glass pressure reactor was equipped with a stir bar after being dried overnight at 110 °C. The polymer sample (1 g), KOH (0.5 g), 2,6-di-*tert*-butyl-4-methylphenol (BHT) (20 mg), MeOH (10 mL) and toluene (20 mL) were then added to the reactor. Then the reactor was heated to 120 °C for 12 h, then allowed to cooled to room temperature. The reaction mixture was acidified with HCl·Et_2O solution and poured it into the hexane, **MM_{tPE}** precipitates out while **MM_{tPOE}** dissolves in the hexane. The two substances were separated by filtration. The precipitate was washed twice with hexane. The solution was then dried to obtain **MM_{tPOE}**. KCl was removed by washing with a mixture of water and ethanol to obtain **MM_{tPE}**, which was then dried under vacuum to constant weight at 40 °C.

3. Supplementary Tables

Table S1. Synthesis of polyolefins with in-chain ester group (*d*PO) from macromonomers[a]

*d*PO	telechelic polyethylene	X_{COO} (mol%)[b]	$M_{n,GPC}$ (kDa)[c]	$M_{w,GPC}$ (kDa)[c]	$Đ$[c]
***d*PE-2.7%**	**$MM_{tPE\text{-}1K}$**	2.7	29	120	4.20
***d*PE-0.9%**	**$MM_{tPE\text{-}3K}$**	0.9	101	172	1.71
***d*PE-0.3%**	**$MM_{tPE\text{-}10K}$**	0.3	224	425	1.90

[a] Conditions: A toluene solution of Ti(O^{n}Bu)$_4$ (0.05 mol%) was added, then the temperature was raised to 180 °C. Typically, the polymerization was conducted at 180 °C under vacuum for 18 h. [b] Calculated by ^{1}H NMR analysis. [c] Determined by GPC in 1,2,4-trichlorobenzene at 150 °C.

4. Supplementary Figures

4.1 Representative GPC traces of Macromonomer and *d*PO

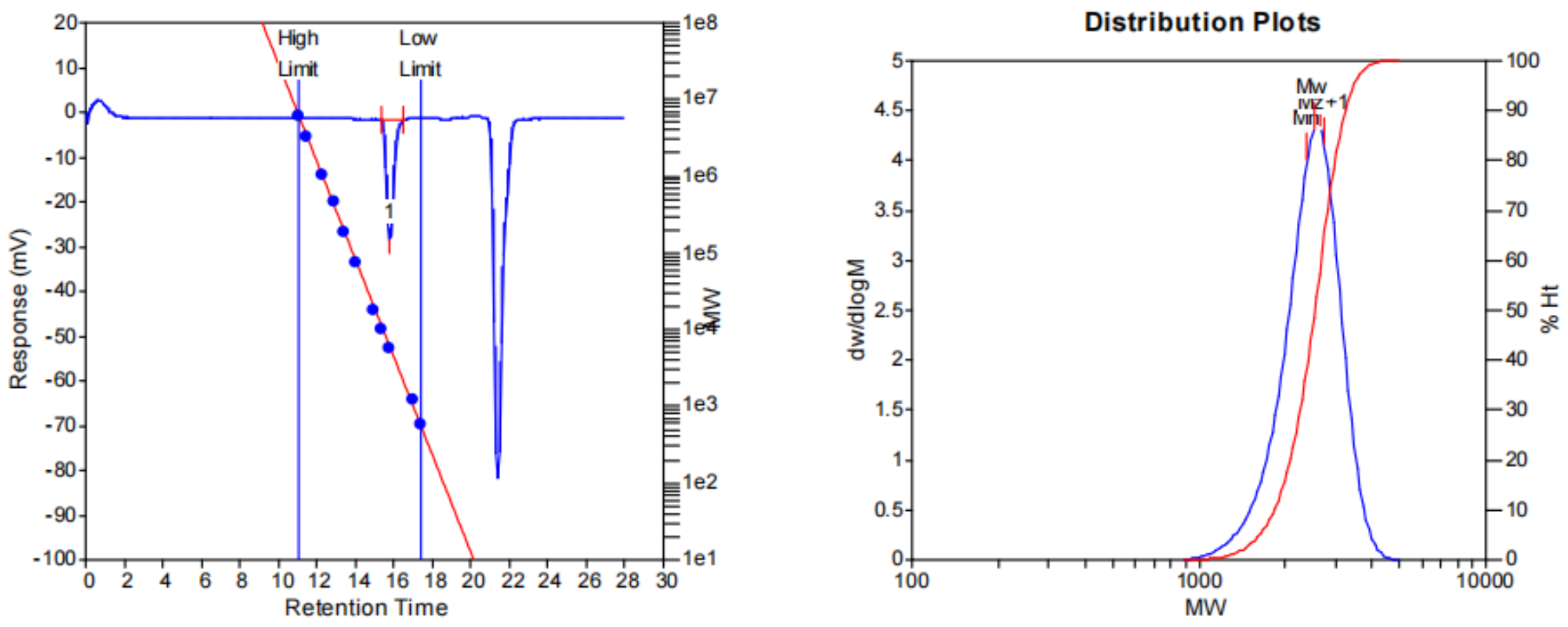

MW Averages

Peak No	Mp	Mn	Mw	Mz	Mz+1	Mv	PD
1	2564	2376	2506	2627	2741	2489	1.05471

Figure S1. GPC trace of $\mathbf{MM}_{t\mathbf{PE\text{-}3K}}$ in table 2.

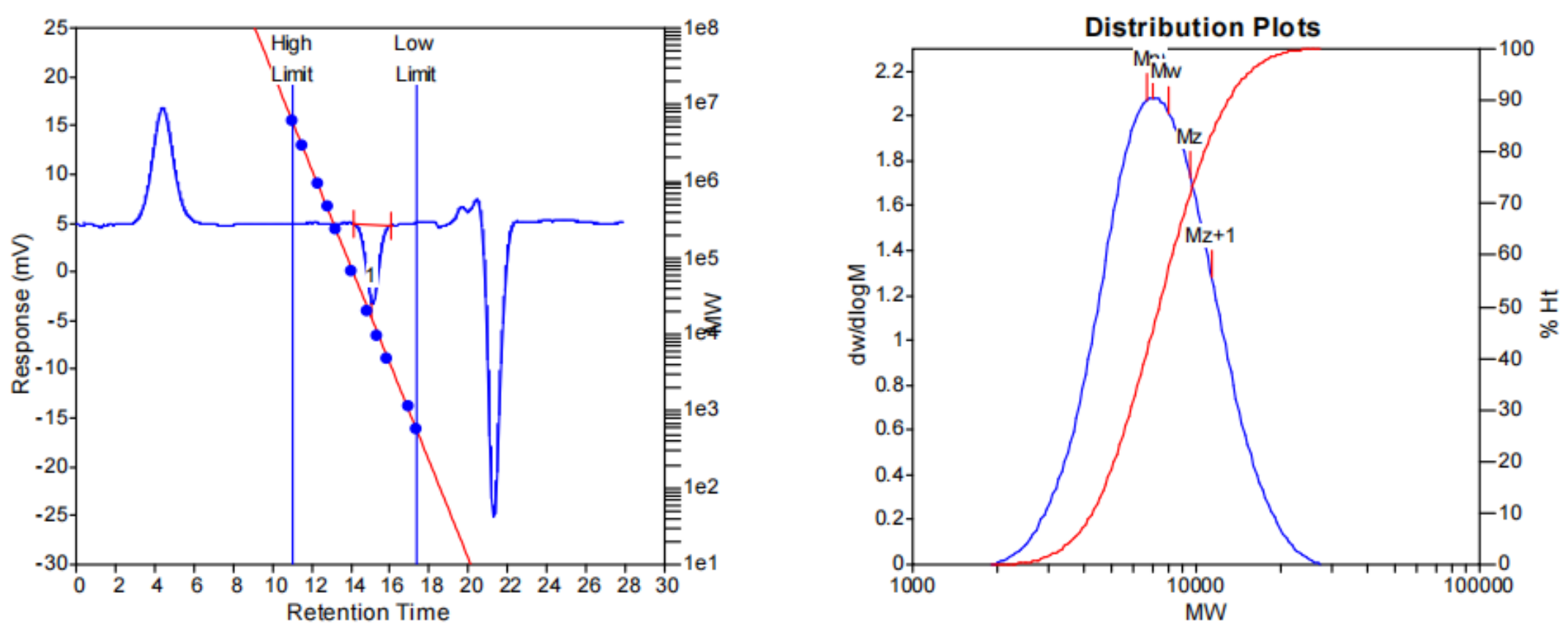

MW Averages

Peak No	Mp	Mn	Mw	Mz	Mz+1	Mv	PD
1	6992	6693	8021	9588	11329	7824	1.19842

Figure S2. GPC trace of $\mathbf{MM}_{t\mathbf{PE\text{-}7K}}$ in table 2.

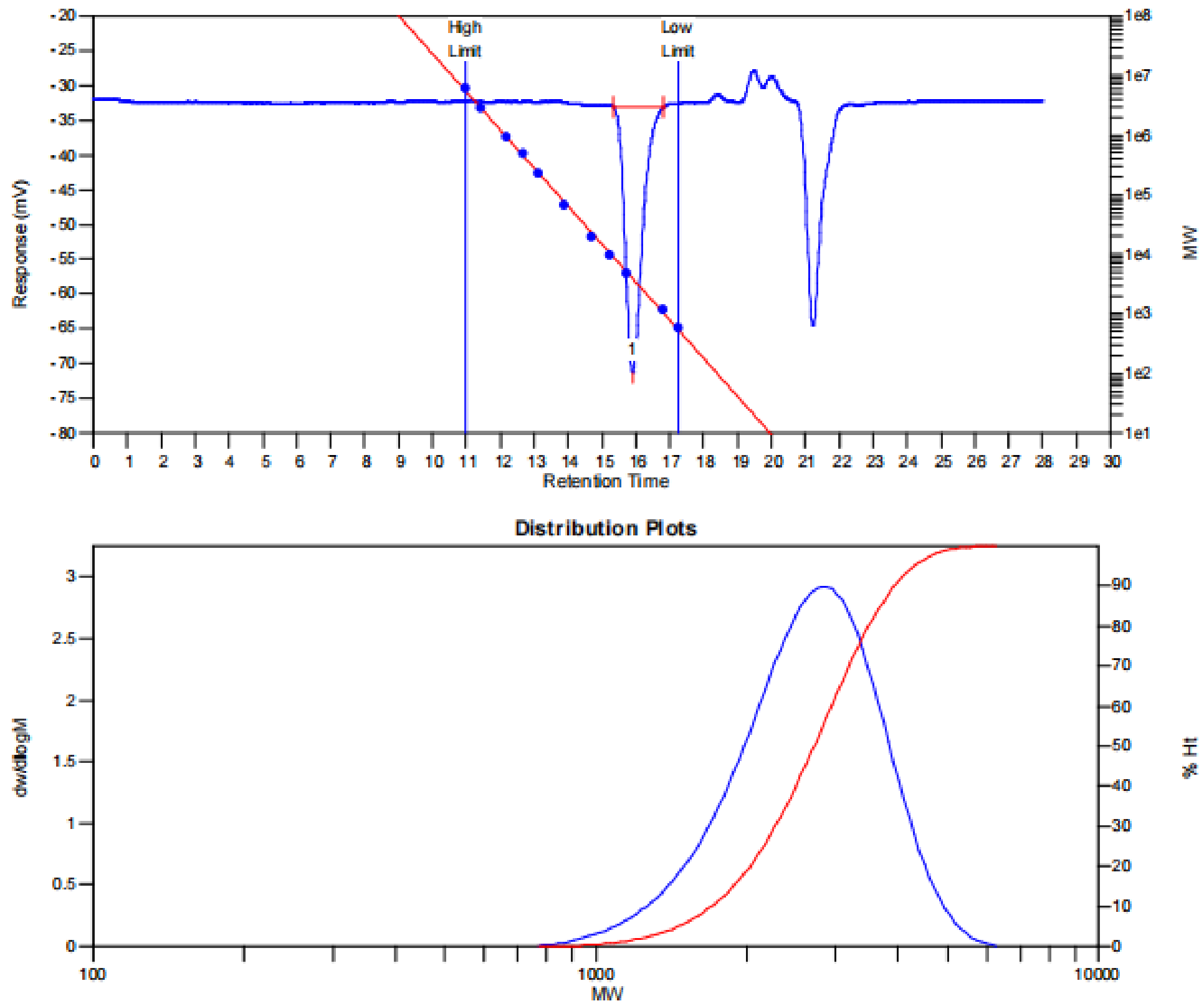

MW Averages

Peak No	Mp	Mn	Mw	Mz	Mz+1	Mv	PD
1	2813	2470	2748	3012	3261	2712	1.11255

Figure S3. GPC trace of **MM**$_{t\mathrm{POE\text{-}2K(6.7\%)}}$ in table 2.

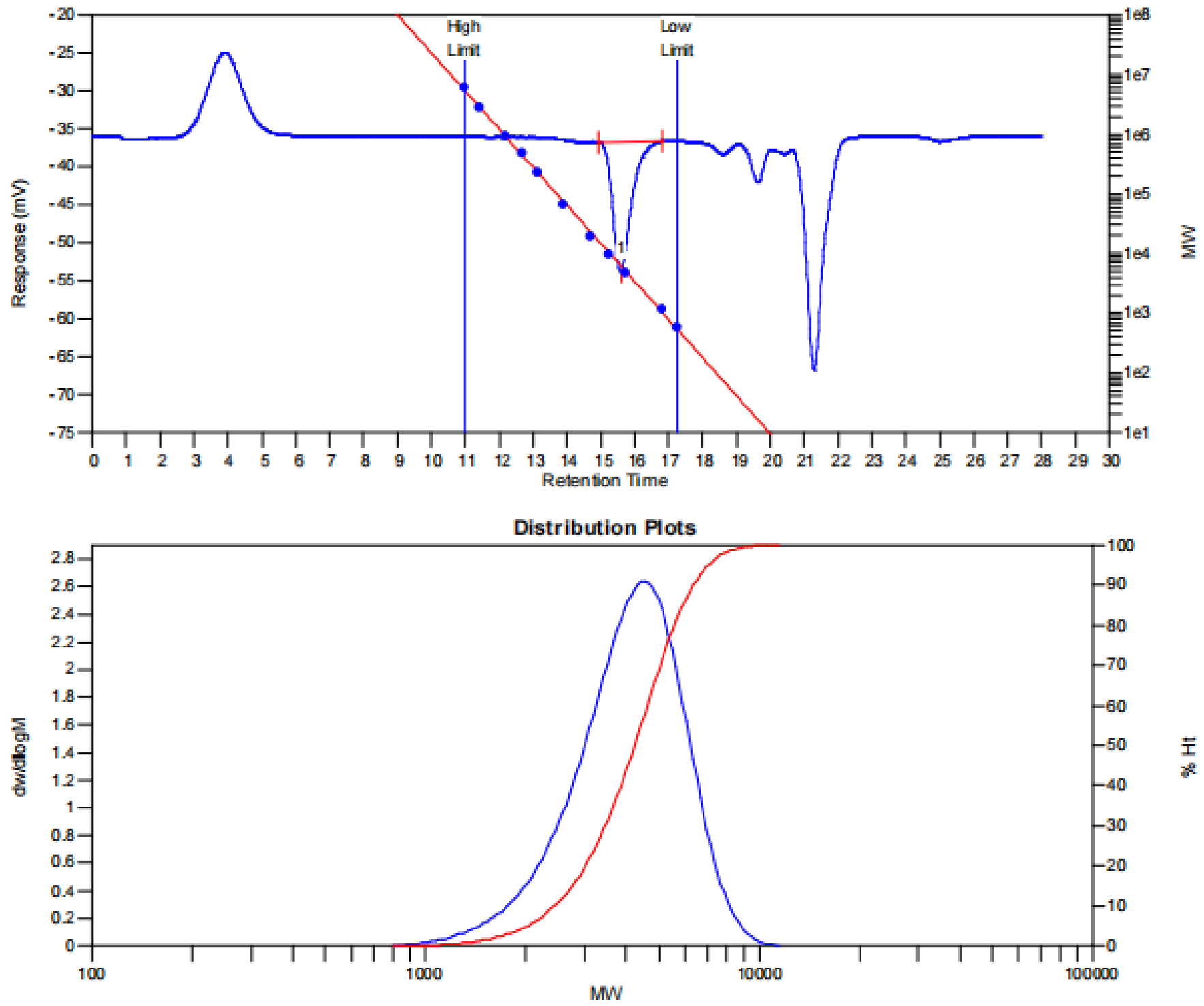

MW Averages

Peak No	Mp	Mn	Mw	Mz	Mz+1	Mv	PD
1	4546	3713	4287	4815	5304	4214	1.15459

Figure S4. GPC trace of **MM**$_{t\mathrm{OBC\text{-}4K(8.9\%)}}$ in table 2.

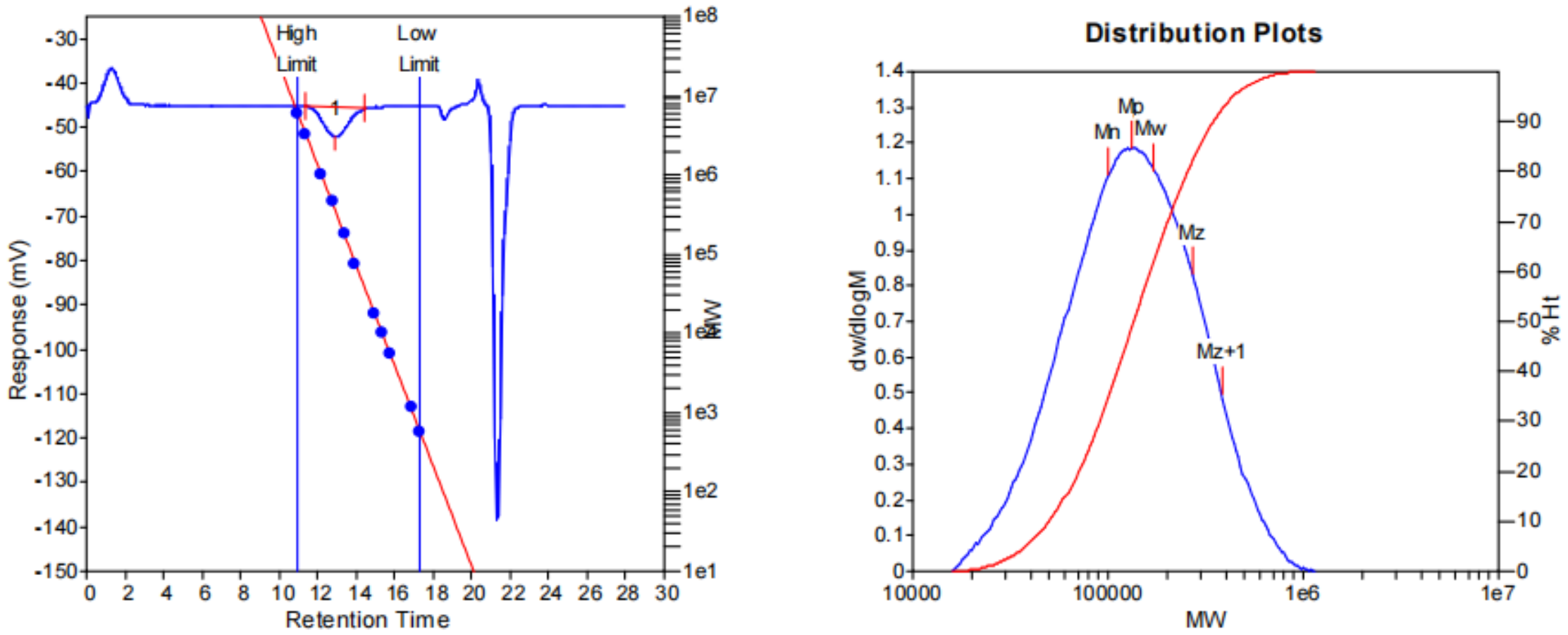

MW Averages

Peak No	Mp	Mn	Mw	Mz	Mz+1	Mv	PD
1	131771	100459	171476	271505	383701	159981	1.70693

Figure S5. GPC trace of ***d*PE-0.9%** in table S1.

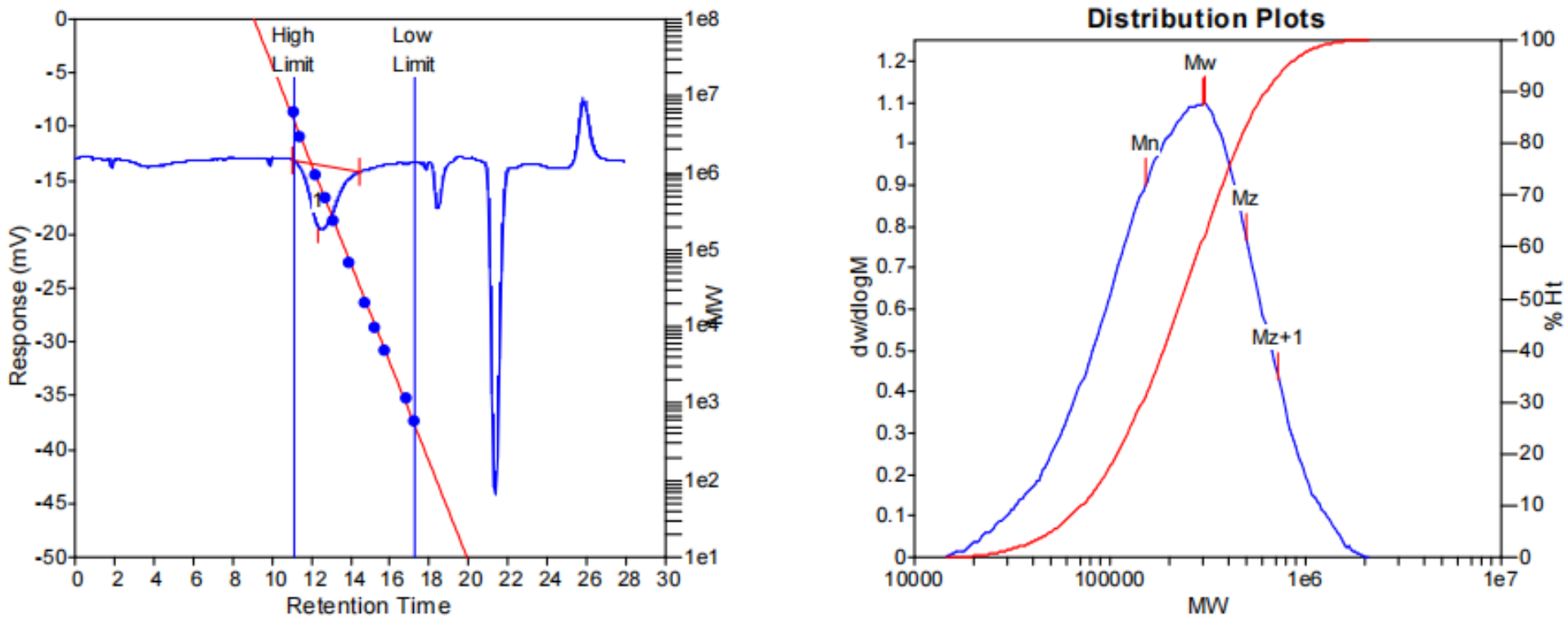

MW Averages

Peak No	Mp	Mn	Mw	Mz	Mz+1	Mv	PD
1	301948	150548	299729	503016	725692	276306	1.99092

Figure S6. GPC trace of ***d*PE-0.4%** in table 3.

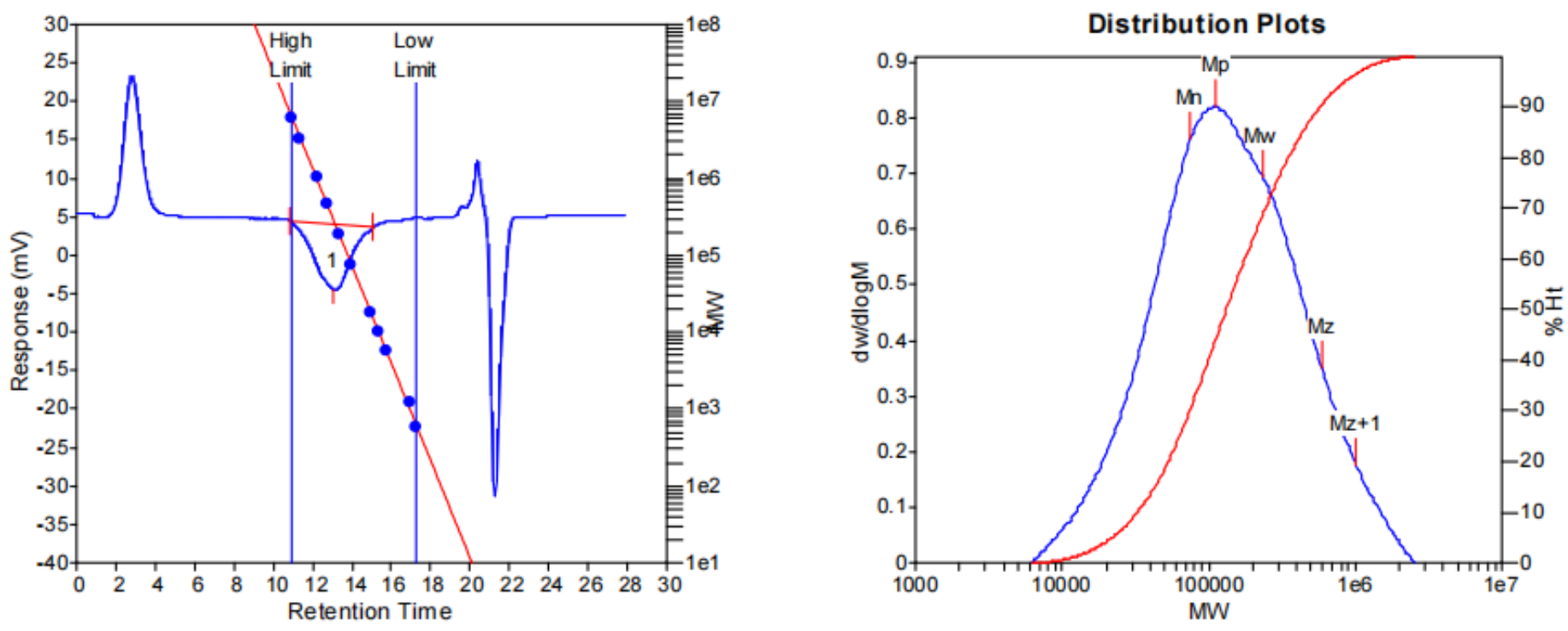

MW Averages

Peak No	Mp	Mn	Mw	Mz	Mz+1	Mv	PD
1	111769	75011	235006	593657	1013743	202301	3.13295

Figure S7. GPC trace of ***d*POE-1.6%** in table 3.

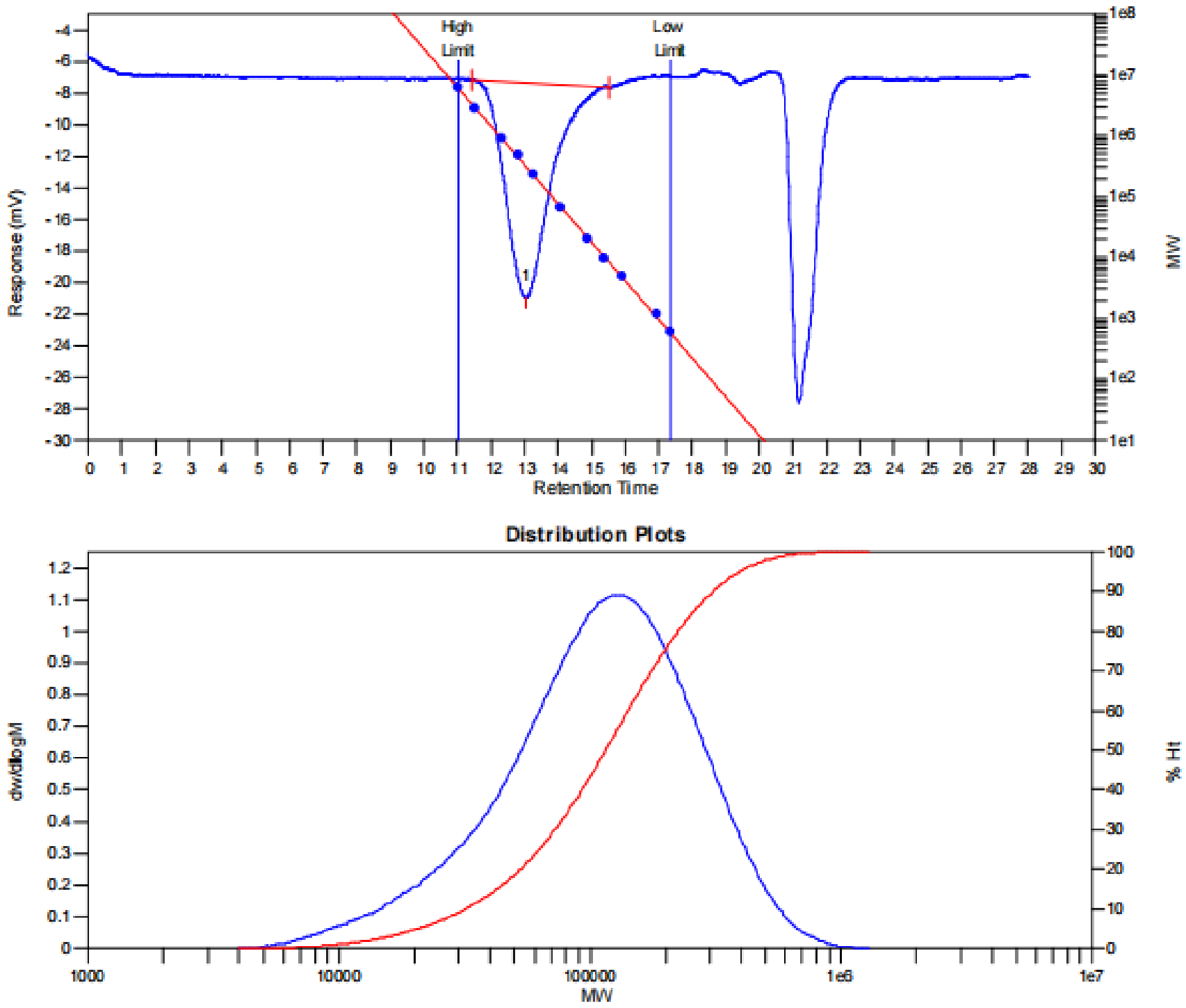

MW Averages

Peak No	Mp	Mn	Mw	Mz	Mz+1	Mv	PD
1	130790	67916	147338	248971	362214	135500	2.16942

Figure S8. GPC trace of ***d*(*r*-OBC-0.6%)** in table 3.

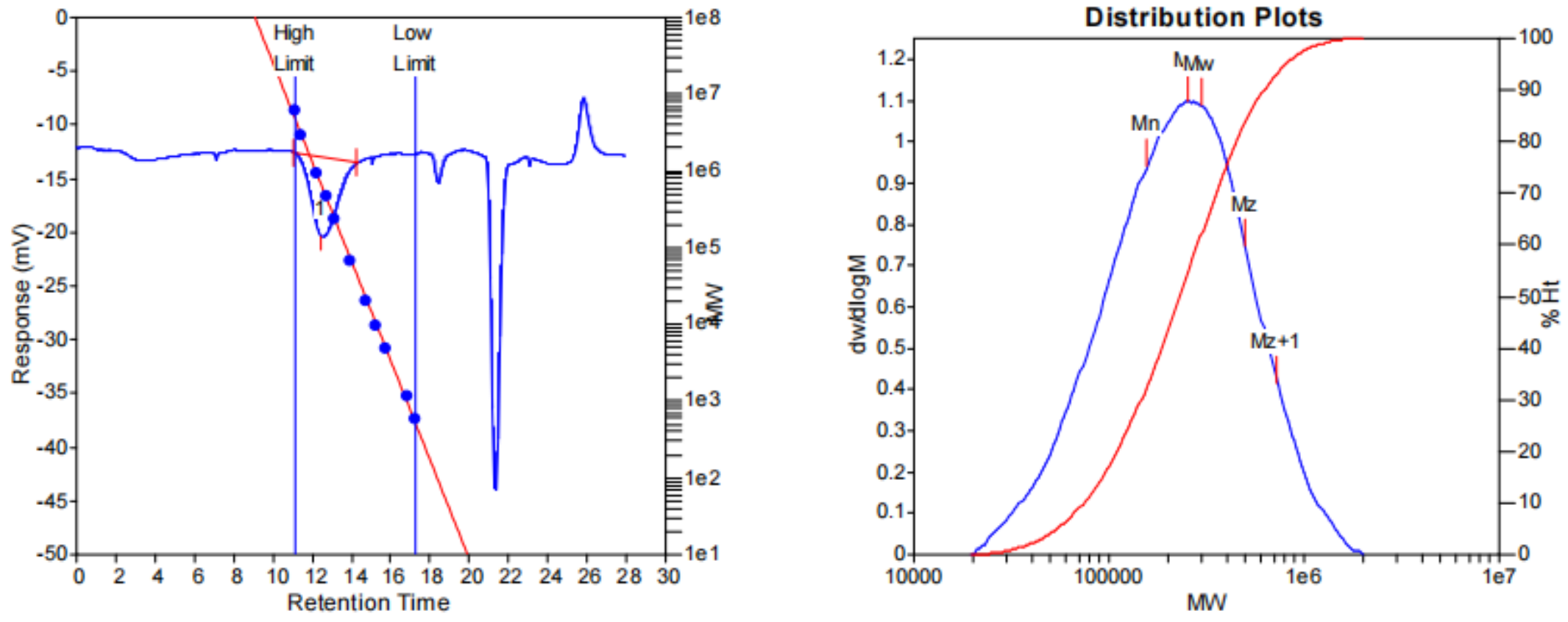

MW Averages

Peak No	Mp	Mn	Mw	Mz	Mz+1	Mv	PD
1	255178	156367	298765	502102	729553	275704	1.91067

Figure S9. GPC trace of ***d*PE-0.4%-RP-1** in Figure S52

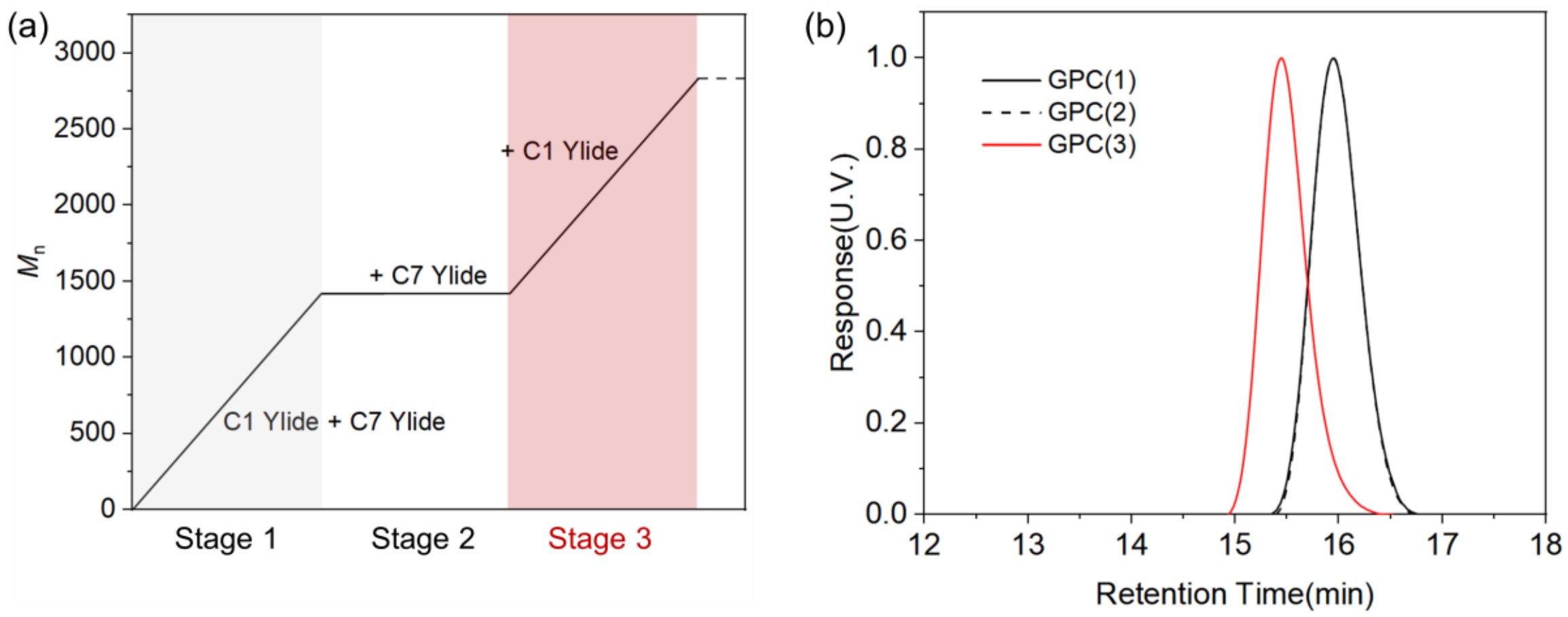

Figure S10. Co-polyhomologation of **C1** and **C7**. (a) Molecular weight evolution; (b) GPC traces recorded at three stages. Stage 1: Alternate slow dropwise addition of **C7** and **C1** (GPC trace 1). Stage 2: Further addition of **C7** only (GPC trace 2). Stage 3: Final dropwise addition of **C1** monomer (GPC trace 3).

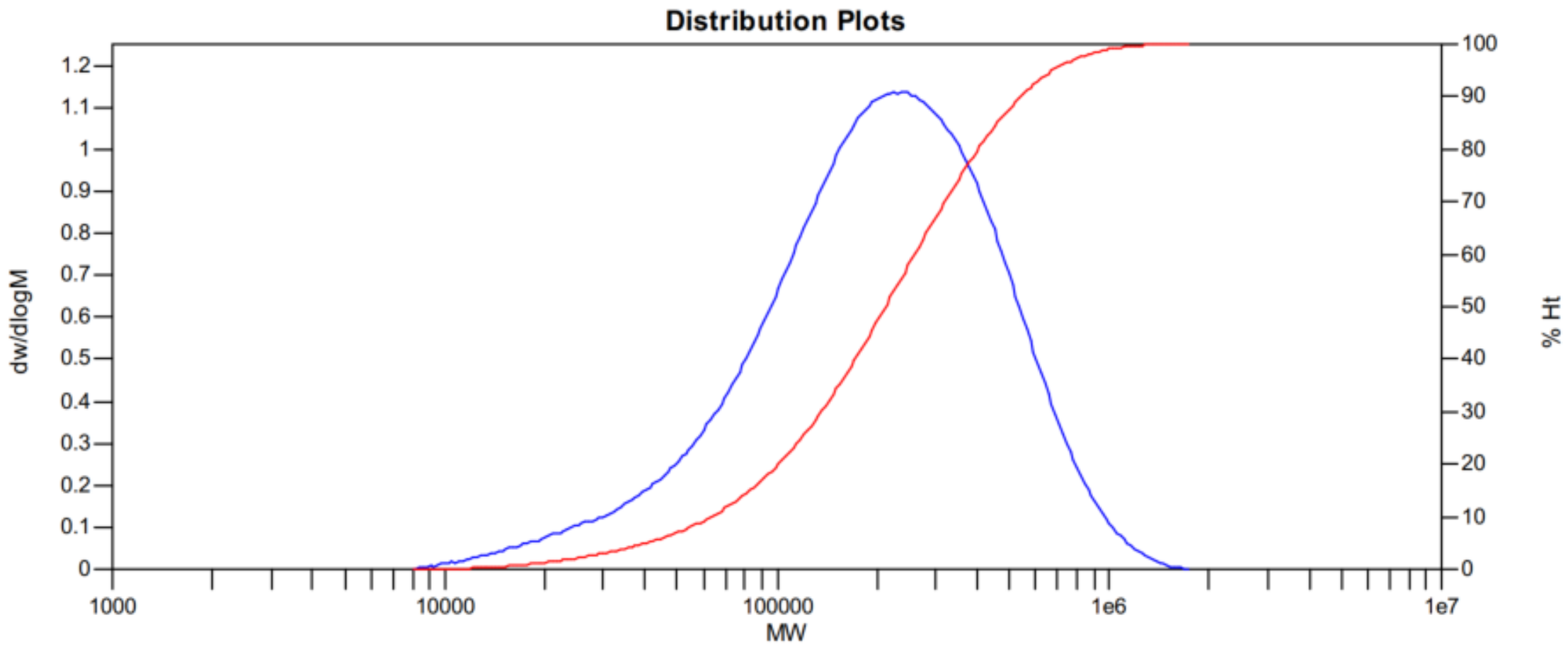

MW Averages

Peak No	Mp	Mn	Mw	Mz	Mz+1	Mv	PD
1	236295	127108	263083	421869	585808	243772	2.06976

Figure S11. GPC trace of HDPE in Figure 3b.

4.2 ESI-MS of Monomers

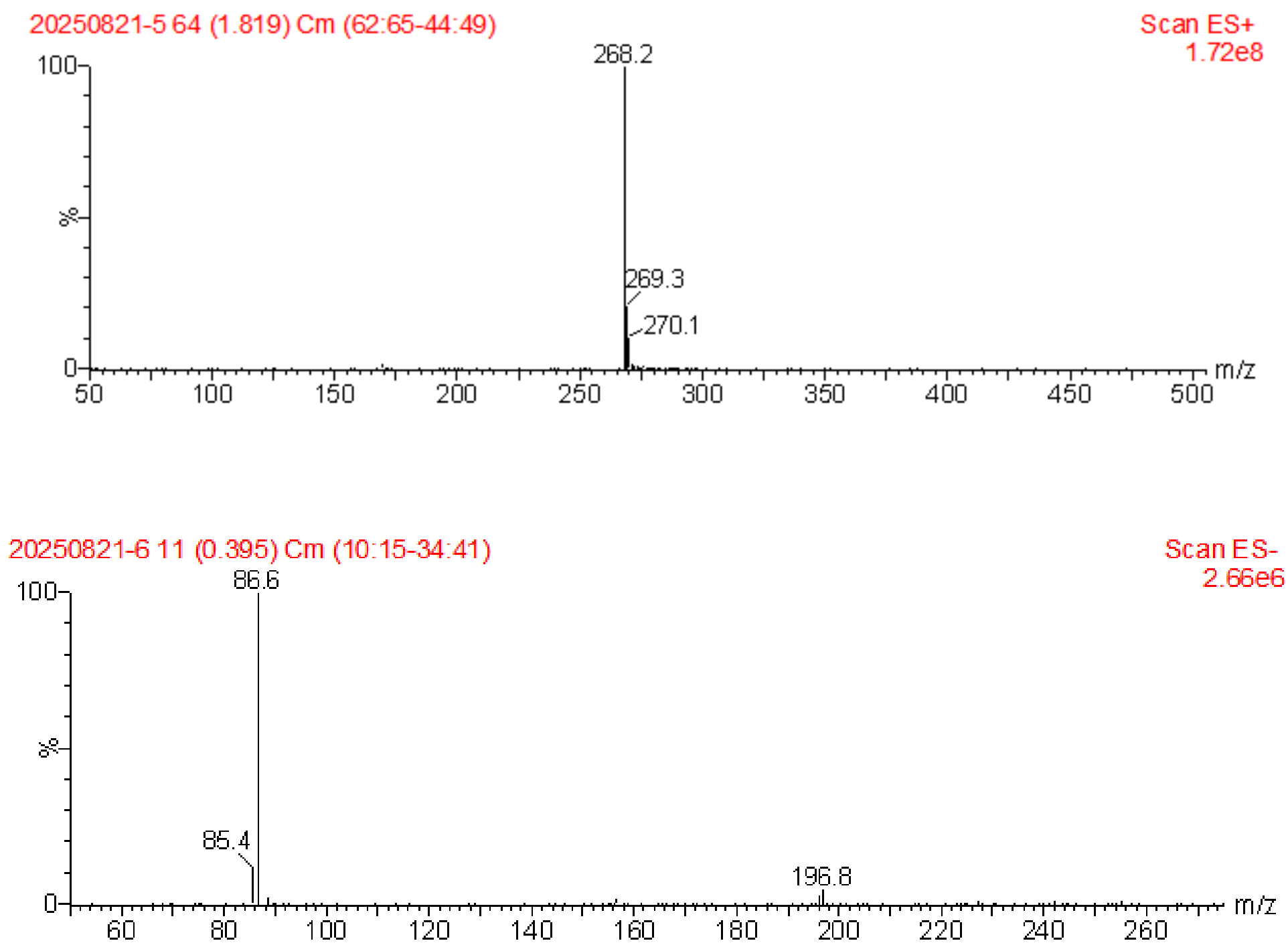

Figure S12. ESI-MS data of (dimethylamino)heptyl-*S*-phenylsulfoxonium tetrafluoroborate **MS (ESI$^+$)**: m/z = 268.2; **MS (ESI$^-$)**: m/z = 86.6.

4.3 NMR spectra of monomers

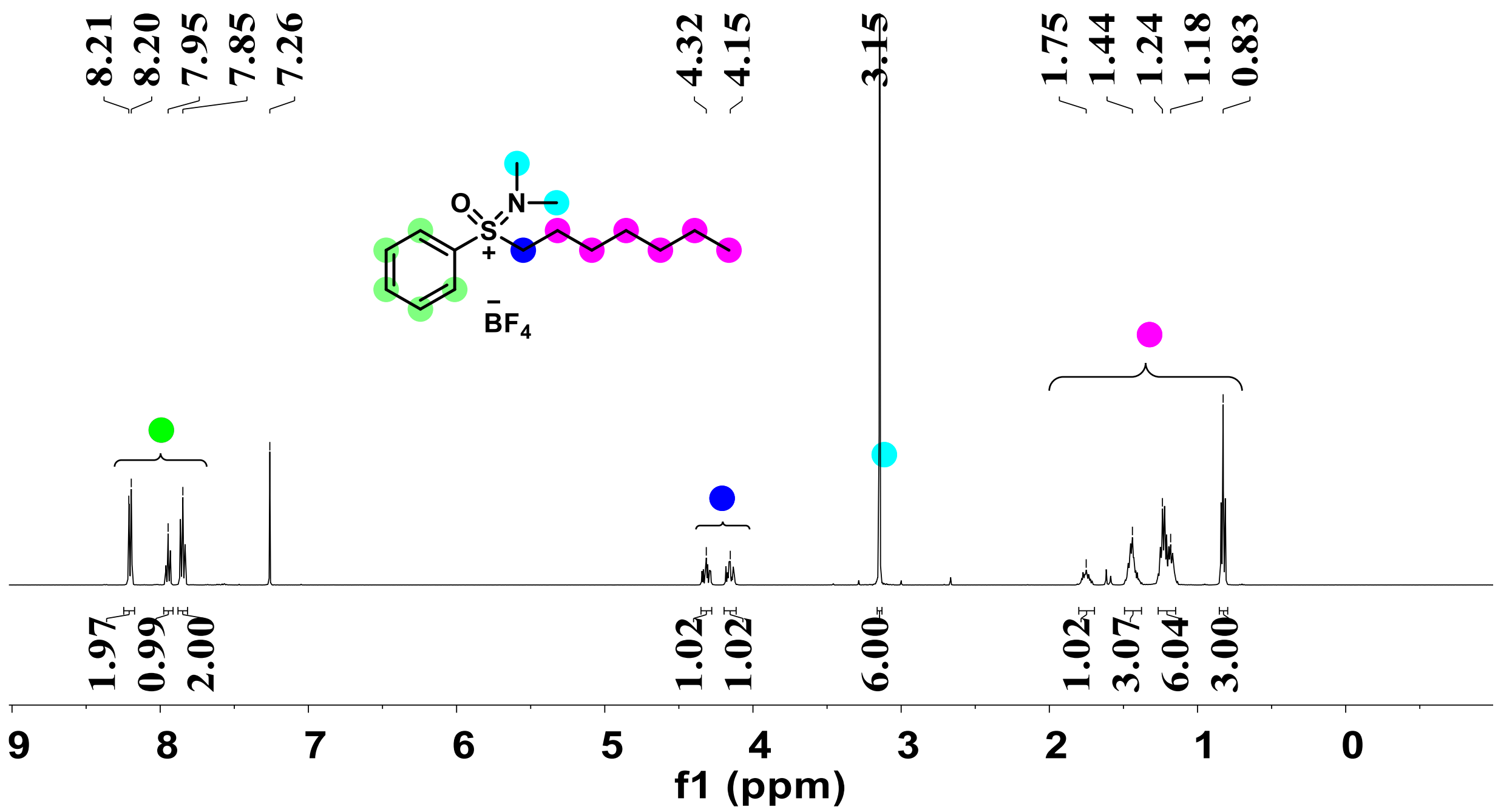

Figure S13. ^{1}H NMR spectrum of (dimethylamino)heptyl-*S*-phenylsulfoxonium tetrafluoroborate in $CDCl_3$ at 25 °C.

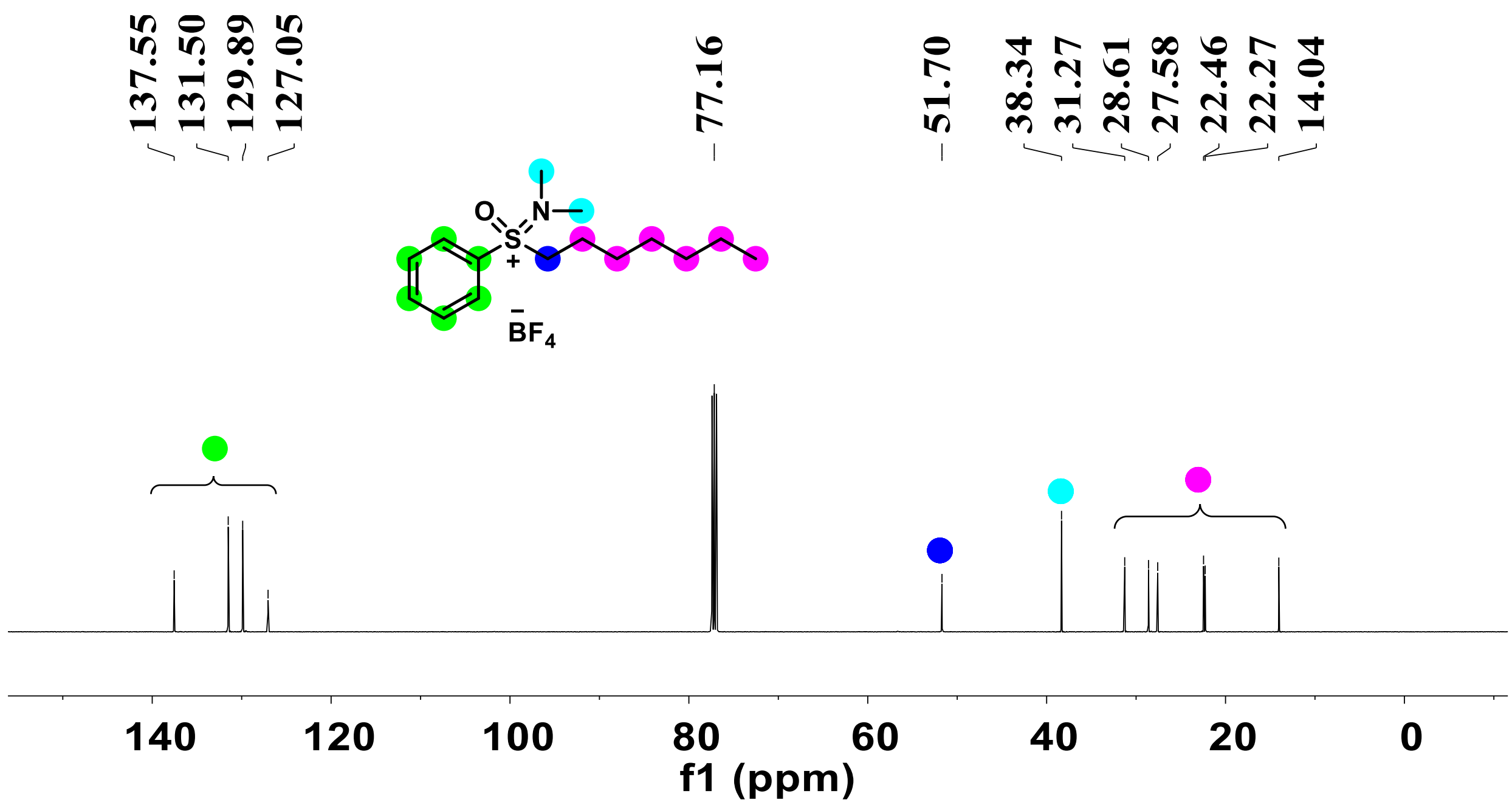

Figure S14. ^{13}C NMR spectrum of (dimethylamino)heptyl-*S*-phenylsulfoxonium tetrafluoroborate in $CDCl_3$ at 25 °C.

4.4 NMR spectra of Macromonomer

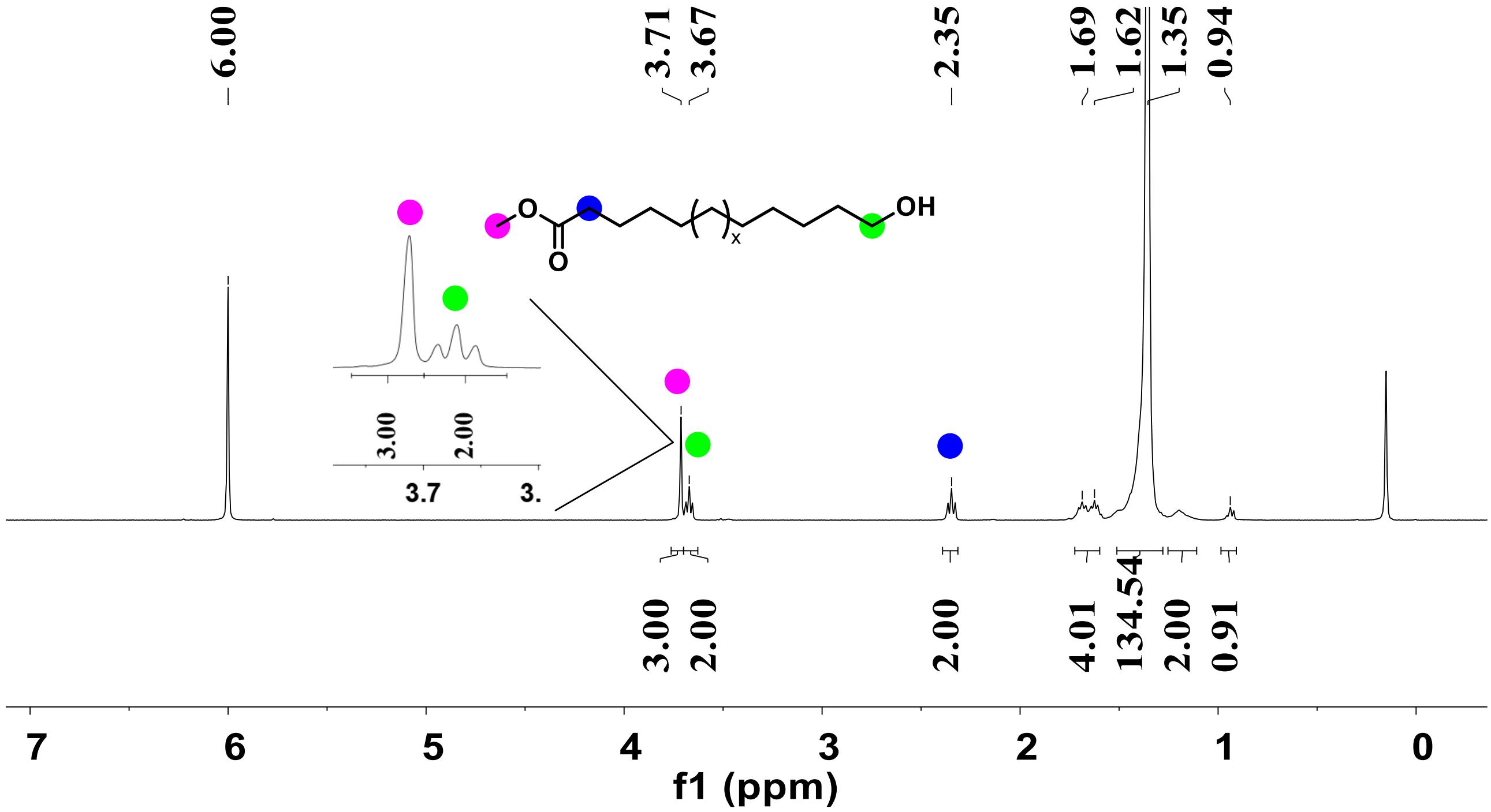

Figure S15. ^{1}H NMR spectrum of **MM$_{tPE\text{-}1K}$** in $C_2D_2Cl_4$ at 110 °C.

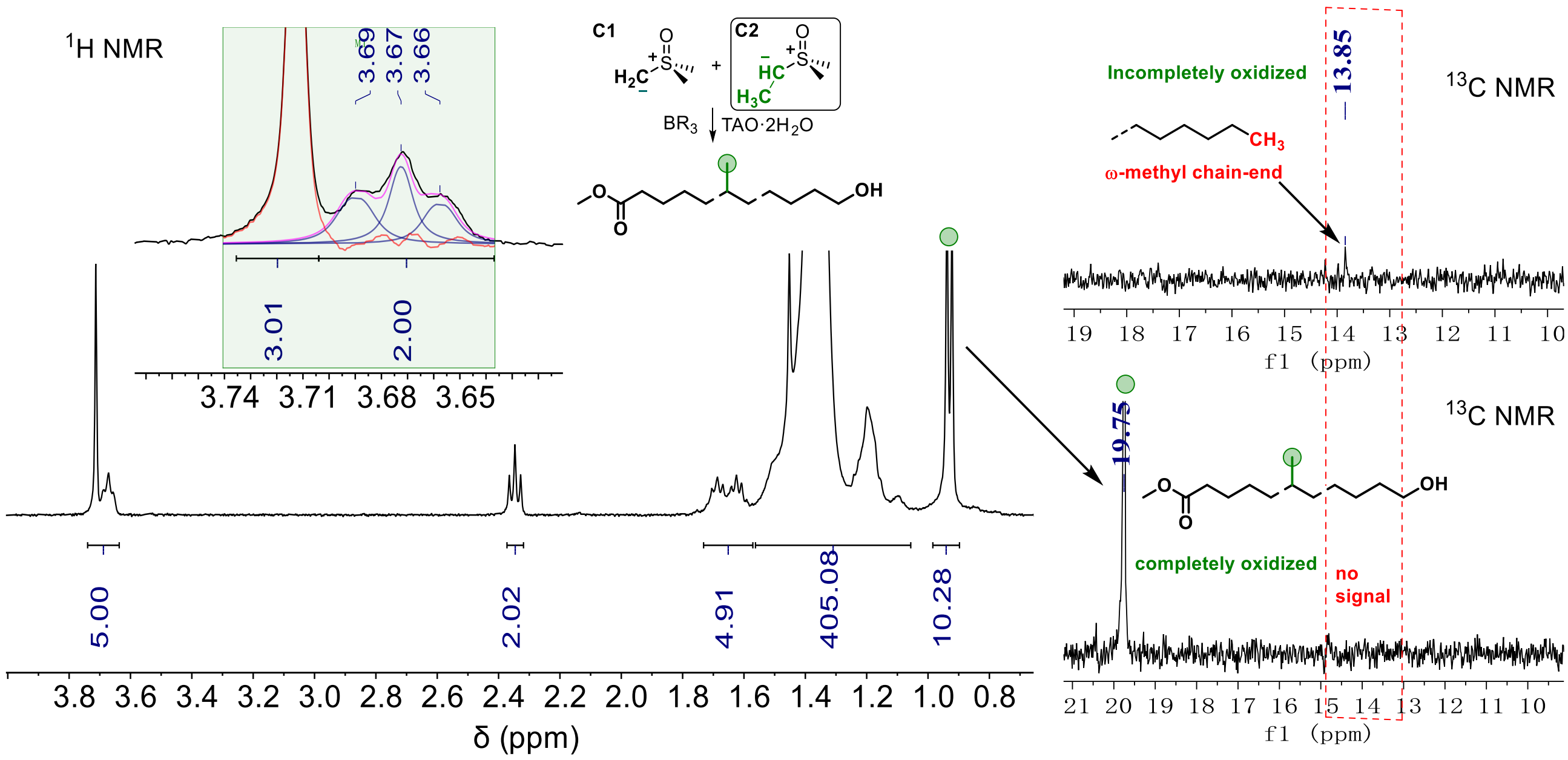

Figure S16. ^{1}H NMR spectrum of **MM$_{tPE\text{-}3K}$** in $C_2D_2Cl_4$ at 110 °C. (Note that a small amount of methyl branches is incorporated into the polyethylene backbone during the polyhomologation of **C1**. This side reaction originates from the trace presence of the **C2**, which is formed as a minor byproduct during the synthesis of the **C1**[3])

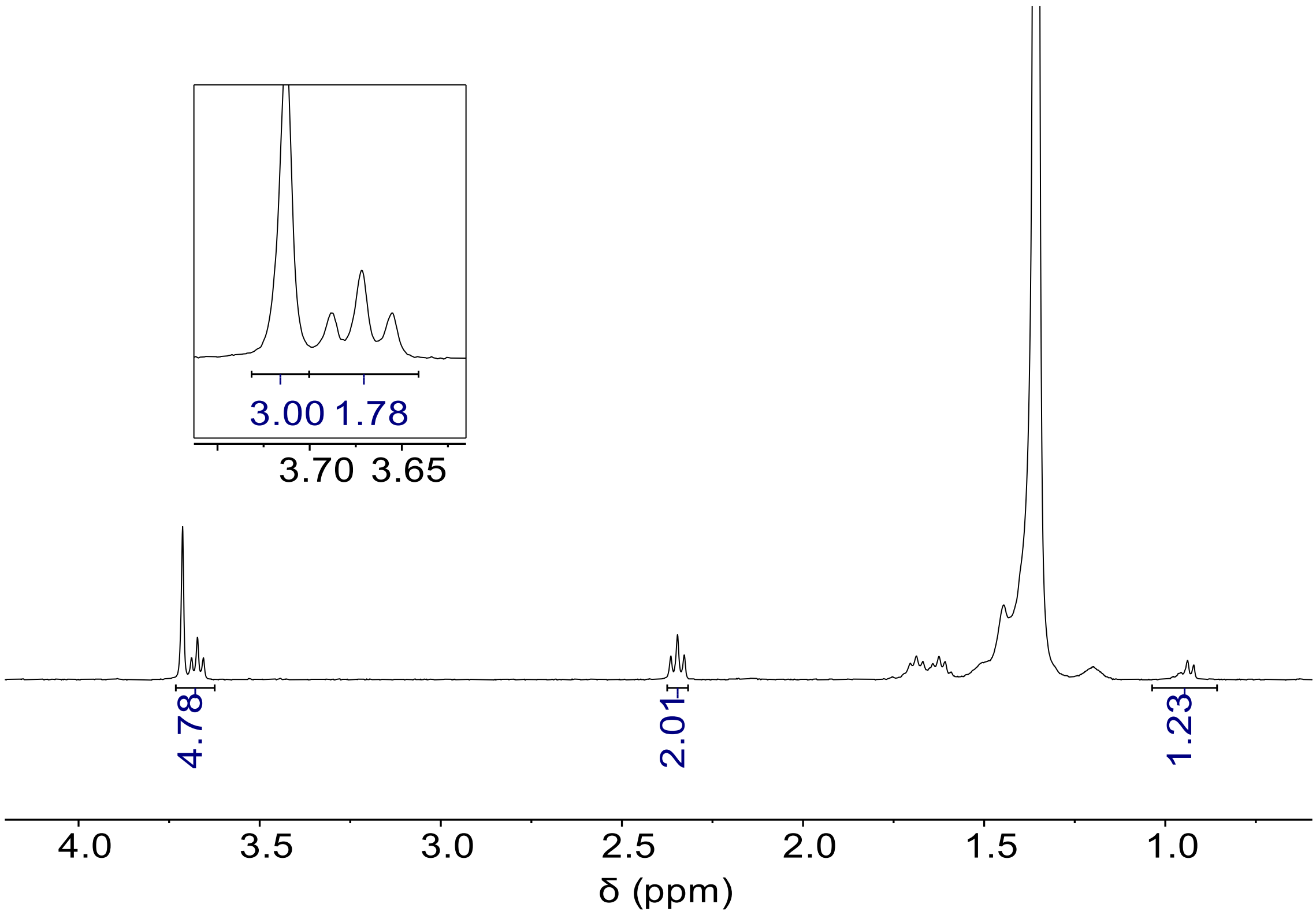

Reference: ^{1}H NMR spectrum ($C_2D_2Cl_4$ at 110 °C) of telechelic PE (**MM*t*PE**) obtained after incomplete oxidation (~90 %).

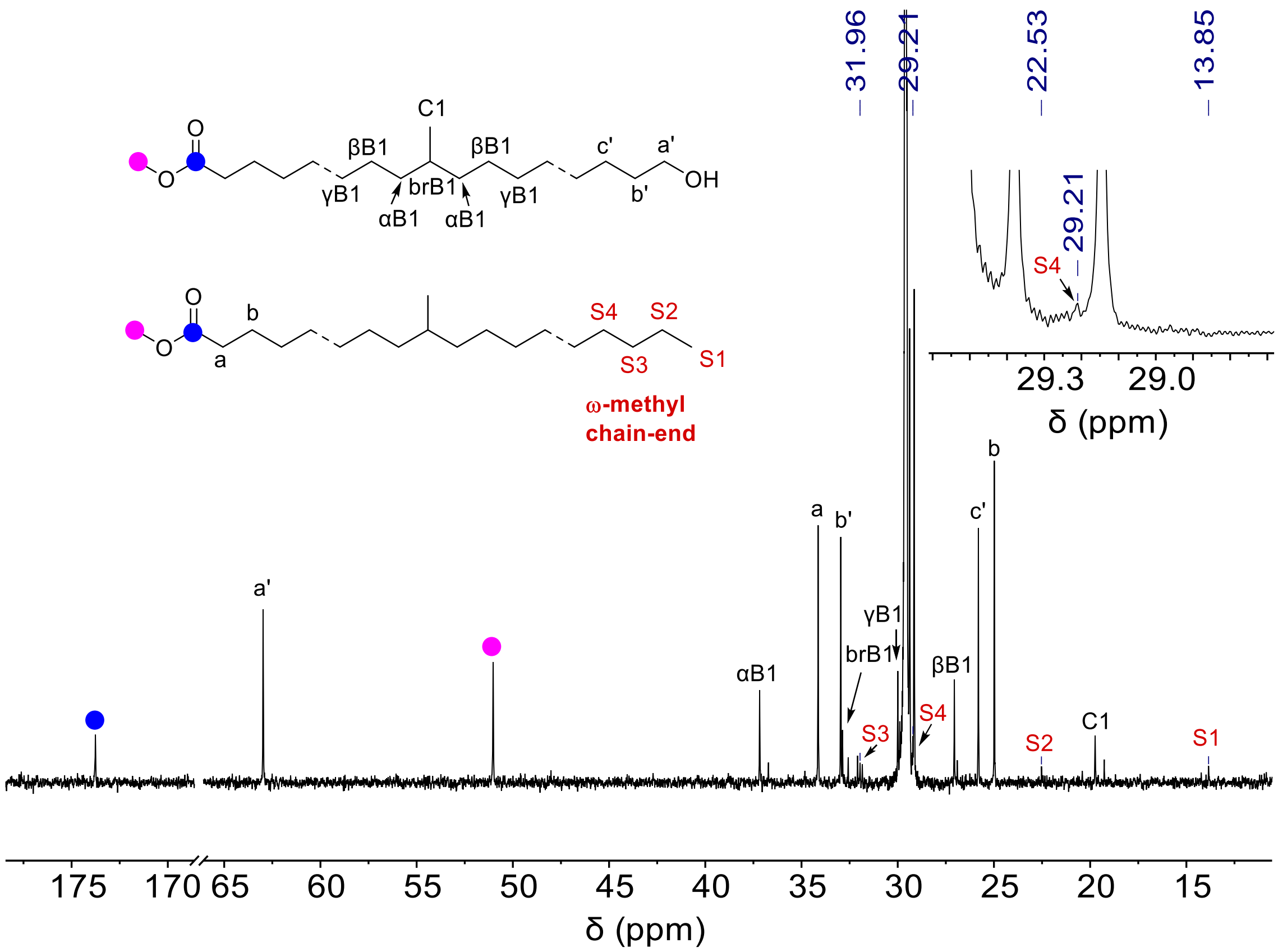

Reference: ^{13}C NMR spectrum ($C_2D_2Cl_4$ at 110 °C) of telechelic PE (**MM*t*PE**) obtained after incomplete oxidation (~90 %). (^{13}C NMR spectrum of incompletely oxidized **MM*t*PE** in Figure S14 and Figure 2b)

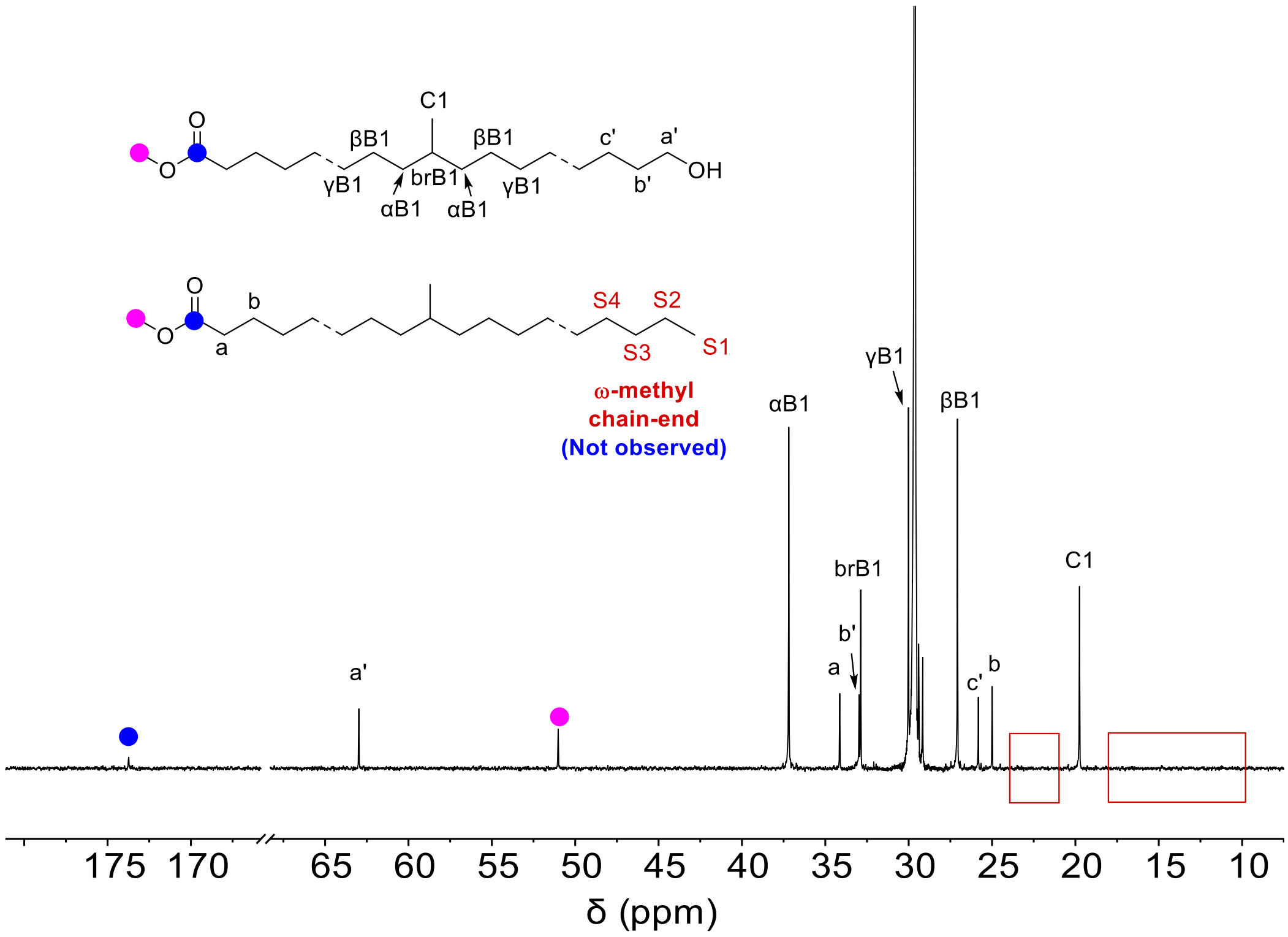

Reference: ^{13}C NMR spectrum ($C_2D_2Cl_4$ at 110 °C) of telechelic PE (**MM$_{tPE}$**) obtained after complete oxidation (>99 %). (Figure 2b).

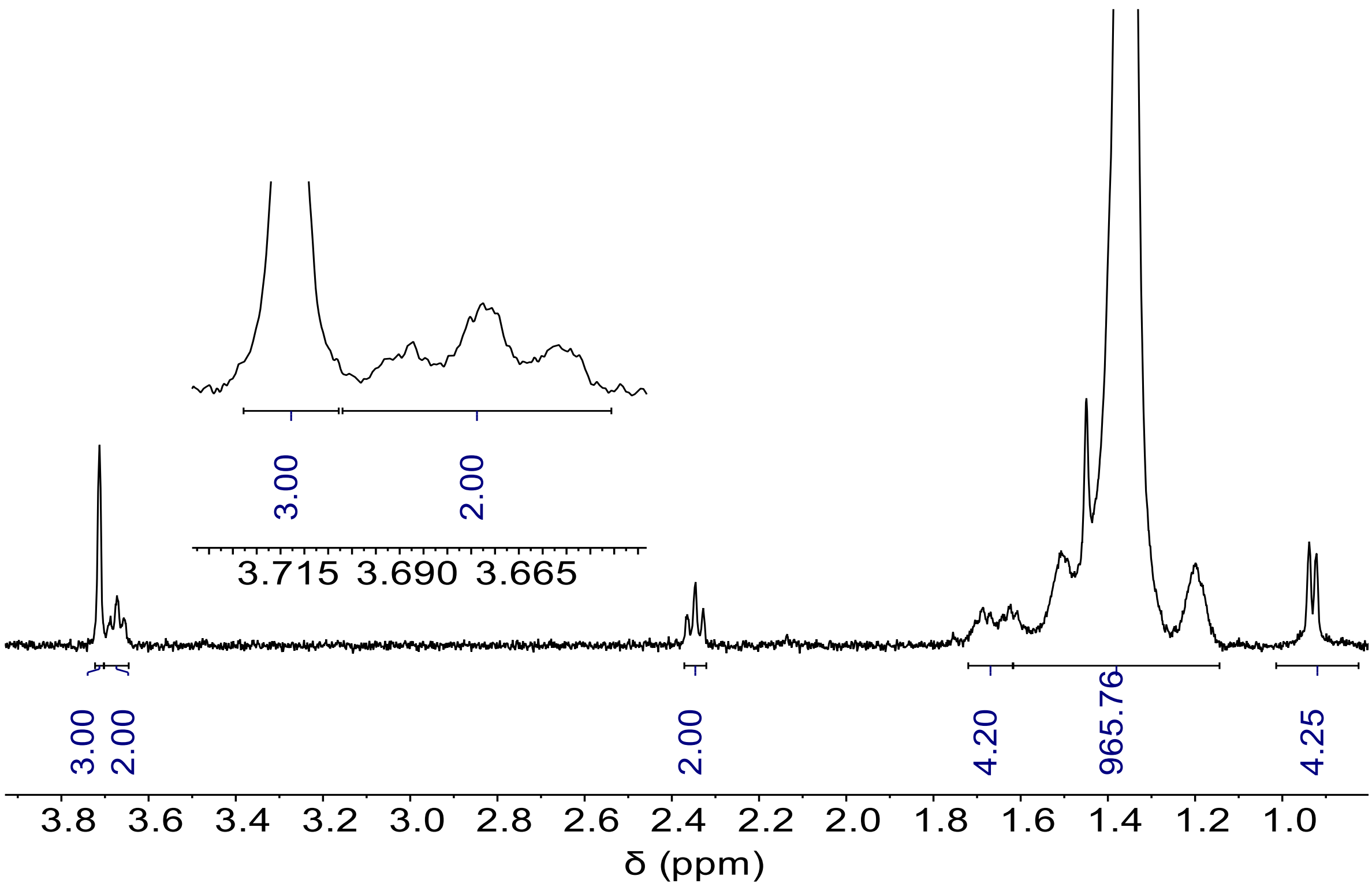

Figure S17. ^{1}H NMR spectrum of **MM$_{tPE\text{-}7K}$** in $C_2D_2Cl_4$ at 110 °C.

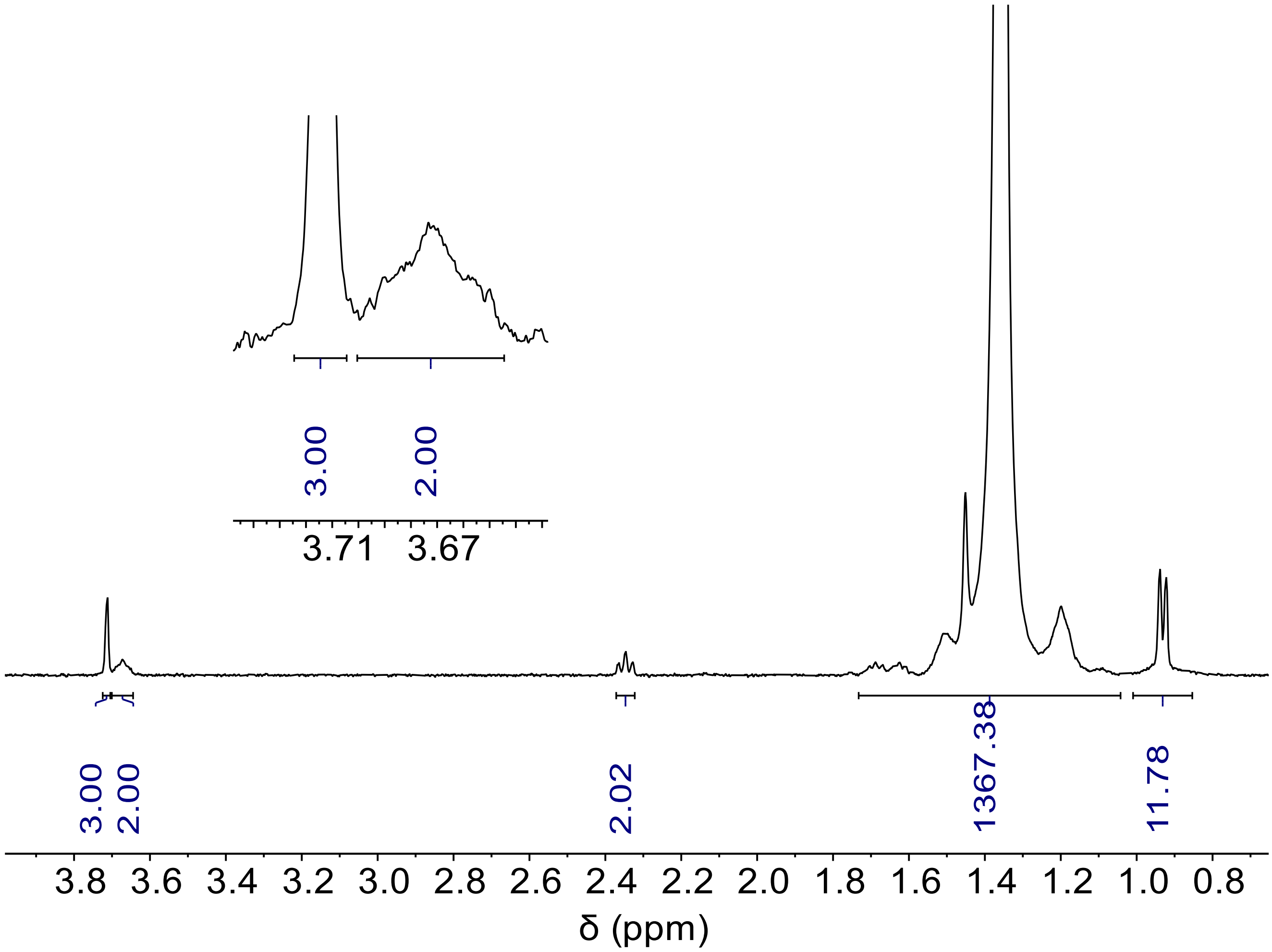

Figure S18. ^{1}H NMR spectrum of **MM$_{tPE\text{-}10K}$** in $C_2D_2Cl_4$ at 110 °C.

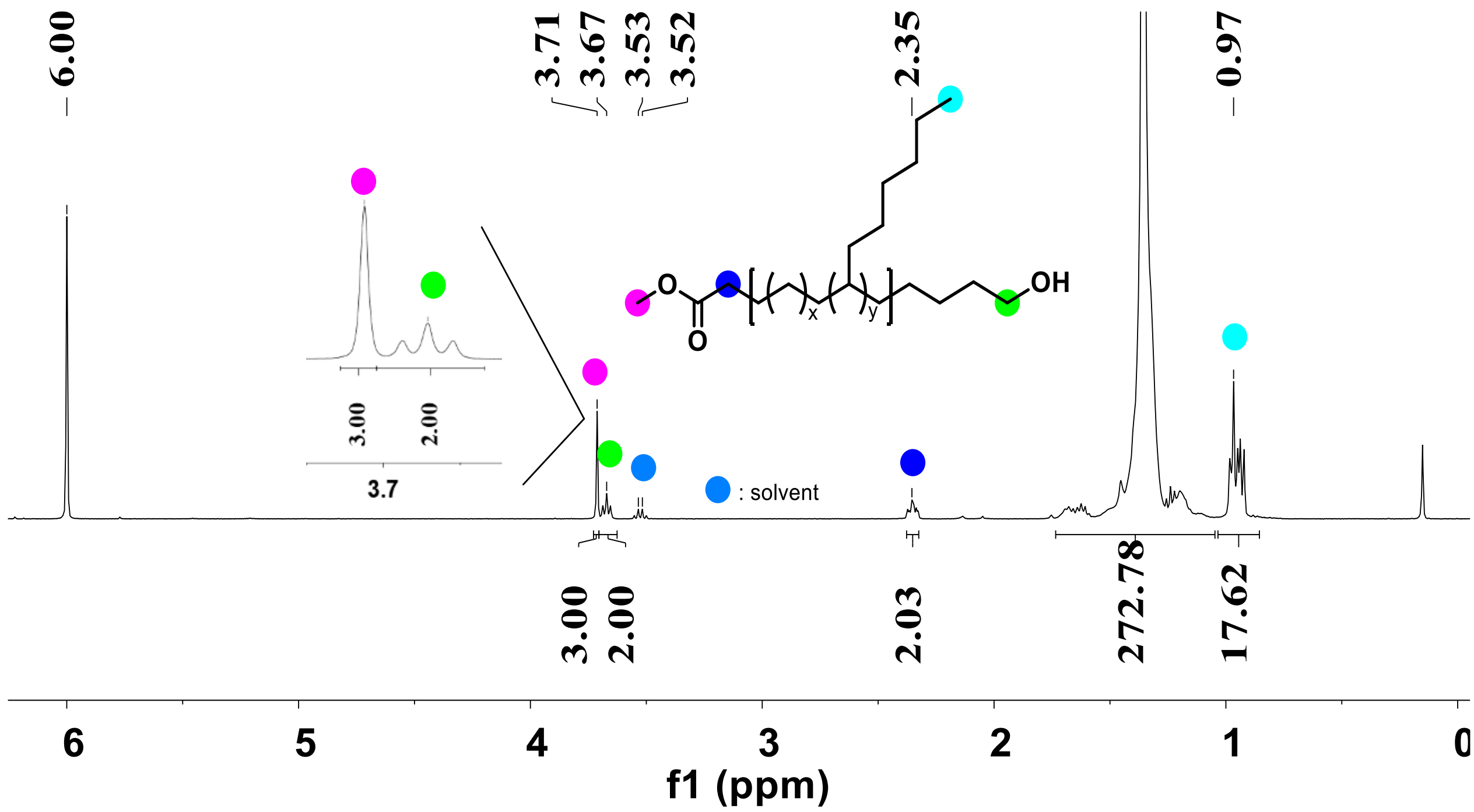

Figure S19. ^{1}H NMR spectrum of **MM$_{tPOE\text{-}2K(6.7\%)}$** in $C_2D_2Cl_4$ at 110 °C.

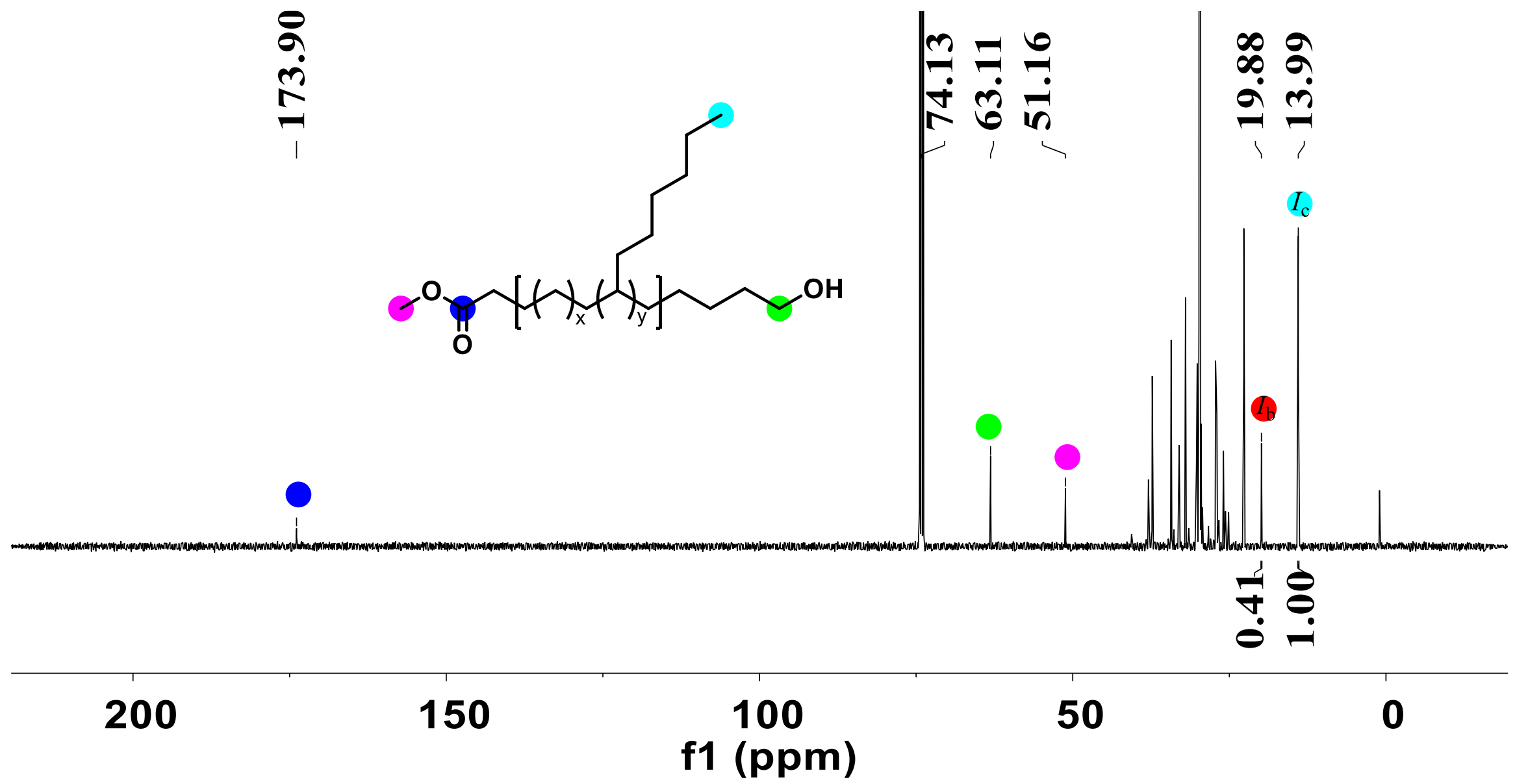

Figure S20. ^{13}C NMR spectrum of $\mathbf{MM}_{t\mathbf{POE\text{-}2K(6.7\%)}}$ in $C_2D_2Cl_4$ at 110 °C.

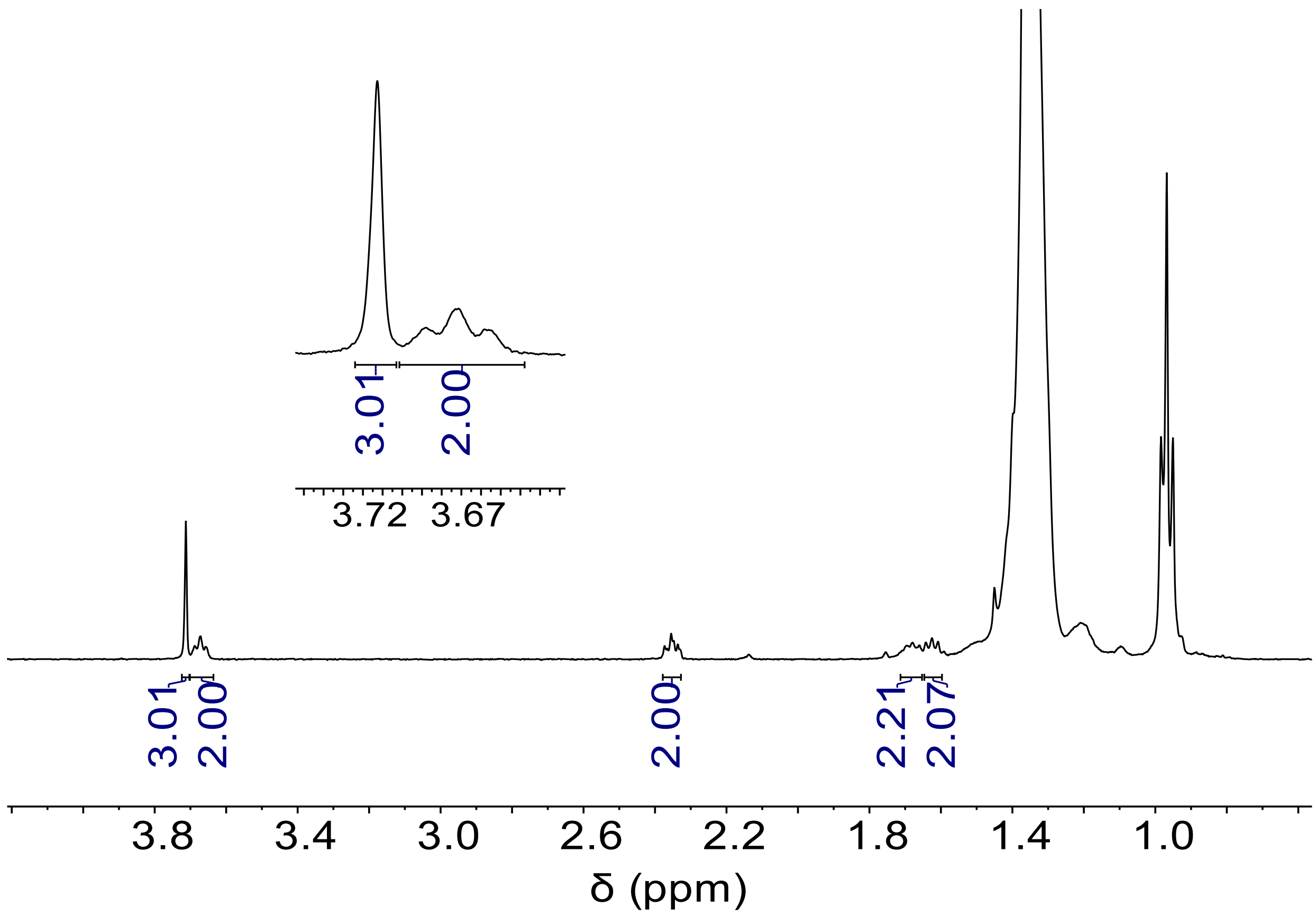

Figure S21. ^{1}H NMR spectrum of $\mathbf{MM}_{t\mathbf{POE\text{-}2.5K(17.4\%)}}$ in $C_2D_2Cl_4$ at 110 °C.

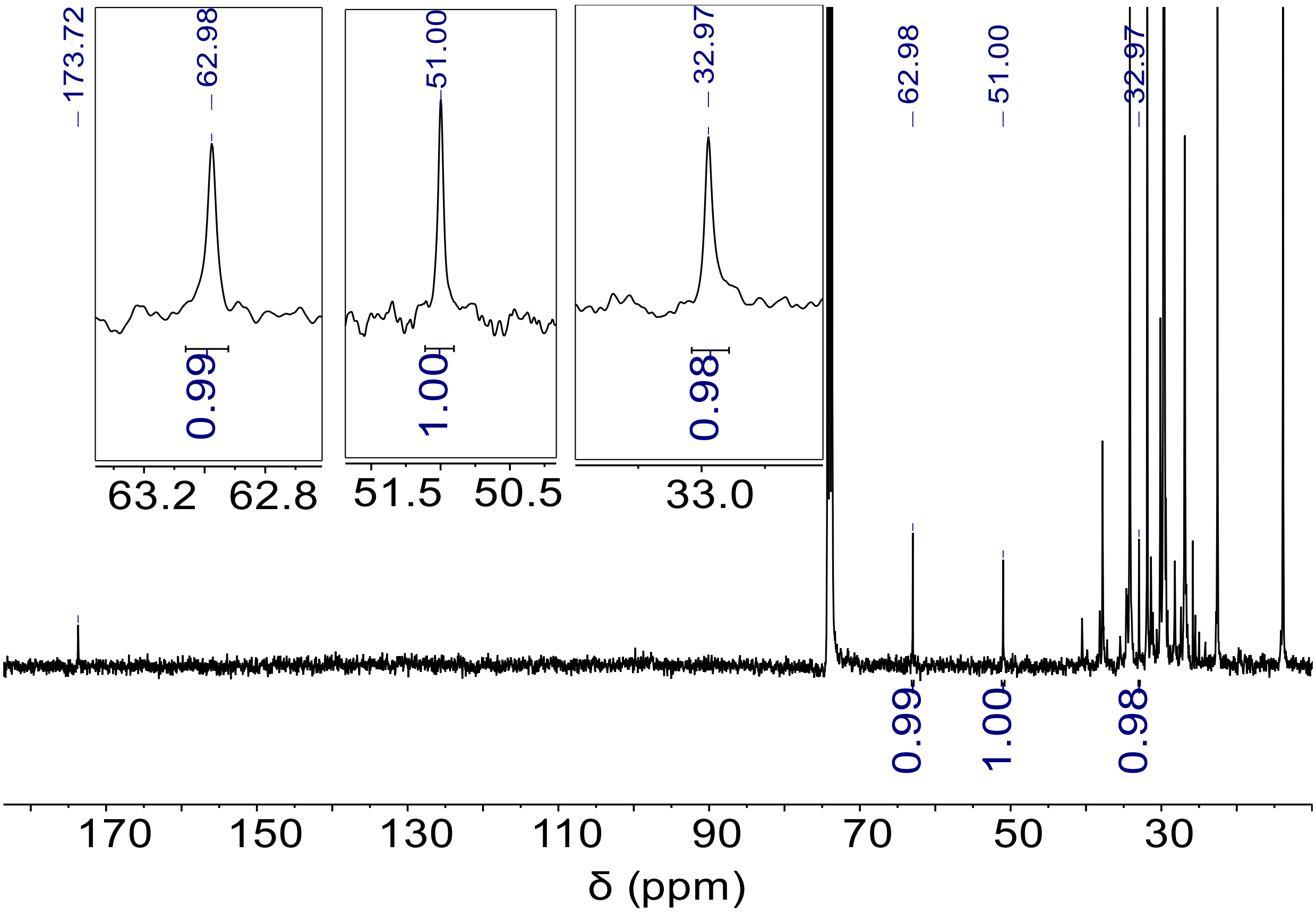

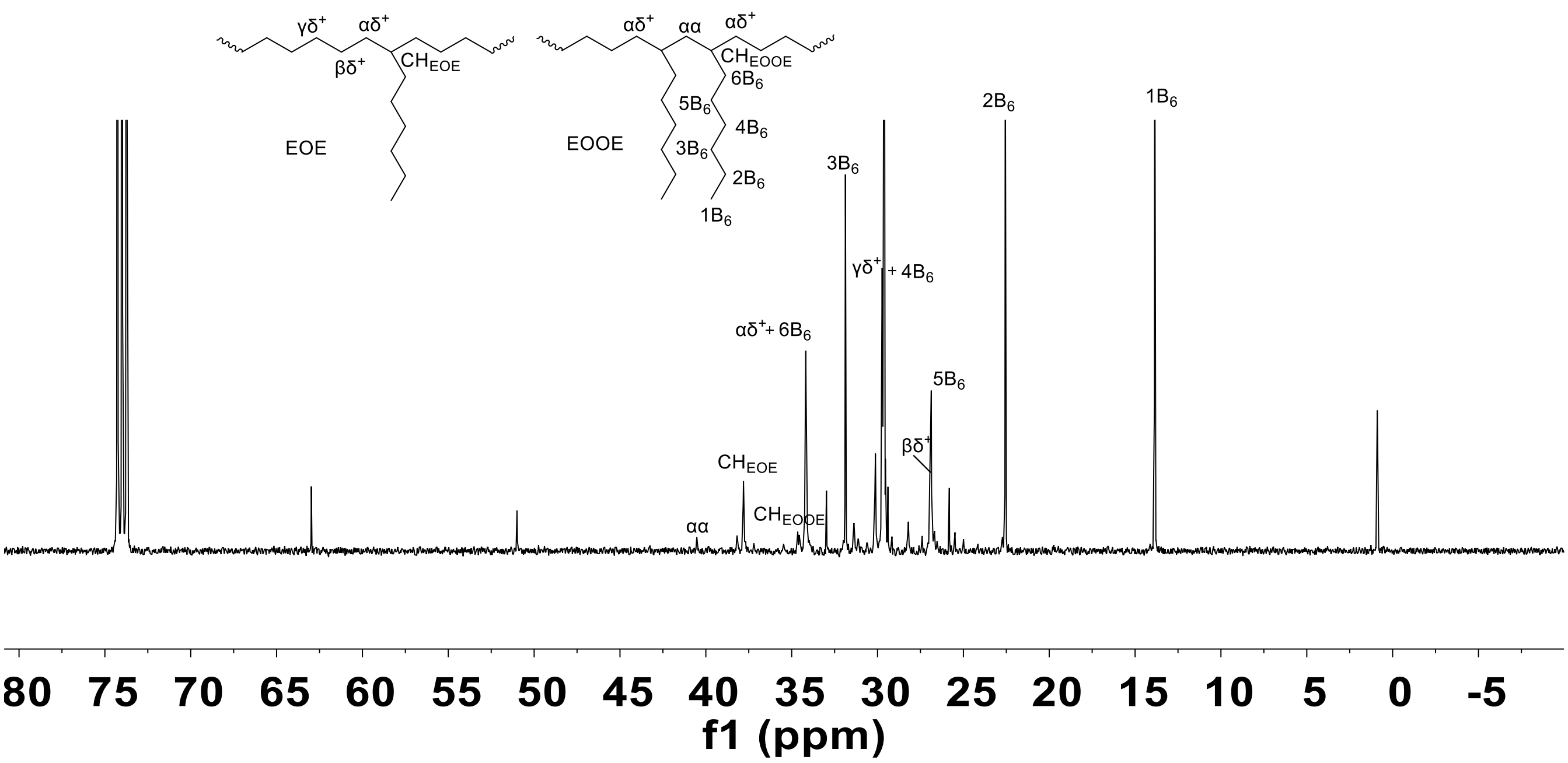

Figure S22. ^{13}C NMR spectrum of **MM$_{tPOE\text{-}2.5K(17.4\%)}$** in $C_2D_2Cl_4$ at 110 °C. (Assigned according to E-O copolymer, O: octene, E: ethylene)

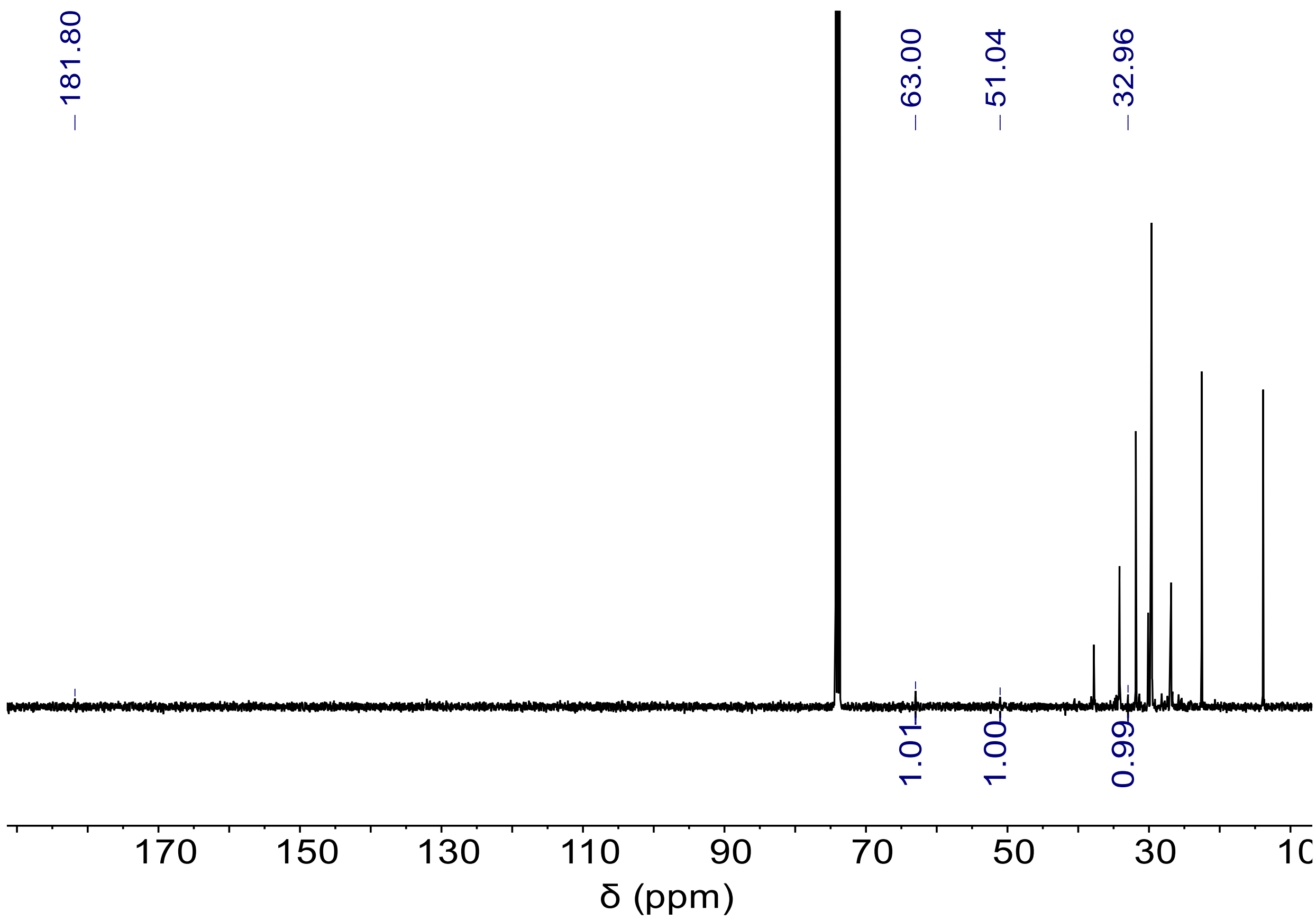

Figure S23. ^{13}C NMR spectrum of $\mathbf{MM}_{\mathbf{\mathit{t}POE\text{-}4K(24.1\%)}}$ in $C_2D_2Cl_4$ at 110 °C.

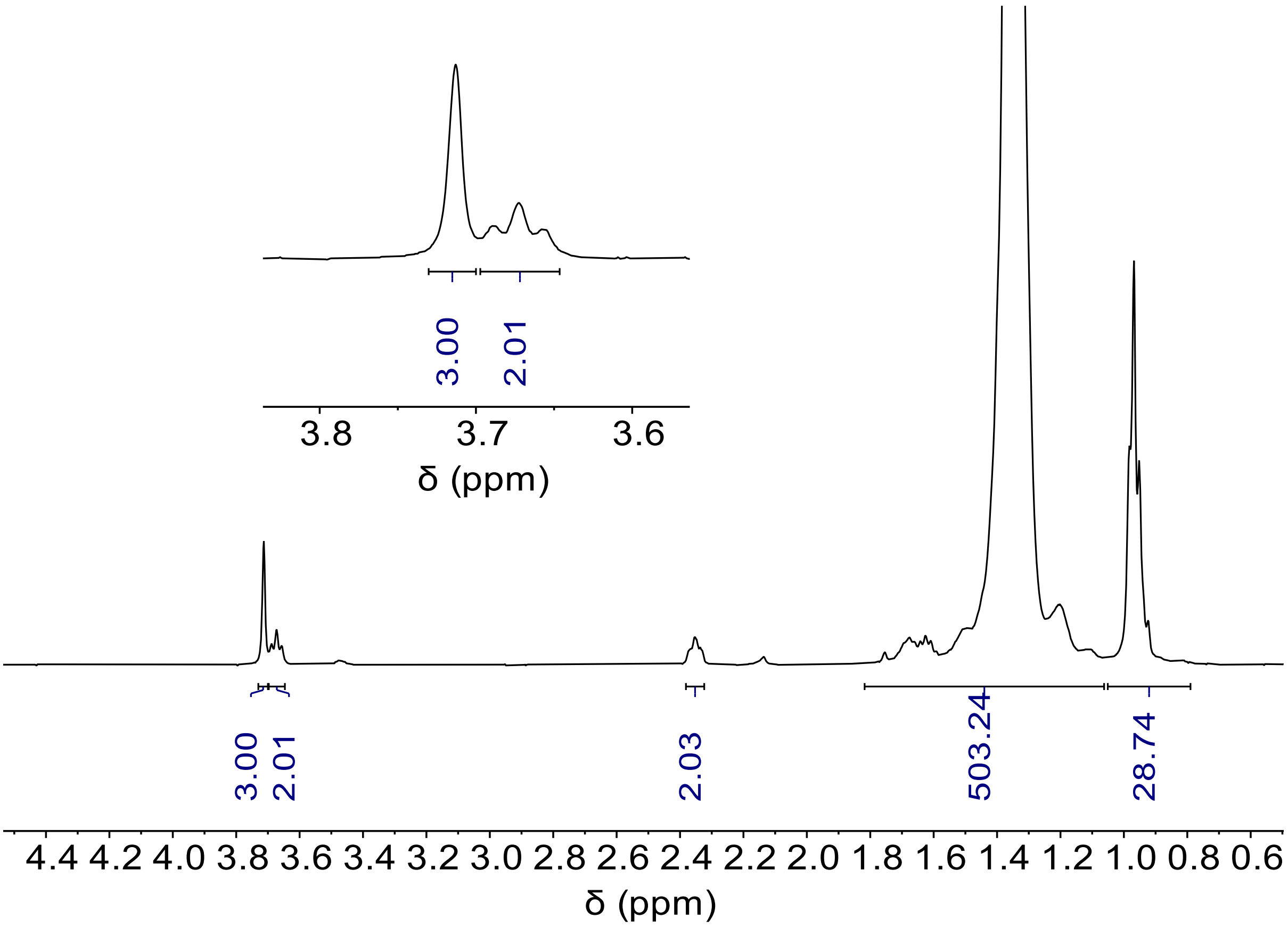

Figure S24. ^{1}H NMR spectrum of $\mathbf{MM}_{\mathbf{\mathit{t}OBC\text{-}4K(8.9\%)}}$ in $C_2D_2Cl_4$ at 110 °C.

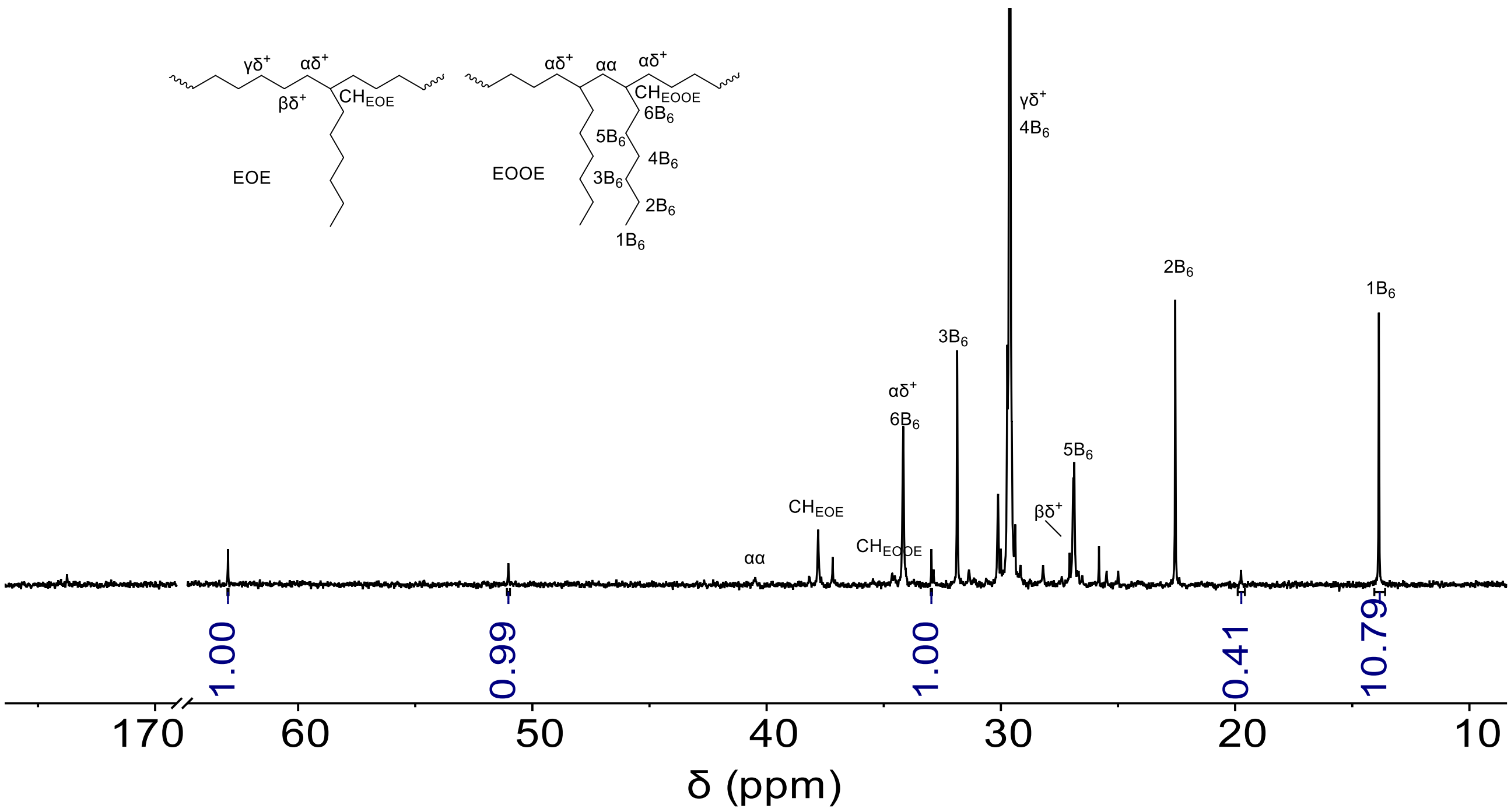

Figure S25. ^{13}C NMR spectrum of $\mathbf{MM}_{\boldsymbol{t}\mathbf{OBC\text{-}4K(8.9\%)}}$ in $C_2D_2Cl_4$ at 110 °C.

4.5 NMR spectra of *d*PO

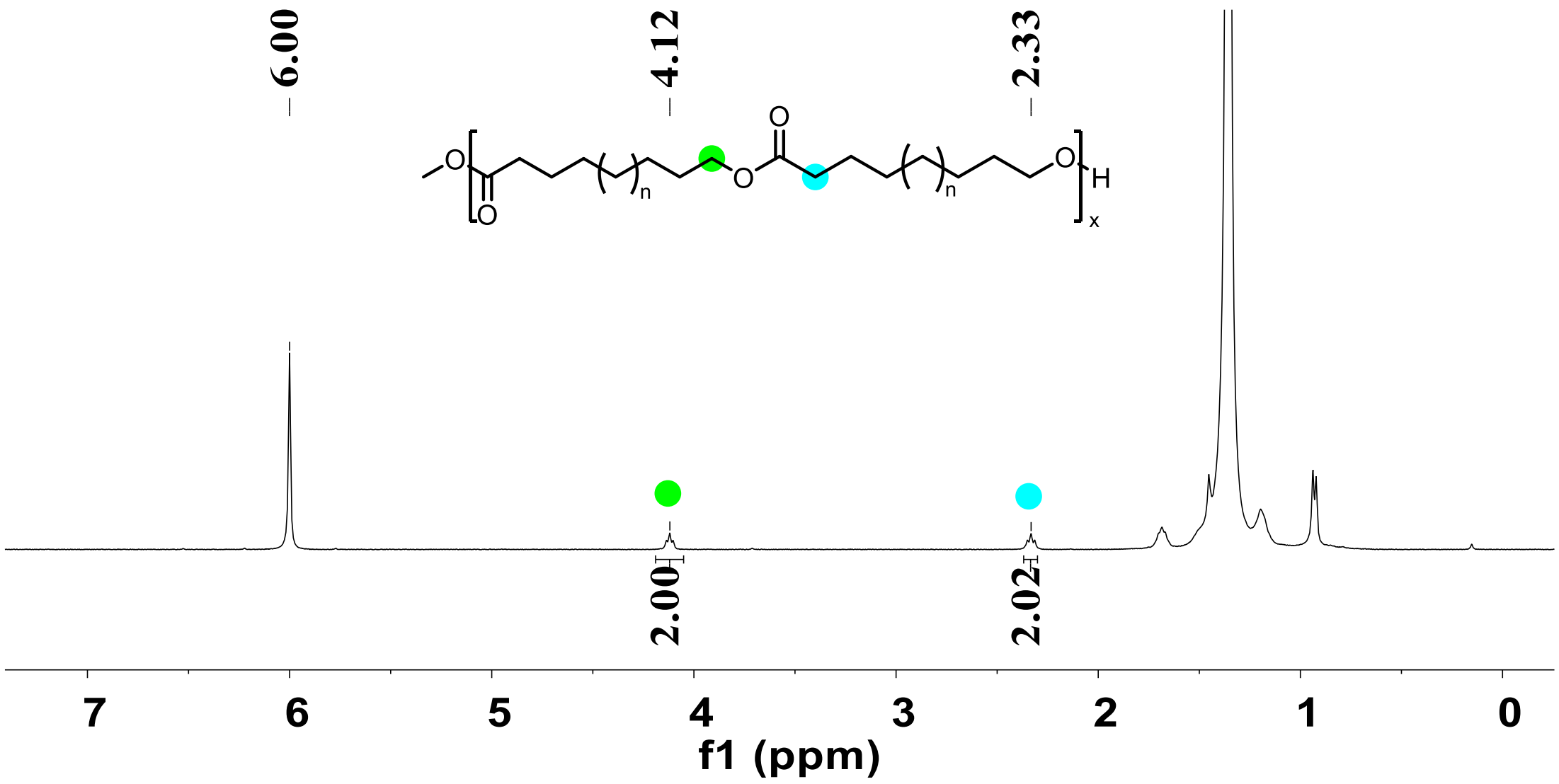

Figure S26. ^{1}H NMR spectrum of ***d*PE-0.9%** in $C_2D_2Cl_4$ at 110 °C.

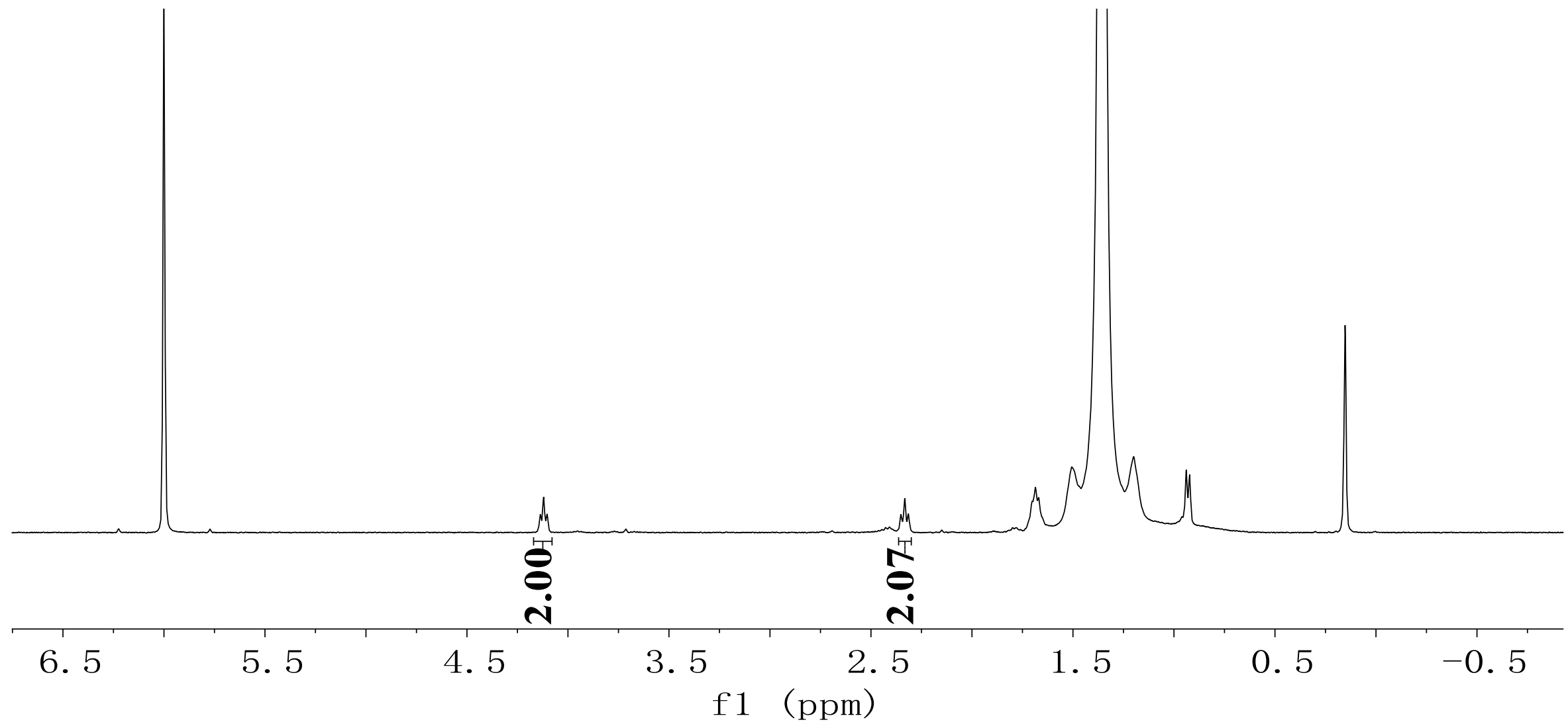

Figure S27. ^{1}H NMR spectrum of ***d*PE-0.4%** in $C_2D_2Cl_4$ at 110 °C.

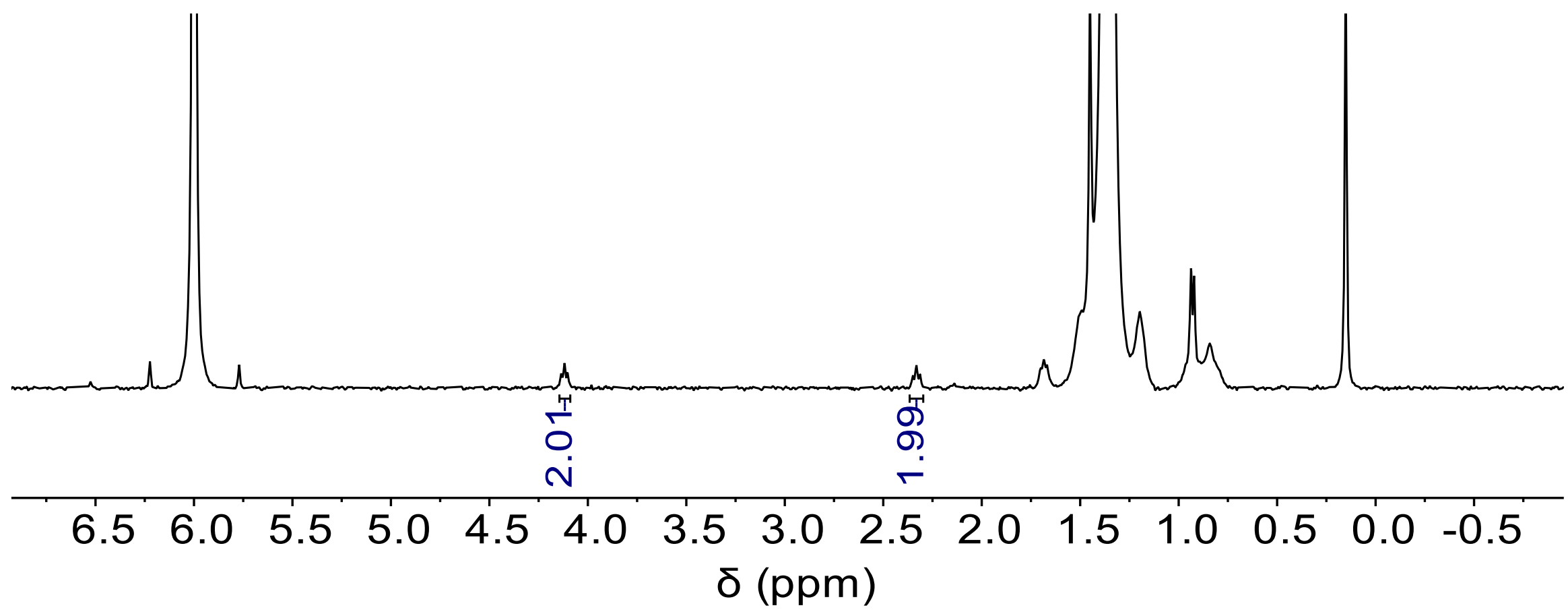

Figure S28. ^{1}H NMR spectrum of ***d*PE-0.3%** in $C_2D_2Cl_4$ at 110 °C.

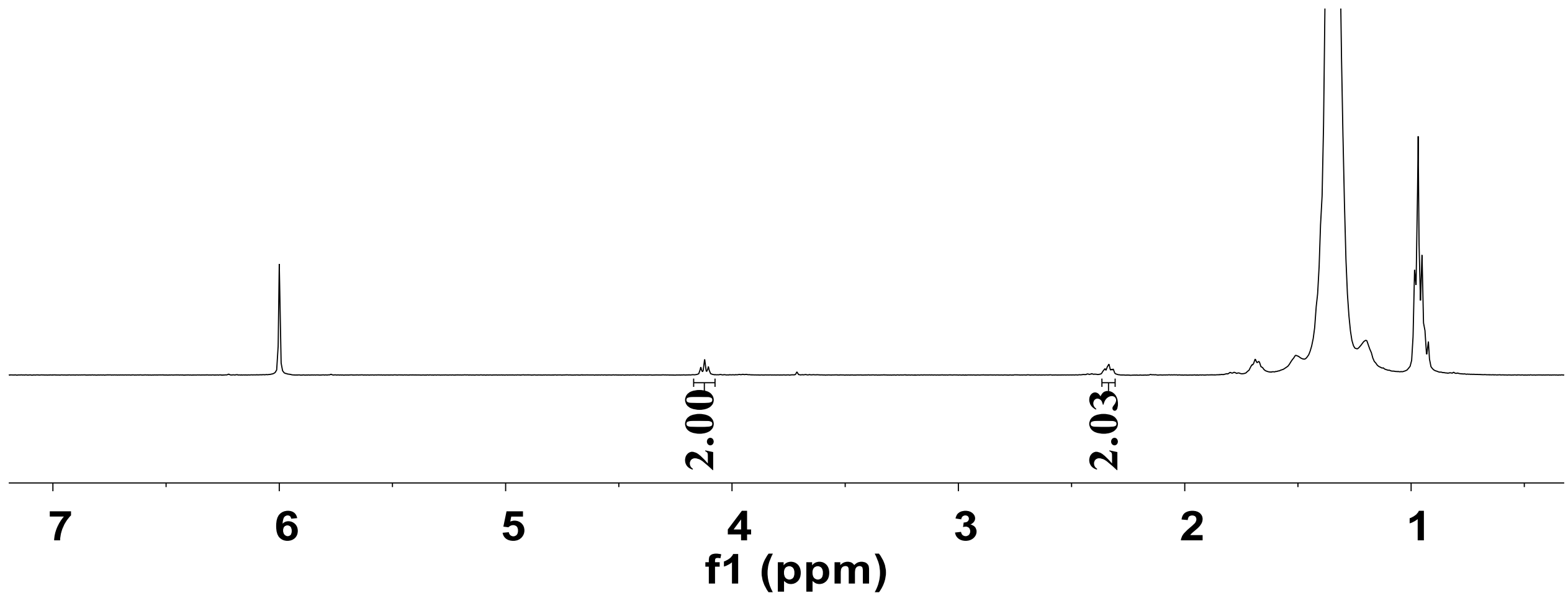

Figure S29. ^{1}H NMR spectrum of ***d*(*r*-OBC-0.6%)** in $C_2D_2Cl_4$ at 110 °C.

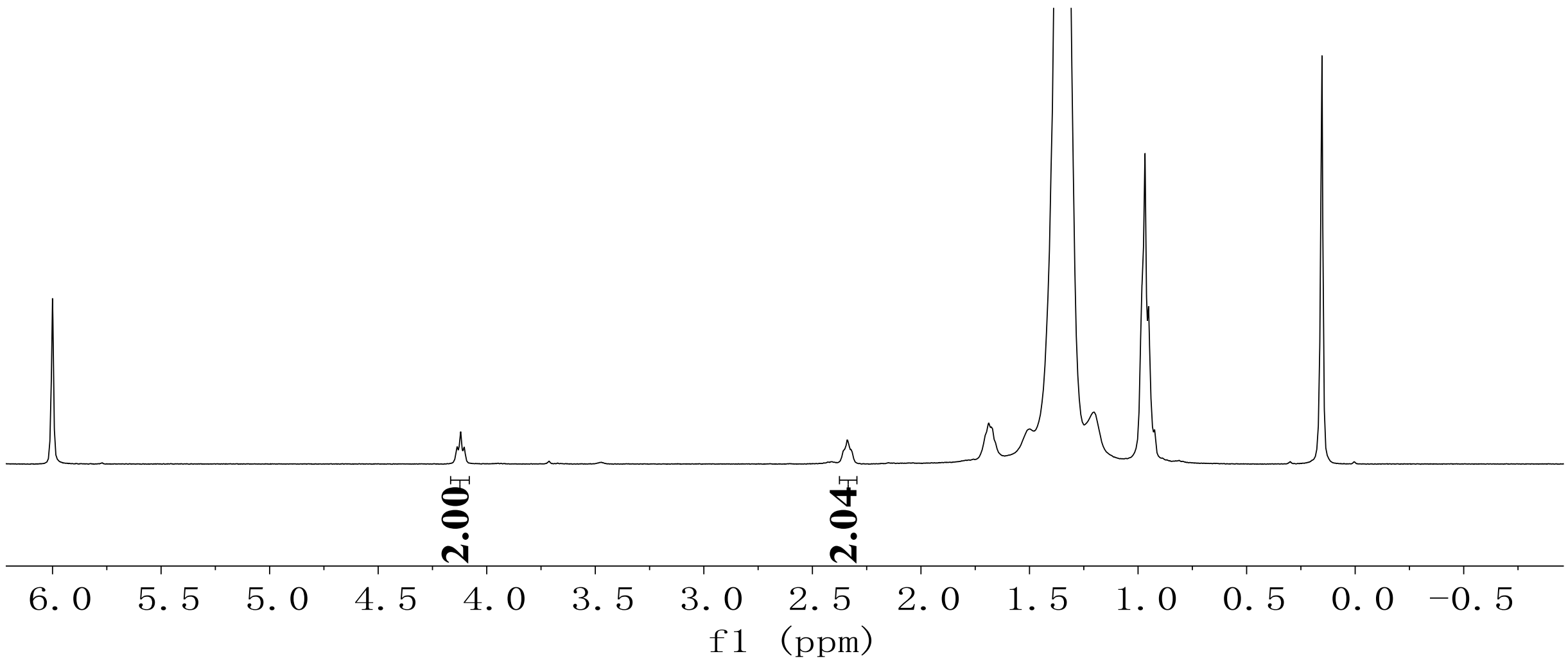

Figure S30. ^{1}H NMR spectrum of ***d*(*a*-OBC-0.9%)** in $C_2D_2Cl_4$ at 110 °C.

4.6 NMR spectra of the recycled macromonomers

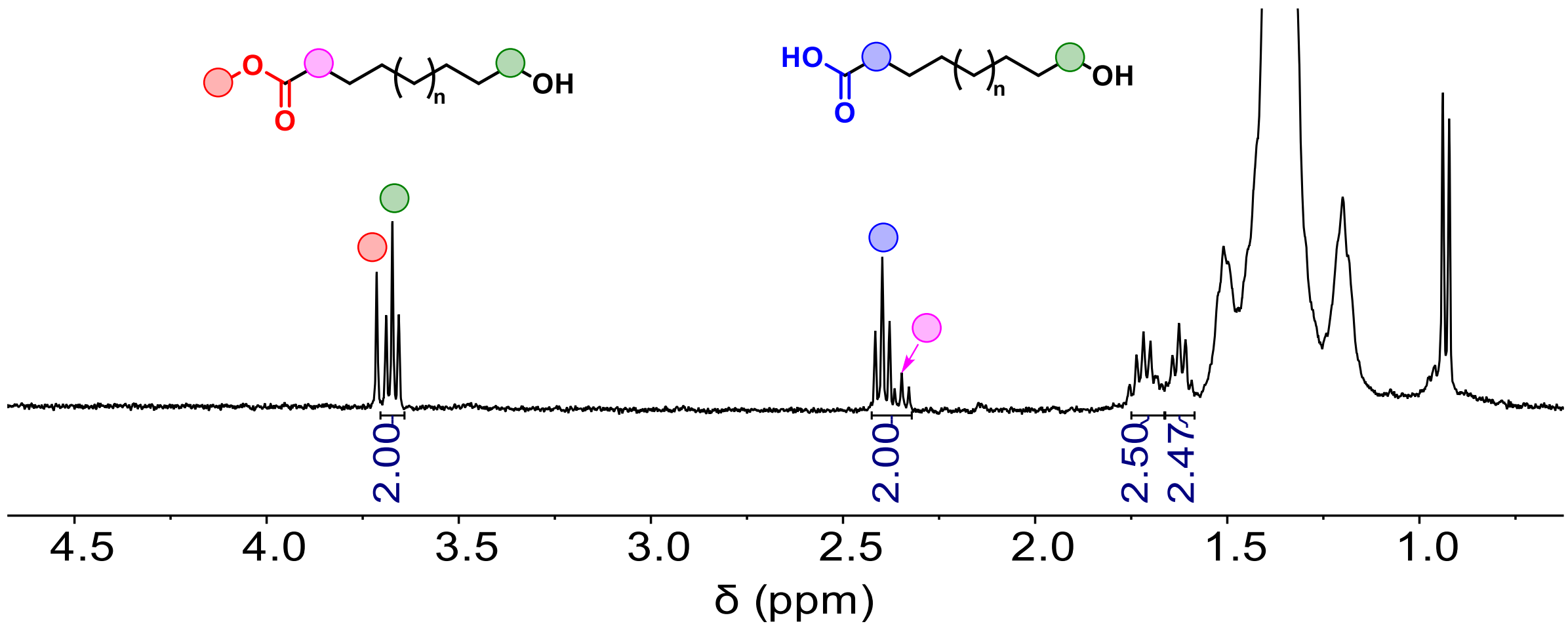

Figure S31. ^{1}H NMR spectrum of **MM$_{tPE\text{-}7K}$-1** in $C_2D_2Cl_4$ at 110 °C.

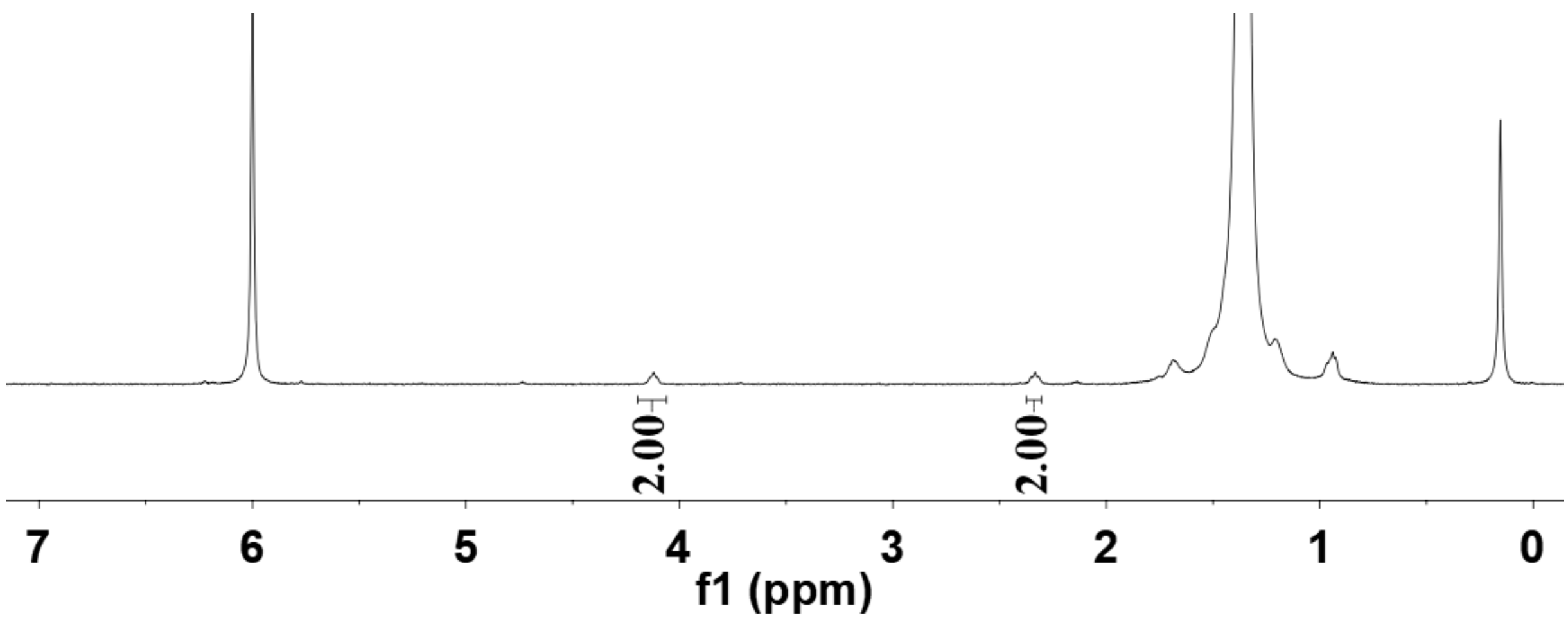

Figure S32. ^{1}H NMR spectrum of ***d*PE-0.4%-RP-1** in $C_2D_2Cl_4$ at 110 °C

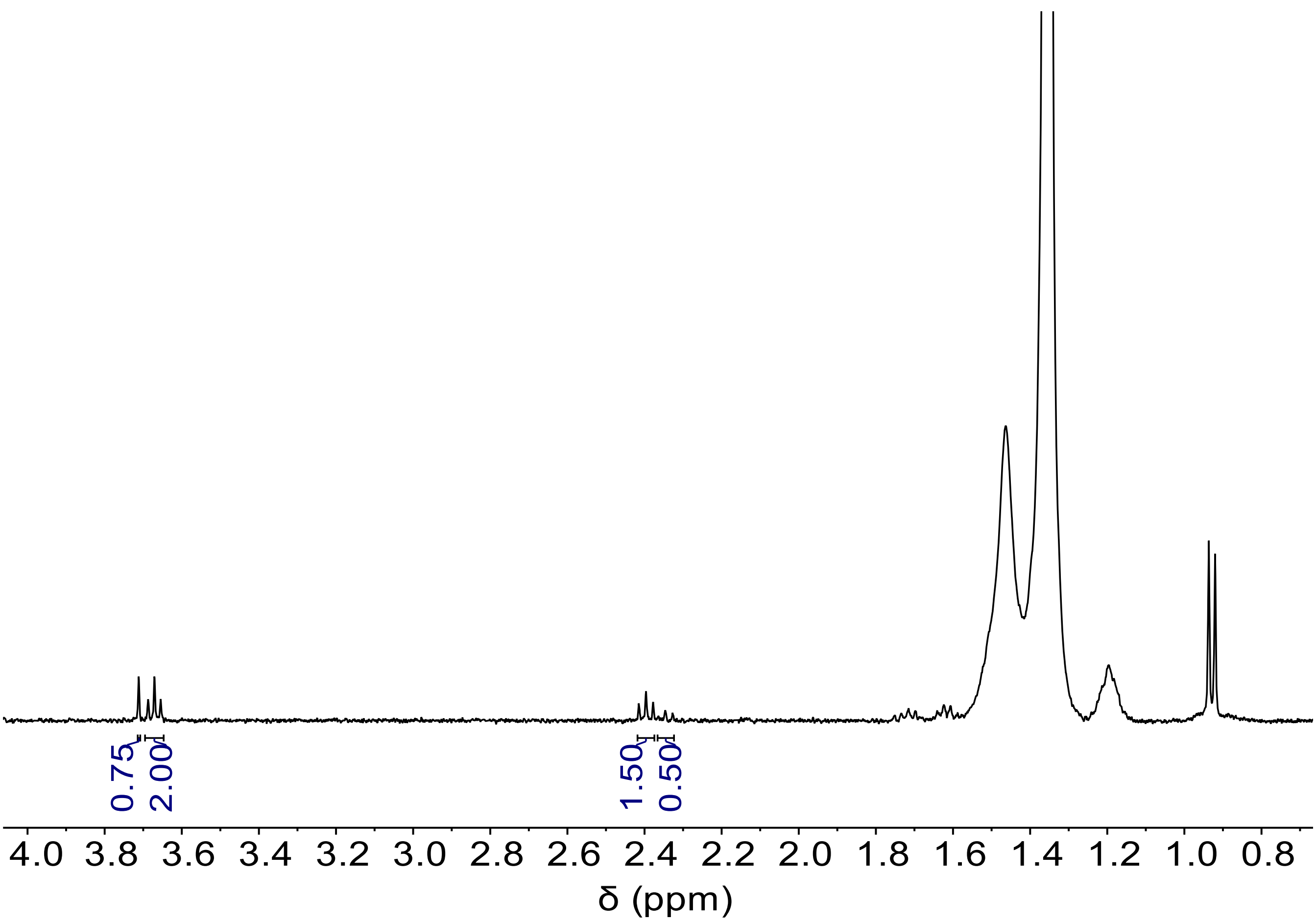

Figure S33. ^{1}H NMR spectrum of **MM$_{t\mathbf{PE\text{-}10K}}$-1** in $C_2D_2Cl_4$ at 110 °C.

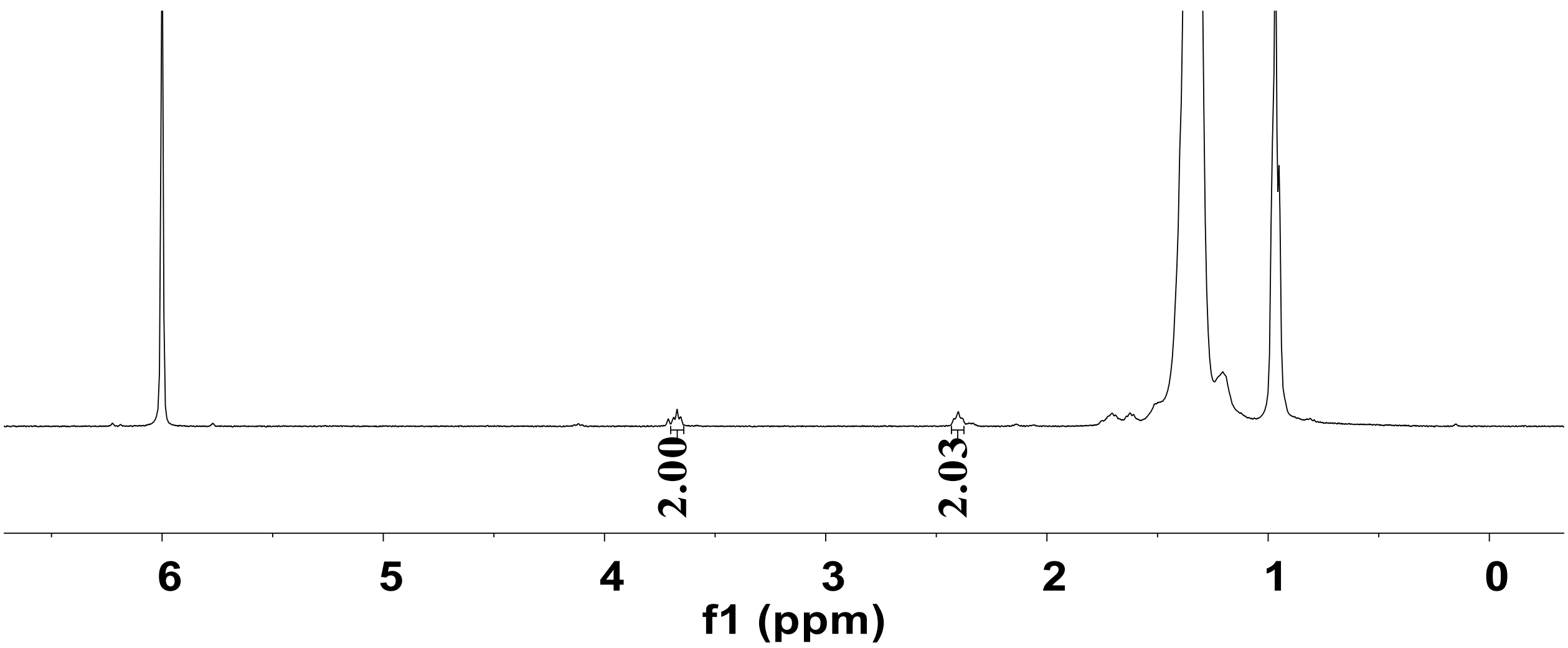

Figure S34. ^{1}H NMR spectrum of **MM$_{t\mathbf{POE\text{-}4K(24.1\%)}}$-1** in $C_2D_2Cl_4$ at 110 °C.

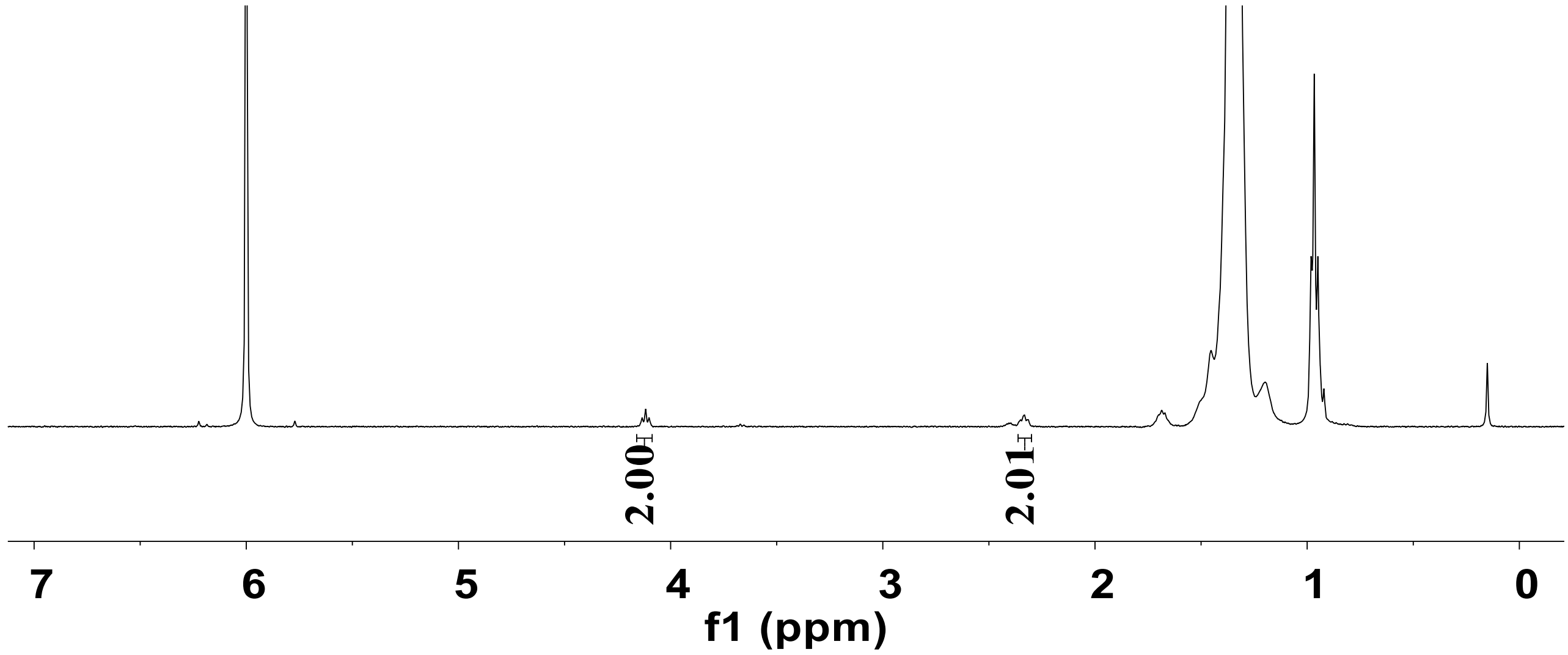

Figure S35. ^{1}H NMR spectrum of ***d*(*r*-OBC-0.6%)-RP-1** in $C_2D_2Cl_4$ at 110 °C.

4.7 DSC traces for MMs and *d*PO

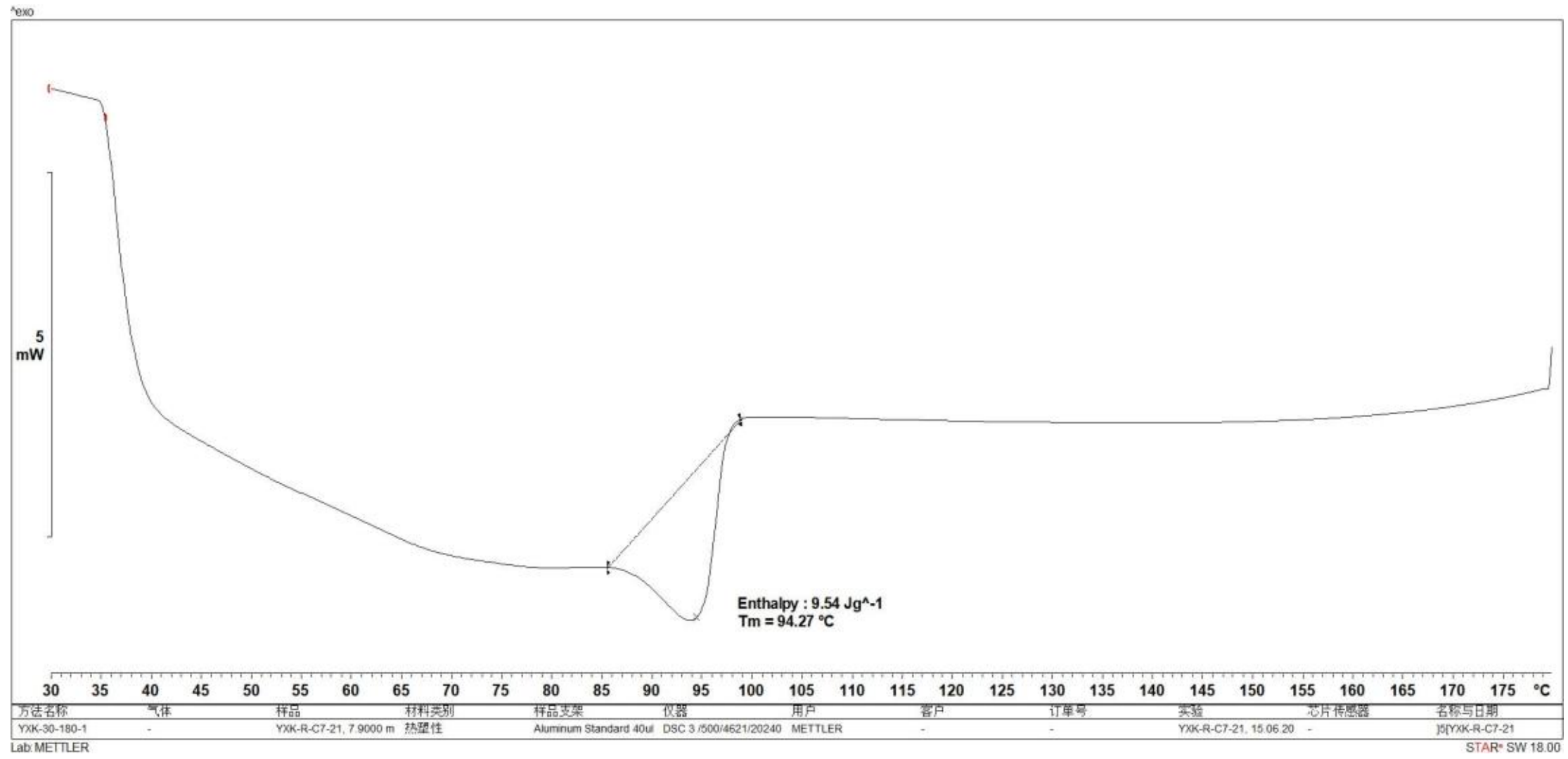

Figure S36. DSC trace of $\mathbf{MM}_{\boldsymbol{t}\mathbf{POE\text{-}2K(6.7\%)}}$.

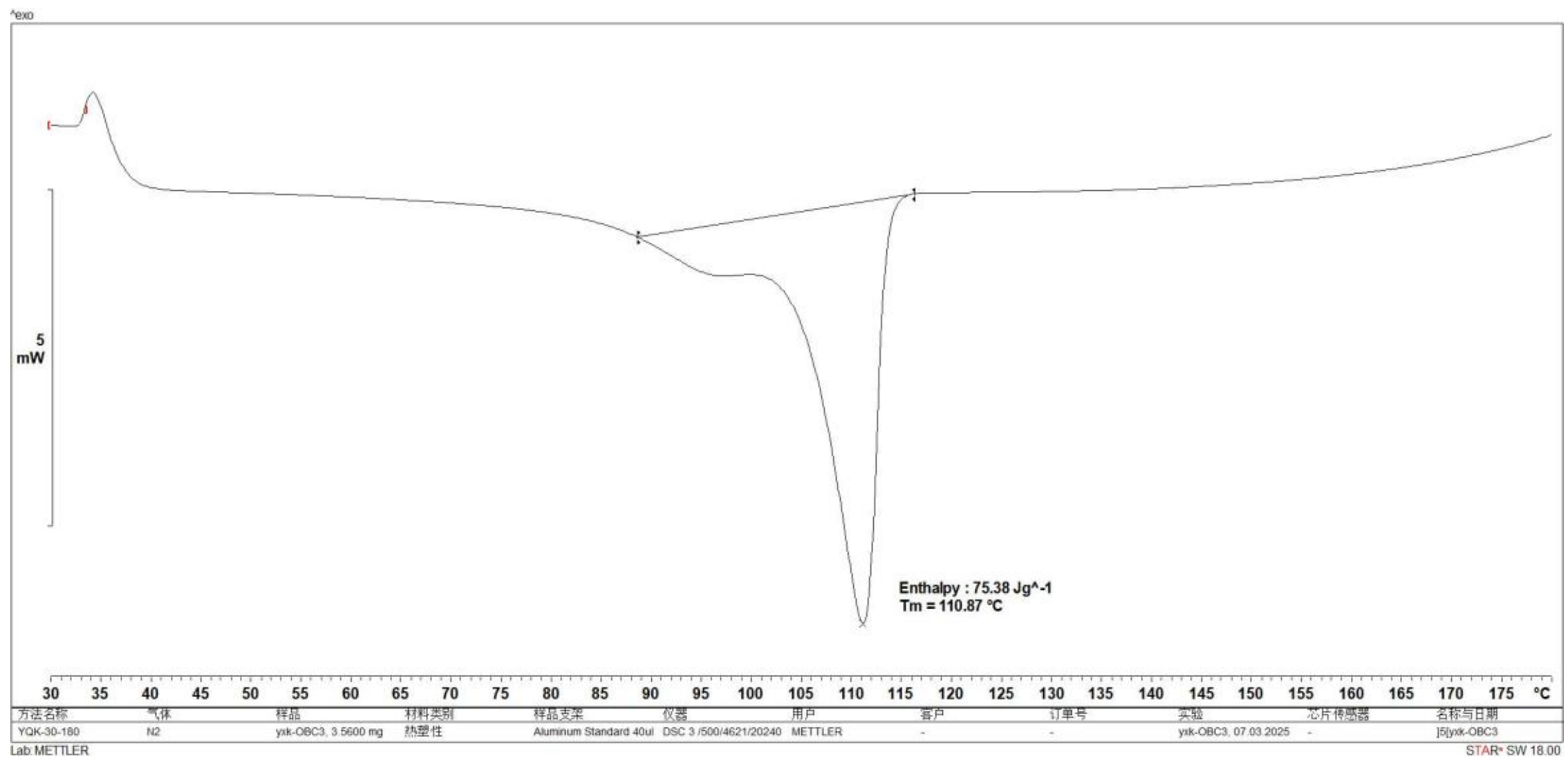

Figure S37. DSC trace of $\mathbf{MM}_{\boldsymbol{t}\mathbf{OBC\text{-}4K(8.9\%)}}$.

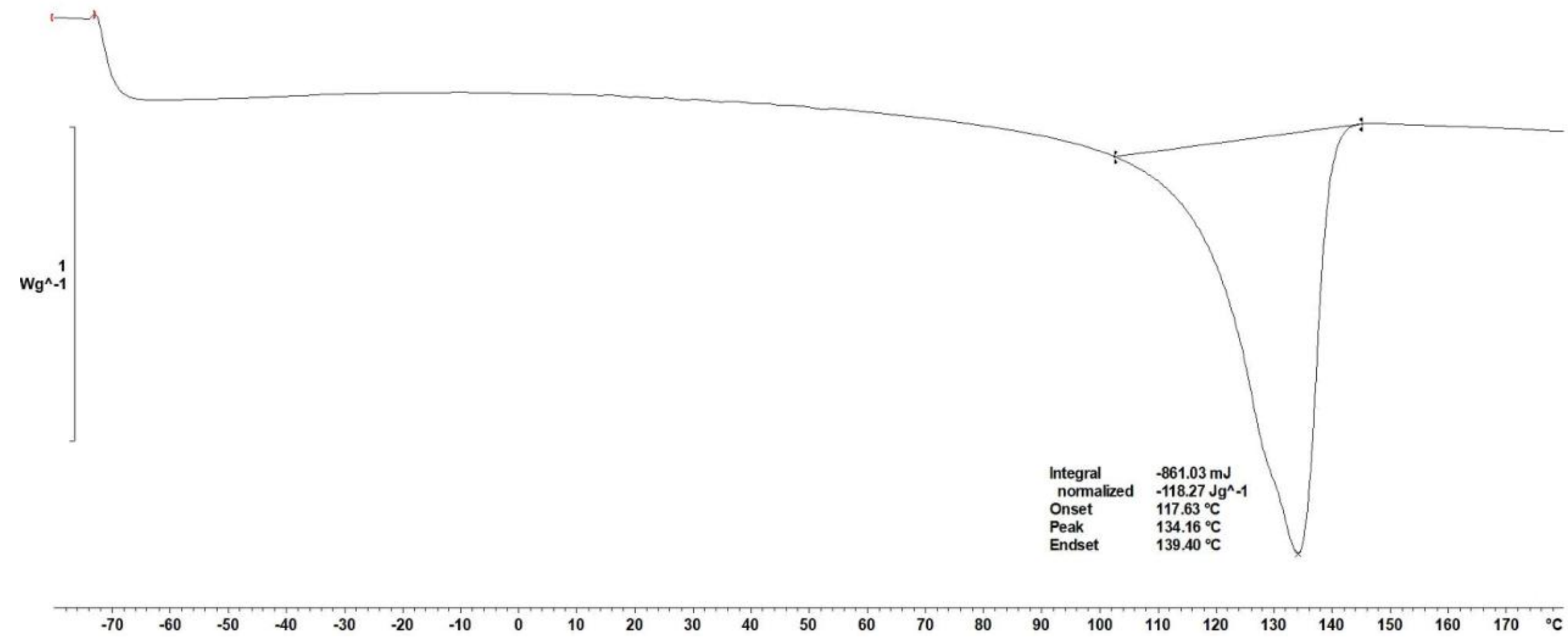

Figure S38. DSC trace of ***d*PE-0.4%**.

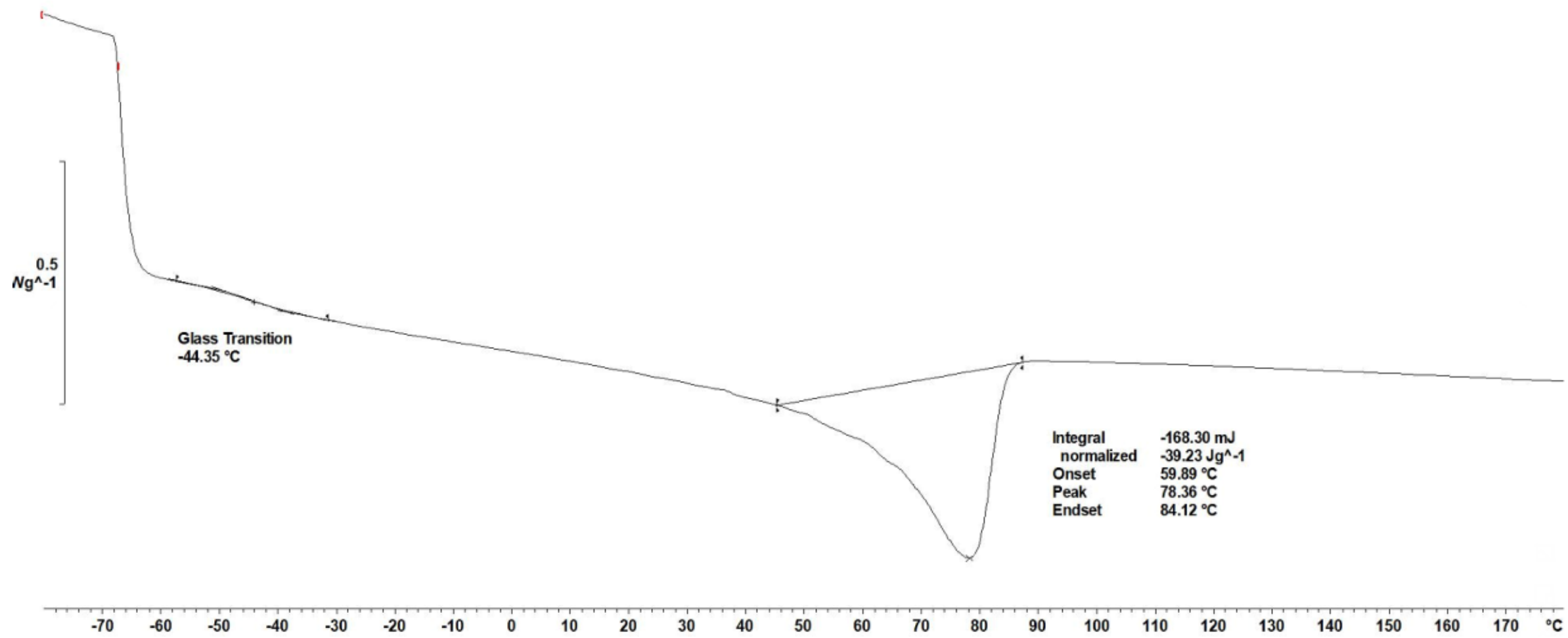

Figure S39. DSC trace of ***d*POE-1.6%.**

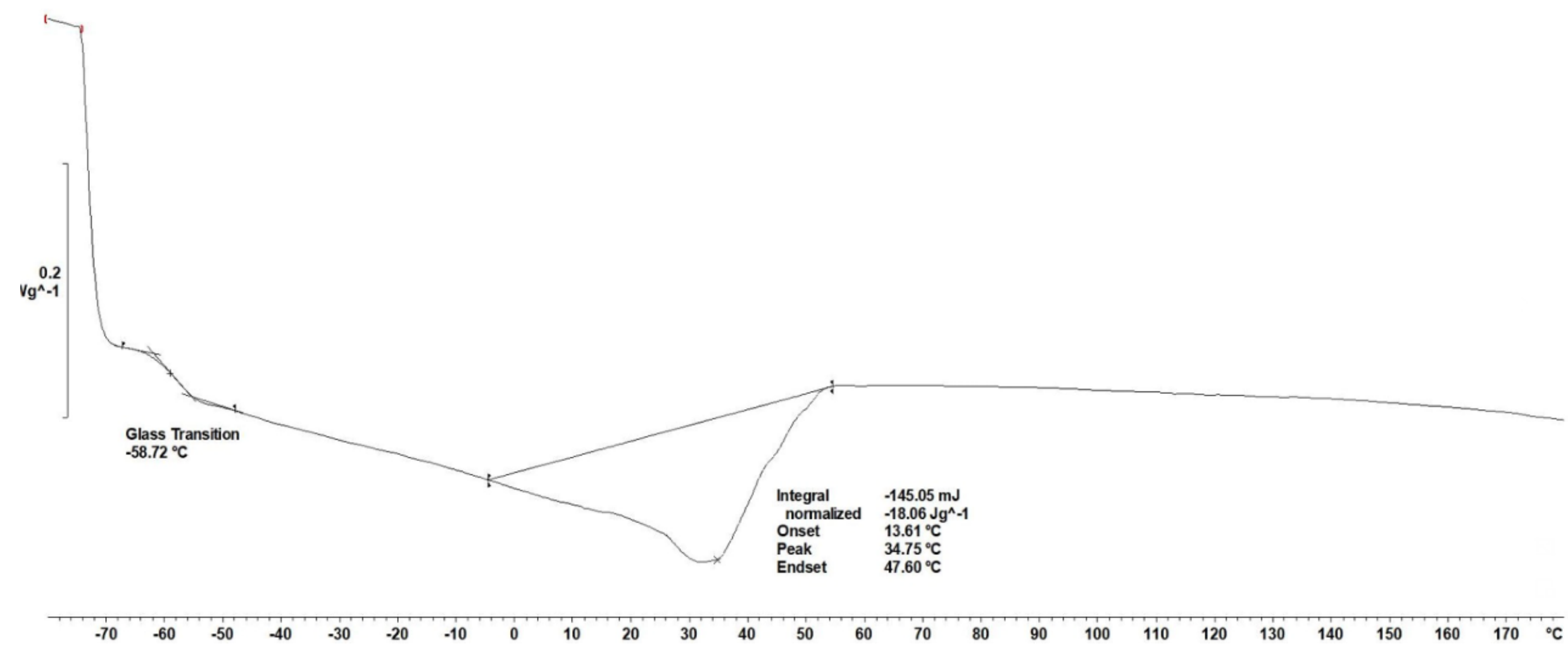

Figure S40. DSC trace of ***d*POE-1.7%.**

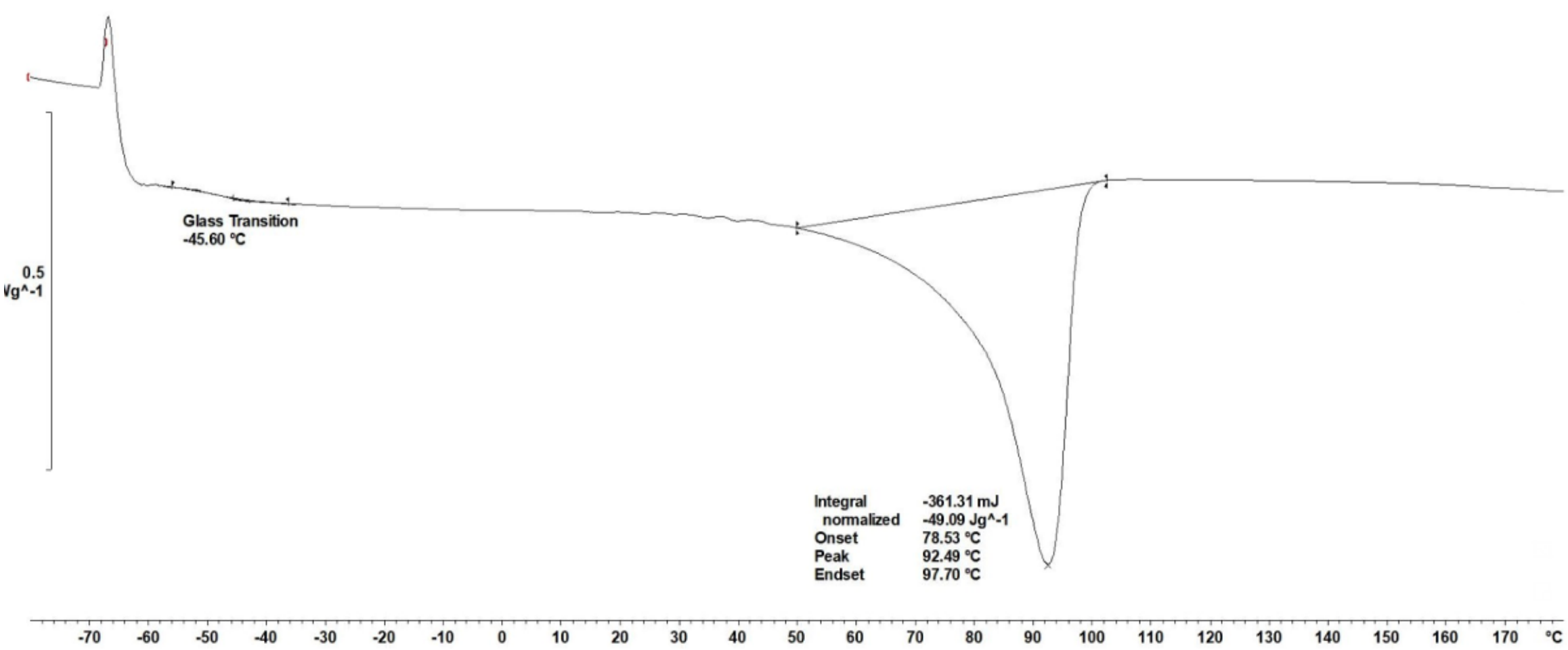

Figure S41. DSC trace of ***d*(*a*-OBC-0.9%).**

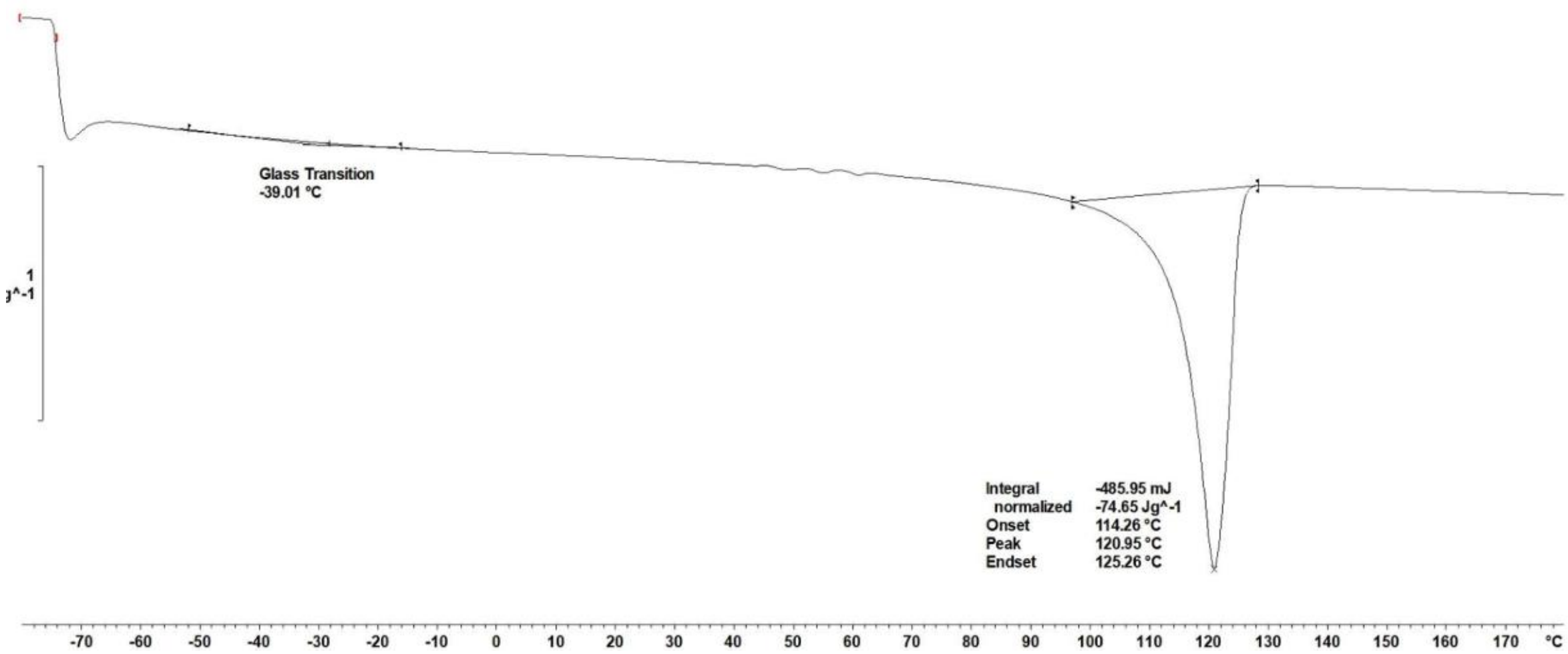

Figure S42. DSC trace of ***d*(*r*-OBC-0.6%).**

4.8 DSC traces for recycled polymers

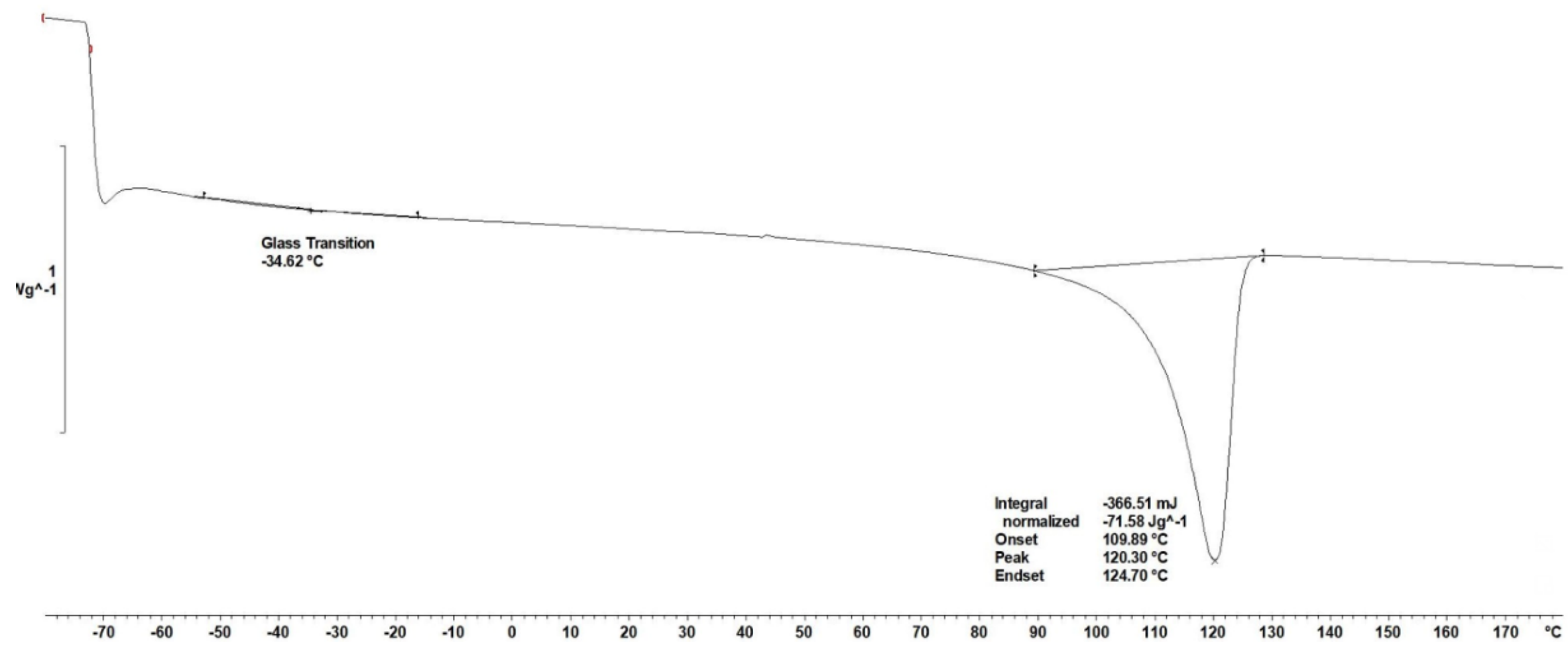

Figure S43. DSC trace of ***d*(*r*-OBC-0.6%)-RP-1.**

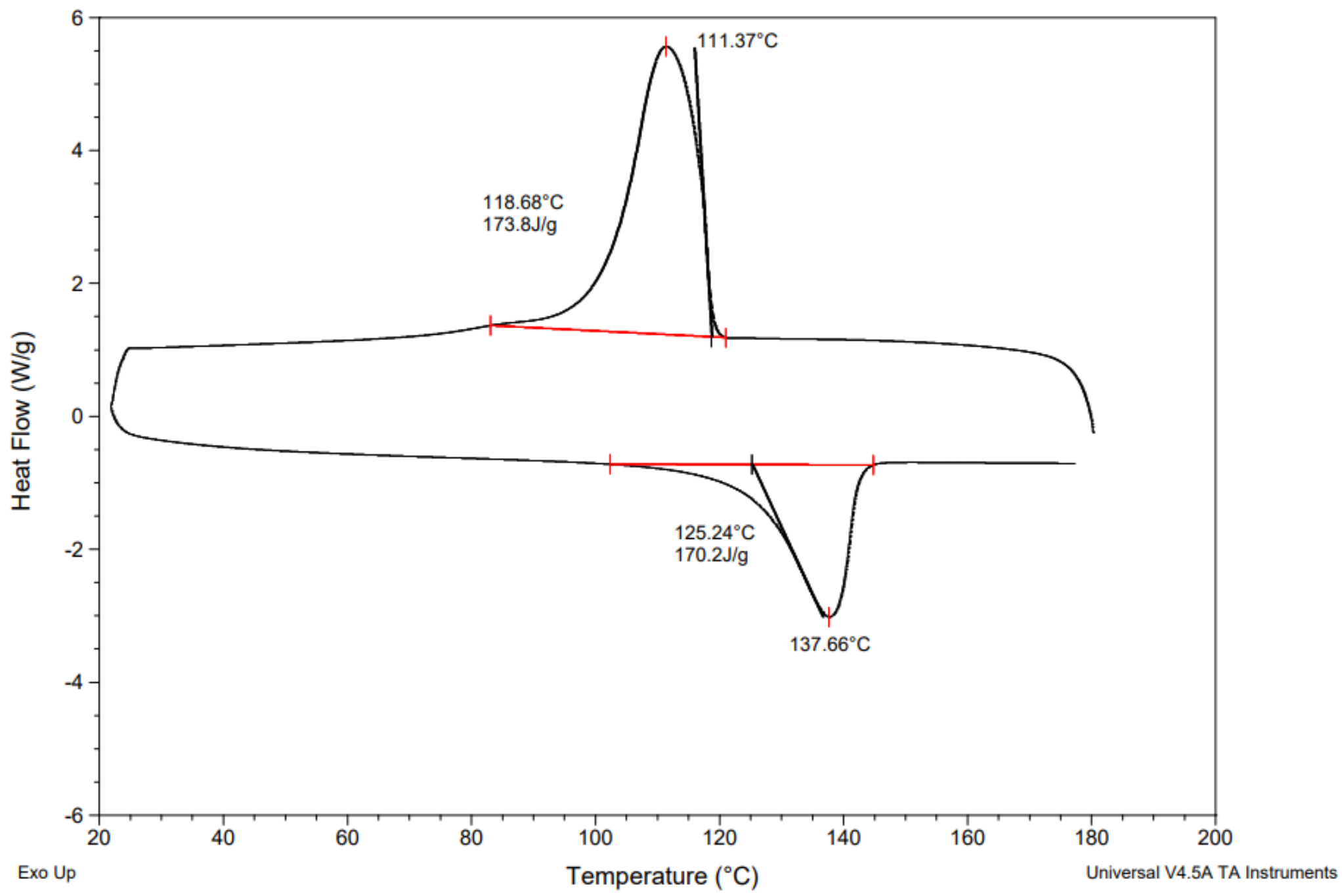

Figure S44. DSC trace of HDPE in Figure 3b.

4.9 Stress-strain curves of *d*PO

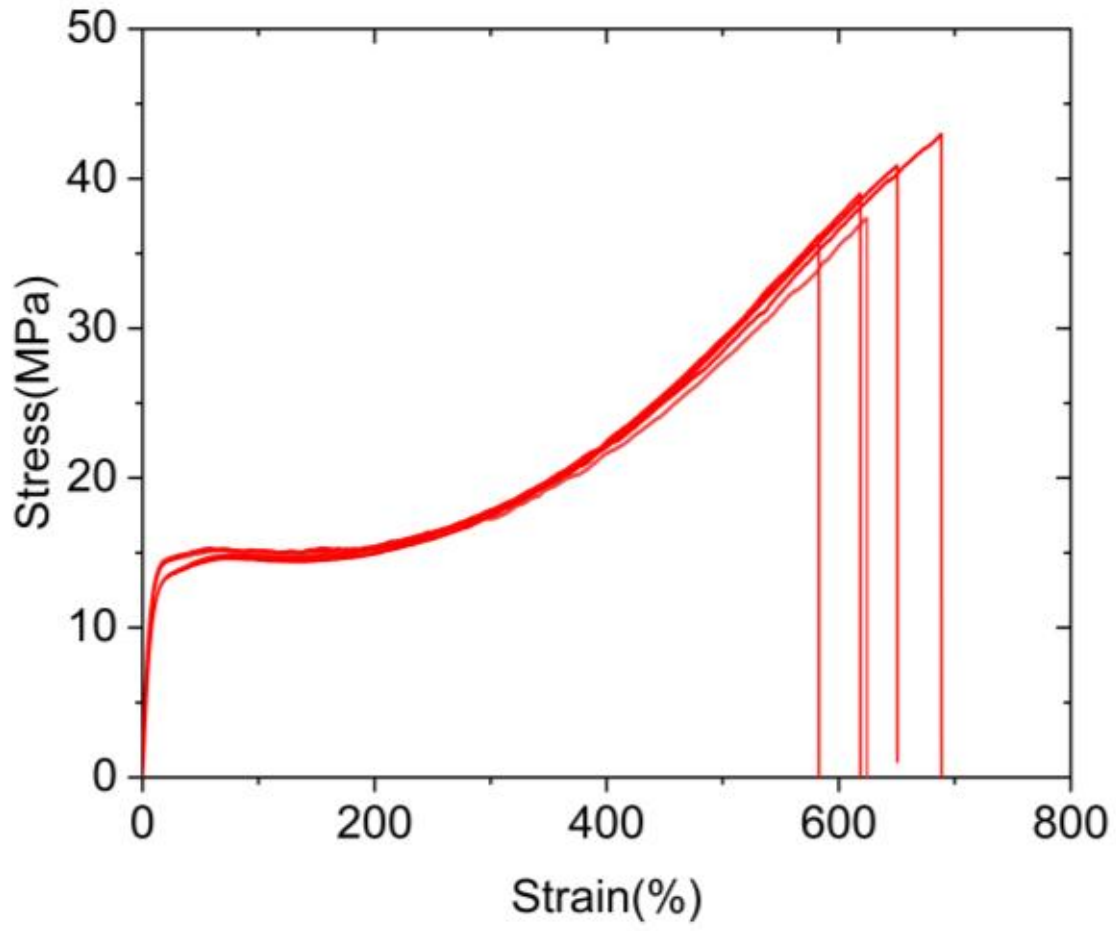

Figure S45. Stress-strain curves of ***d*PE-0.3%**.

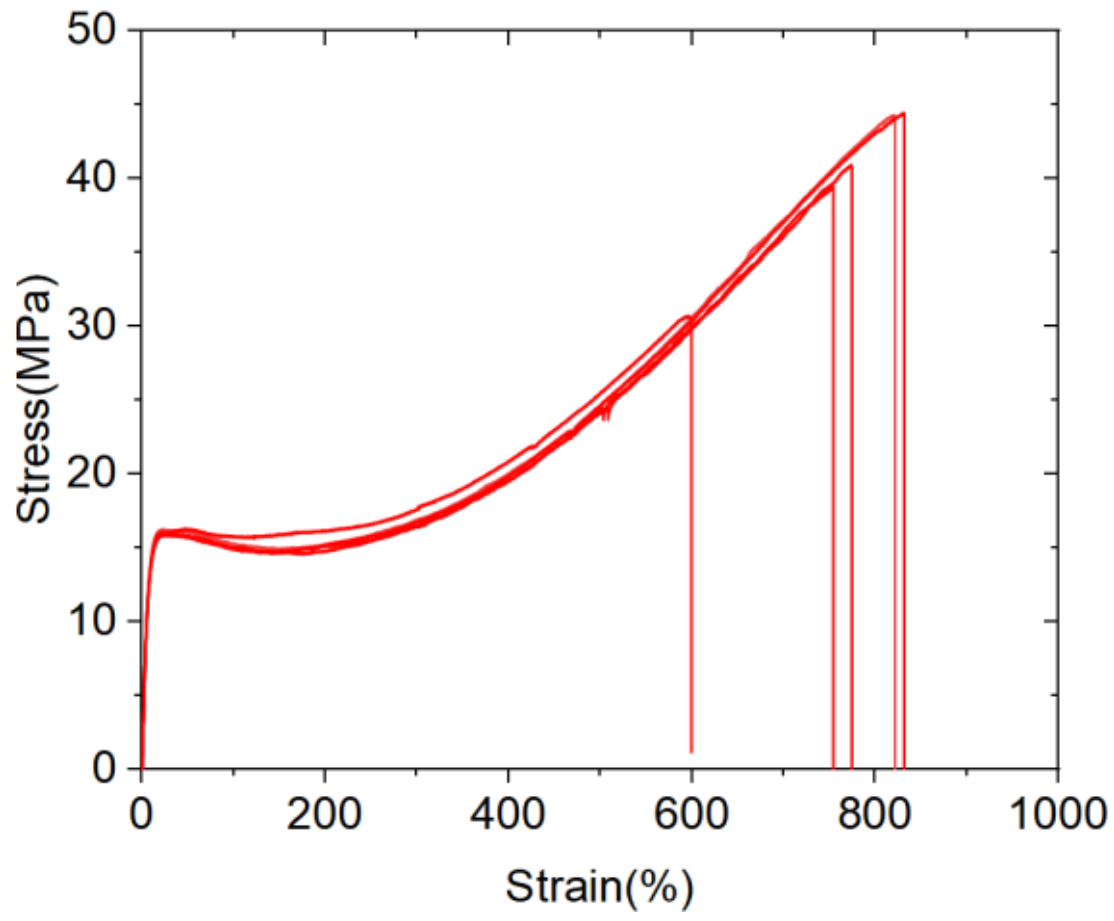

Figure S46. Stress-strain curves of ***d*PE-0.4%**.

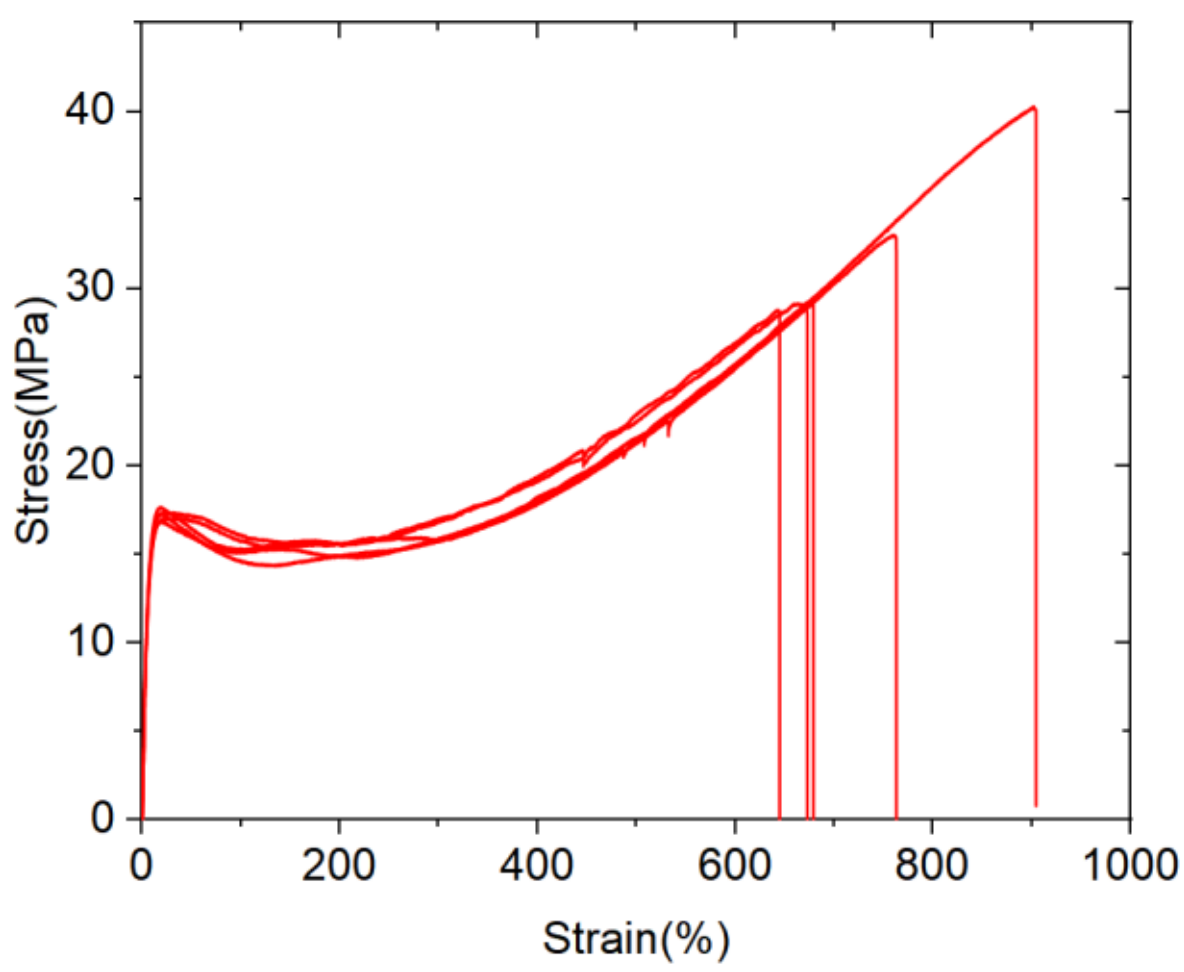

Figure S47. Stress-strain curves of ***d*PE-0.4%-RP-1**.

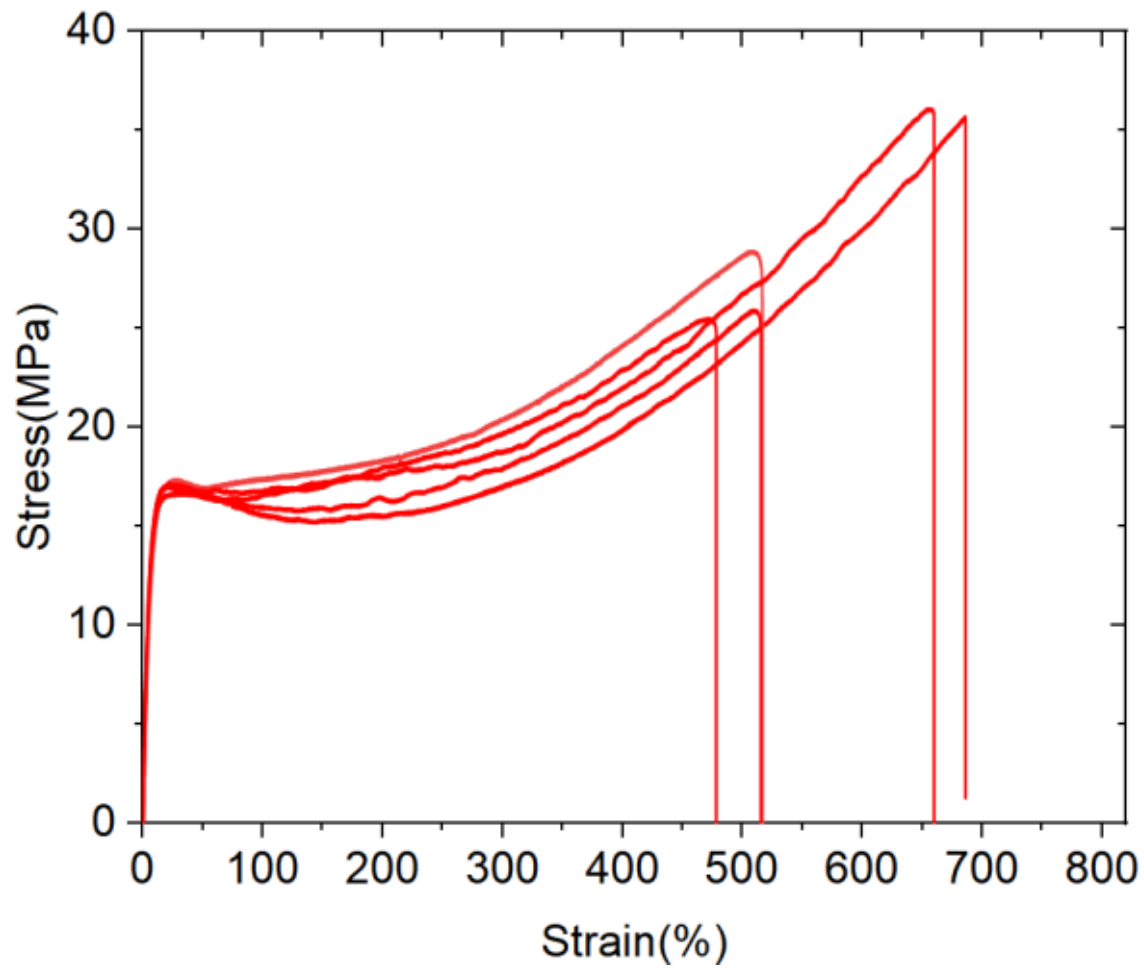

Figure S48. Stress-strain curves of ***d*PE-0.4%-RP-2**.

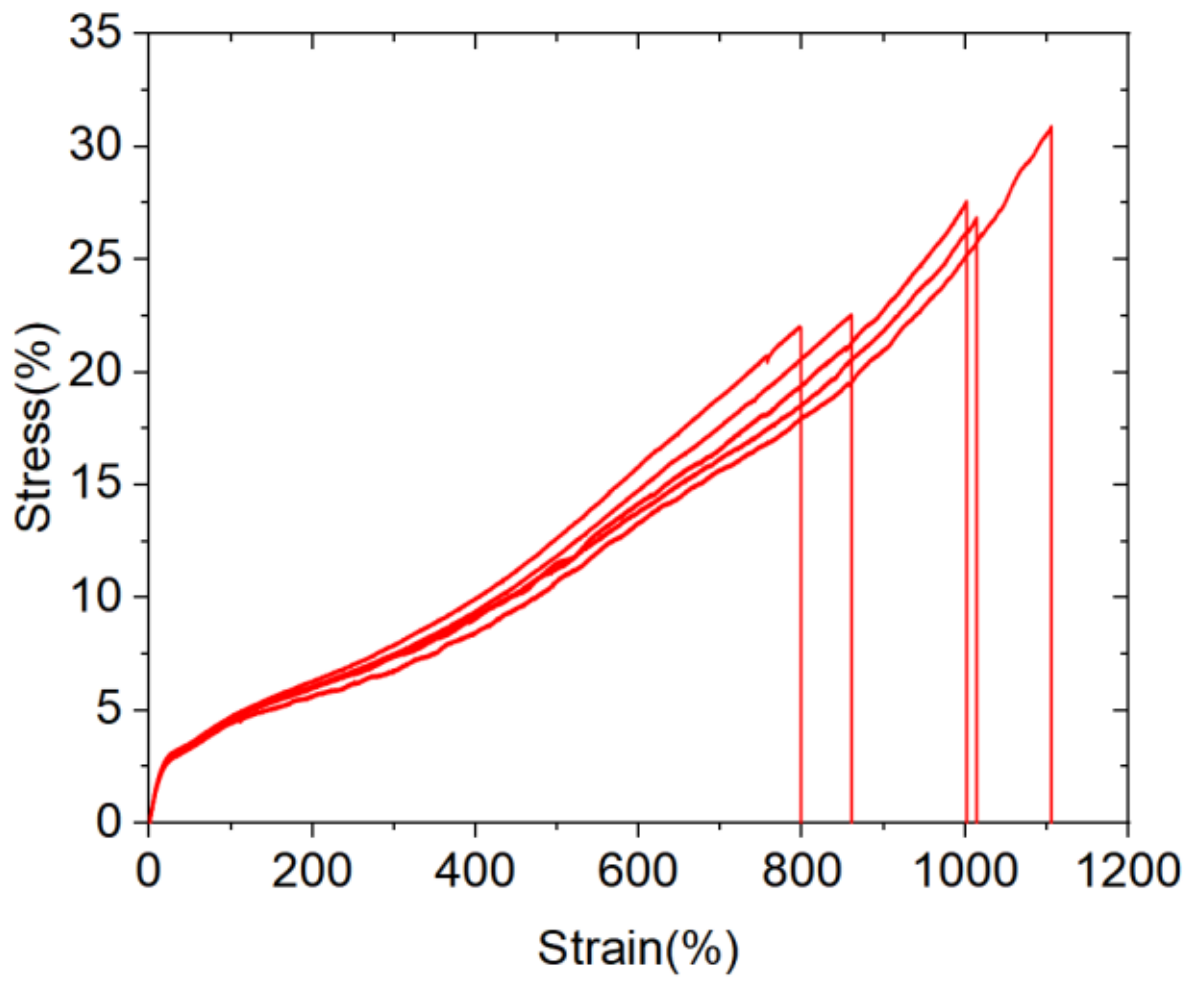

Figure S49. Stress-strain curves of ***d*POE-1.6%**.

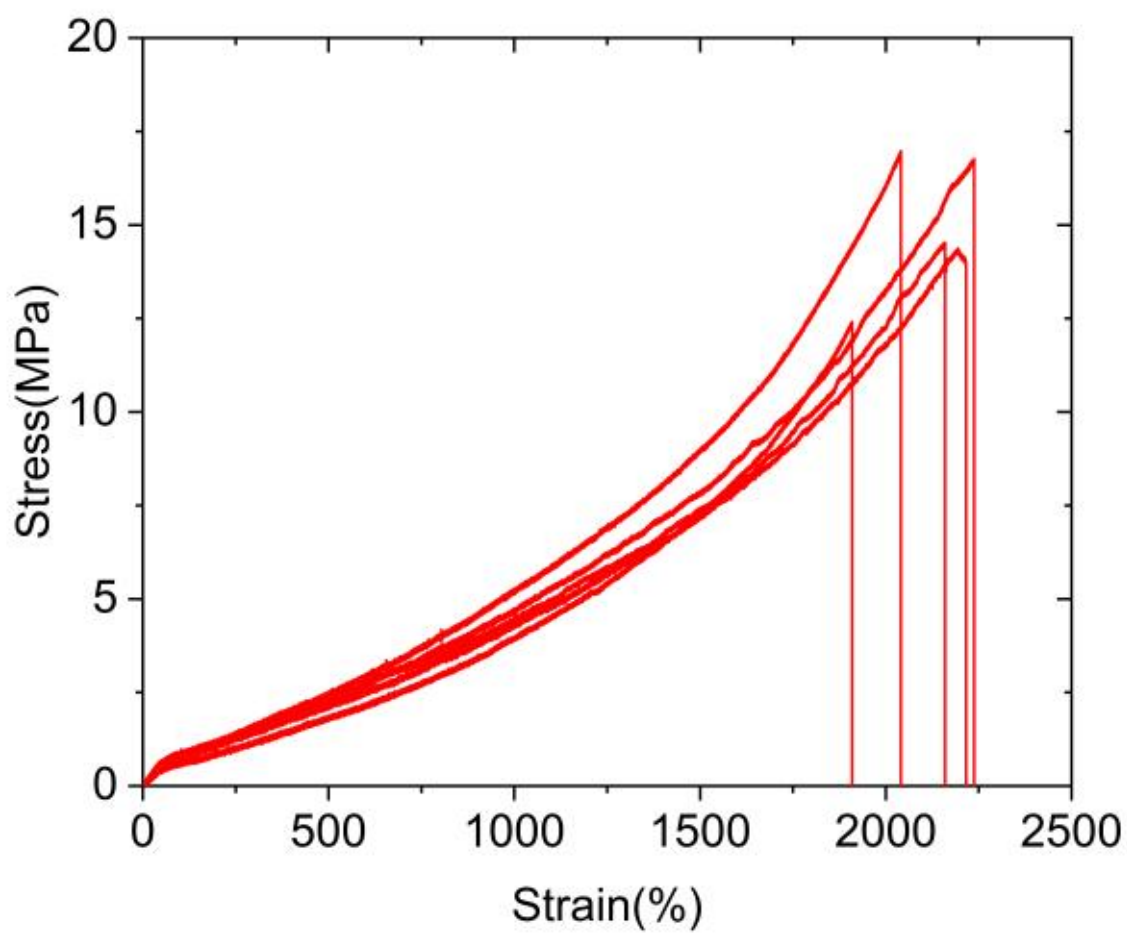

Figure S50. Stress-strain curves of ***d*POE-1.7%**.

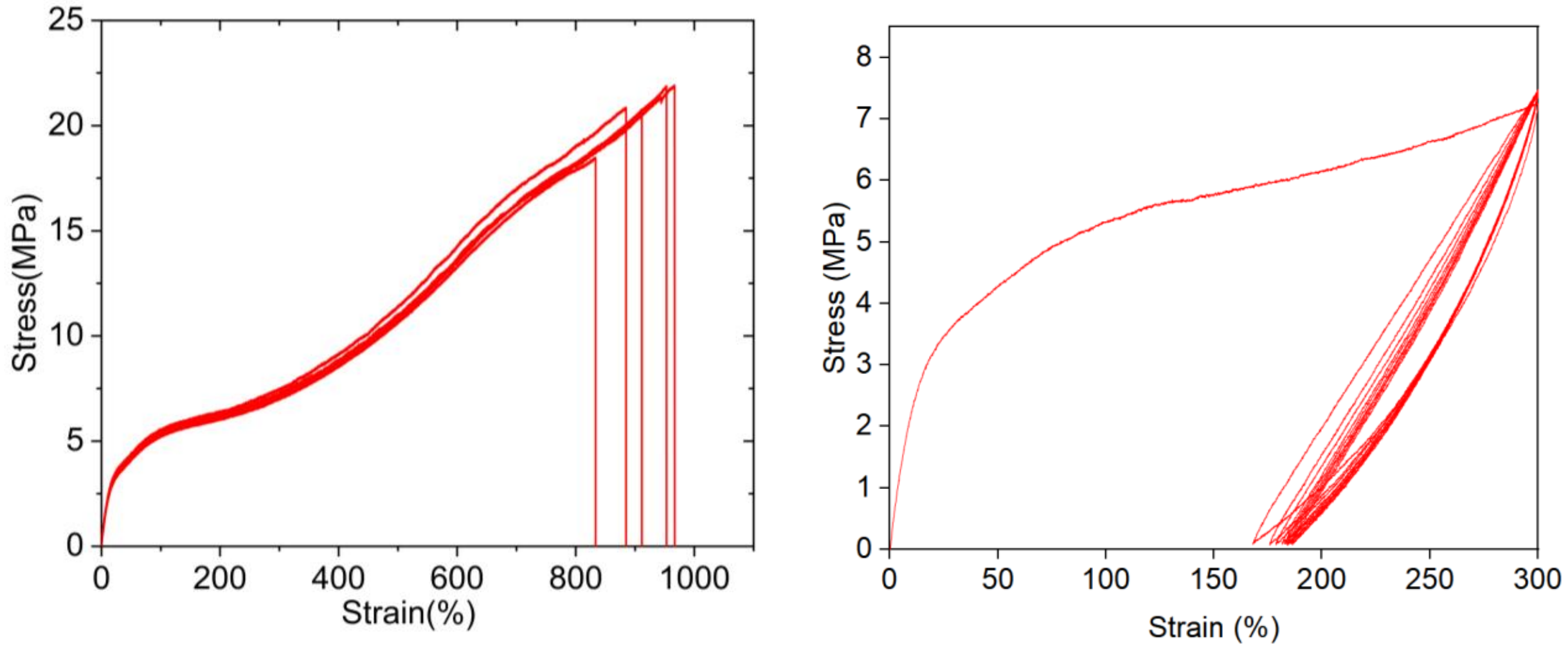

Figure S51. Stress-strain curves of ***d*(*a*-OBC-0.9%)**. Recovery ratio: 38%

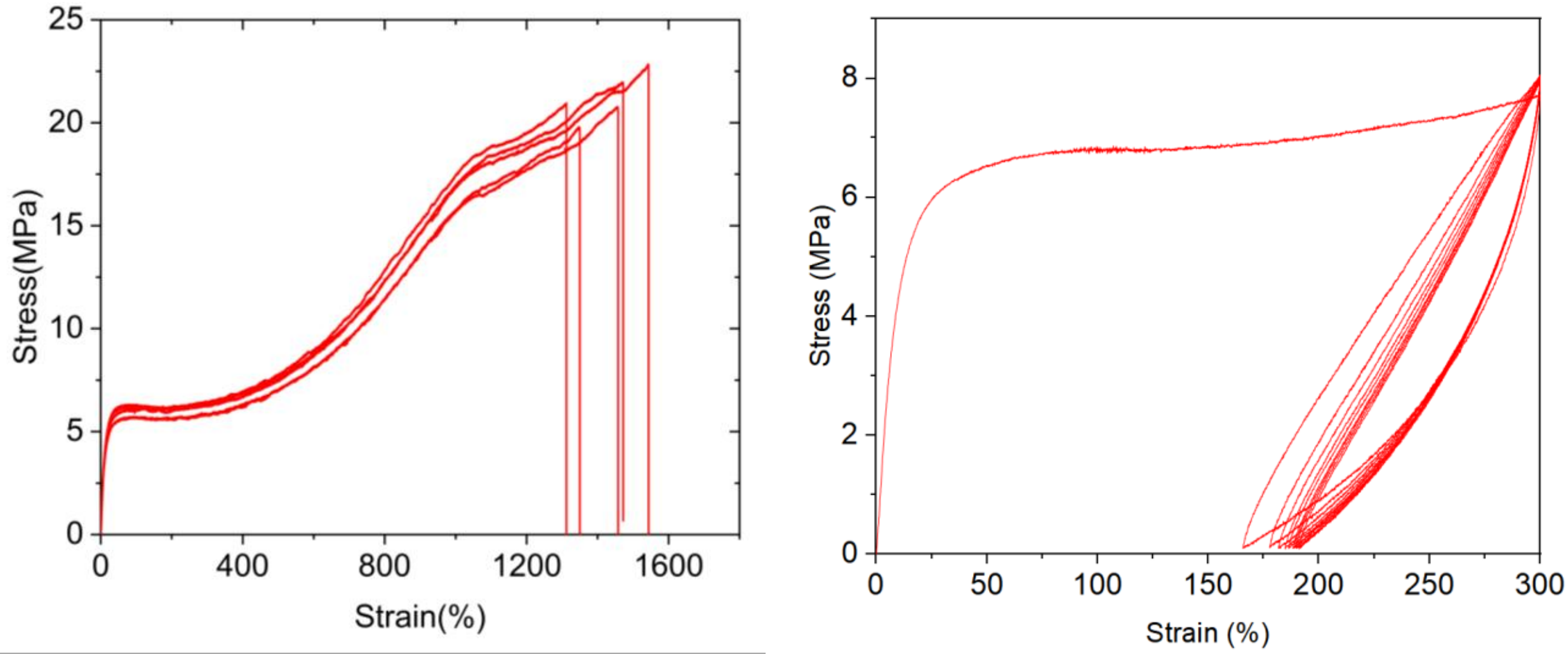

Figure S52. Stress-strain curves of ***d*(*r*-OBC-0.6%)**. Recovery ratio: 36%

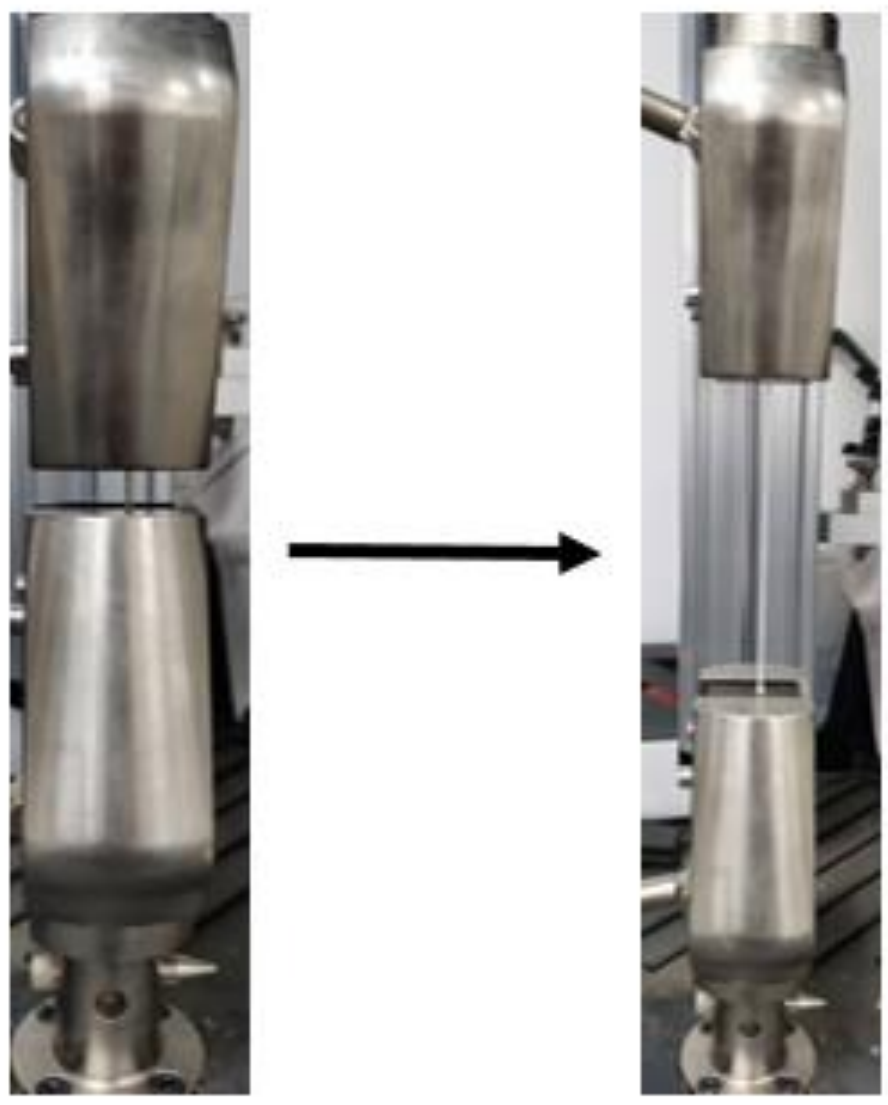

Figure S53. Photos of tensile testing process of ***d*PO.**

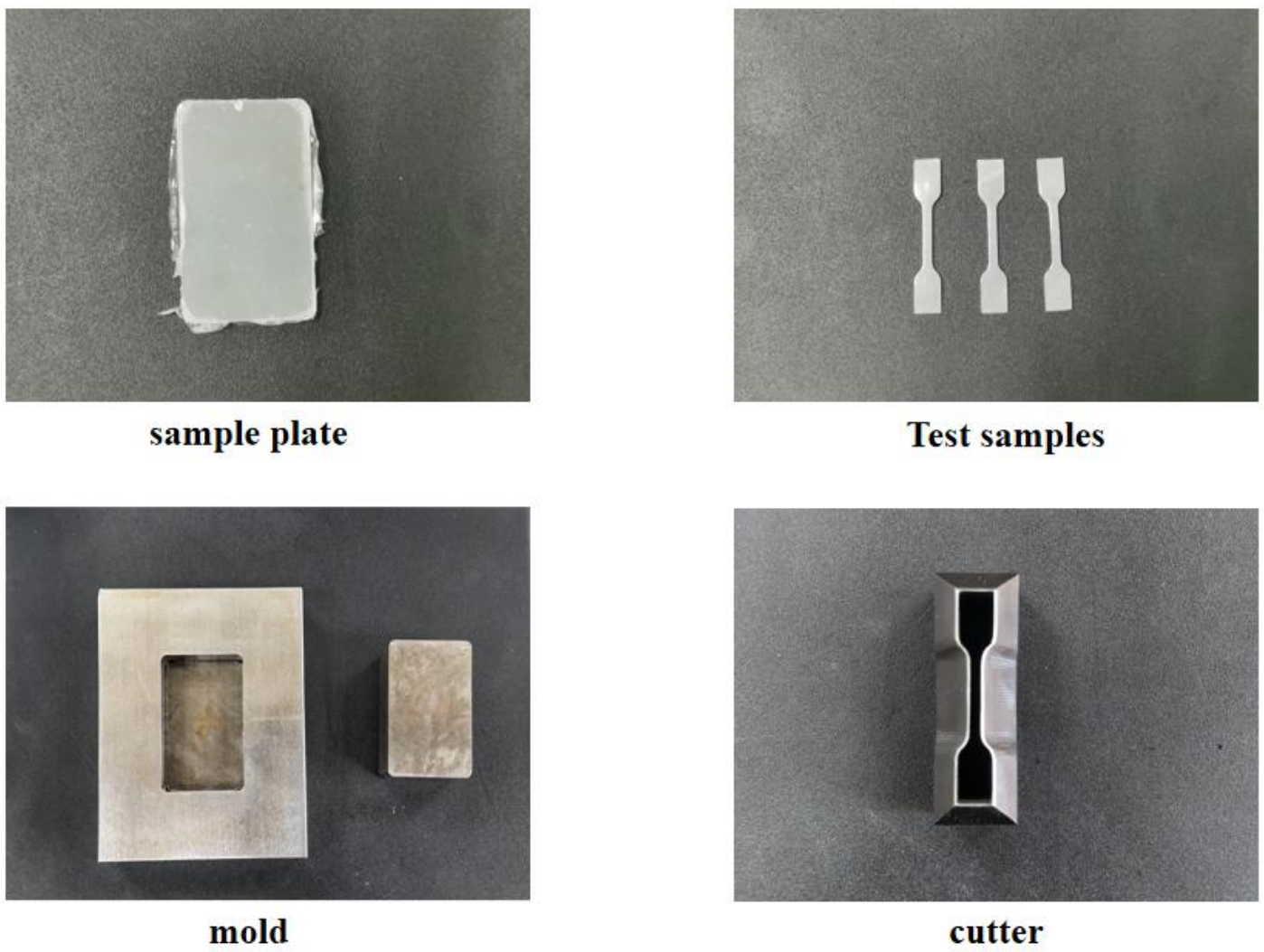

Figure S54. Tensile testing samples of *d*PO and the molds for their production.

4.10 Infrared (IR) spectra of Macromonomer and *d*PO

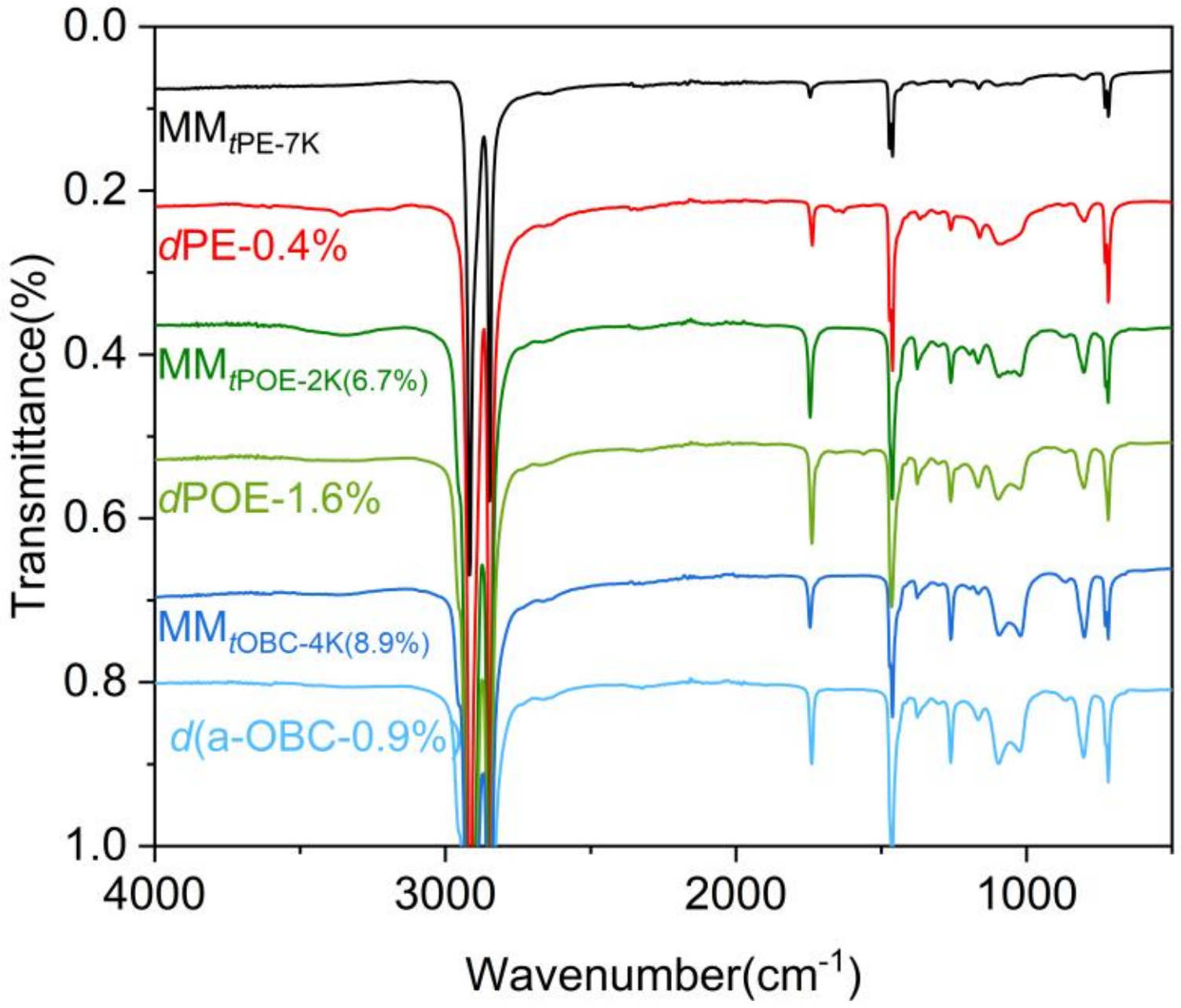

Figure S55. Stacked infrared (IR) spectra of **MM$_{tPE-7K}$**, ***d*PE-0.4%**, **MM$_{tPOE-2K(6.7\%)}$**, ***d*POE-1.6%**, **MM$_{tOBC-4K(8.9\%)}$**, ***d*(*a*-OBC-0.9%)**.

4.11 Photos of water resistance testing

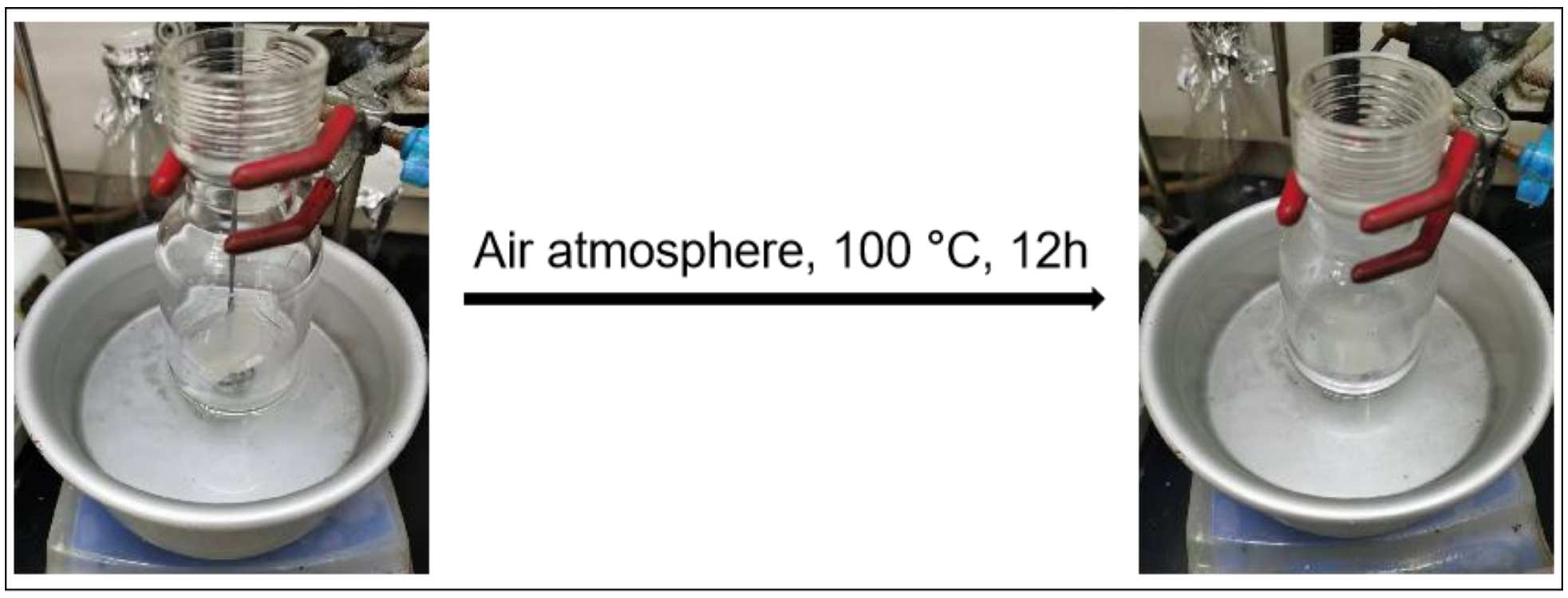

Figure S56. Photos of ***d*PE-0.4%** heated in water at 100 °C for 12 h in an air atmosphere.

The following section 4.12 presents an extended study of the polyolefin materials with in-chain ester group described in this work, serving as a supplement to the material properties discussed in the main text:

4.12 Chemical recycling of polymer with in-chain ester groups.

The presence of in-chain ester groups in the polyolefin backbone confers excellent chemical recyclability to polyolefin materials (Figure S57).

For example, ***d*PE-0.4%** underwent complete depolymerization in MeOH/KOH via ester bond cleavage, yielding the degraded product **MM$_{tPE-7K}$-1**. Its structure was nearly identical to the original macromonomer **MM$_{tPE-7K}$**, with the partial hydrolysis of terminal α-COOMe to α-COOH (Figures S57a-**i**, S31). Notably, the inherent selectivity of ester bond solvolysis is advantageous for recycling mixed plastics. For instance, when blended with conventional HDPE in a mixture of 33 vol% methanol and 66 vol% toluene, only the polyethylene with in-chain ester group degraded, allowing clean separation of **MM$_{tPE-3K}$-1** from HDPE (Figure S57a-**ii**).

Crucially, the degradation products retained the stoichiometric end group functionality, permitting repolymerization. Using Ti(O^{n}Bu)$_4$, α-ester-ω-hydroxy terminated **MM$_{tPE-7K}$-1** was repolymerized to produce ***d*PE-0.4%-RP-1**. The repolymerized polymer was structurally identical to the original polymer (***d*PE-0.4%**), as evidenced by nearly identical ^{1}H NMR and GPC profiles (Figure S57, a-**i** and b). Their thermal and mechanical properties were also comparable: only slight difference was observed in tensile strength (40.0±5.5 MPa to 36.6±5.2 MPa), melting point (134 to 130 °C), and elongation at break (820% to 900%) (Figure S57, c and d). This entire recycling process is quantitative without the addition/removal of extra monomer, confirming the advantage of AB-type telechelic polyolefin-like polymers. The same results were observed in second recycling (Figure S58).

The chemical recycling was extended to more complicated multi-component copolymers, where recycling is typically challenging due to product separation. ***d*(*r*-OBC),** derived from **MM$_{tPE}$** and **MM$_{tPOE}$**, underwent similar depolymerization to yield two well-defined telechelic macromonomers, namely **MM$_{tPE-10K}$-1** and **MM$_{tPOE-4K(24.1\%)}$-1**. Their separation was straightforward: **MM$_{tPOE}$** exists as an oily product at room temperature, while **MM$_{tPE}$** is solid (Figure S59). GPC analysis confirmed the recovered macromonomers matched the original macromonomers (Figure S59a, red and blue lines). The repolymerization afforded ***d*(*r*-OBC-0.6%)-RP-1**, a block copolymer nearly identical to the original ***d*(*r*-OBC-0.6%)** with closely matched melting points (120 vs. 121 °C) and GPC traces (Figure S59, a and b).

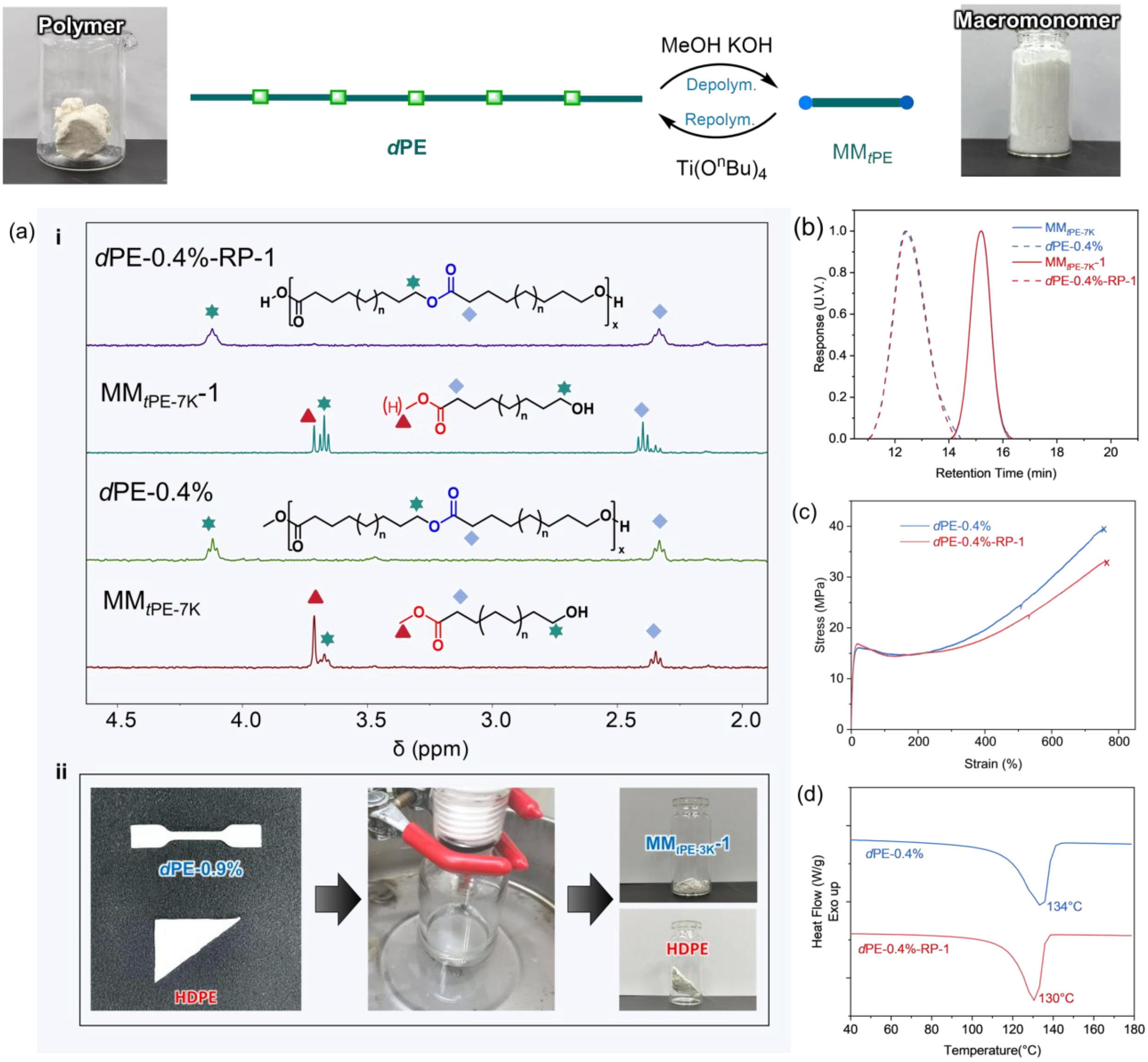

Figure S57. Chemical recycling and properties of polyethylene-like polymers with in-chain esters. (a) i. Stacked ^{1}H NMR spectra of "macromonomer-polymer-macromonomer" circular process (original NMR spectra see Figure S32, S31, S27 and S17); ii. Photos of mixed plastic recycling experiment. A mixture of HDPE and ***d*PE-0.9%** as an example. (b) Stacked gel permeation chromatography (GPC) traces of the original and recycled *d*PE and macromonomer (MM). (c) Stress–strain curves of original (40±5.5 MPa) and recycled *d*PE (36.6±5.2 MPa). (d) Representative differential scanning calorimetry (DSC) traces of original and recycled *d*PE.

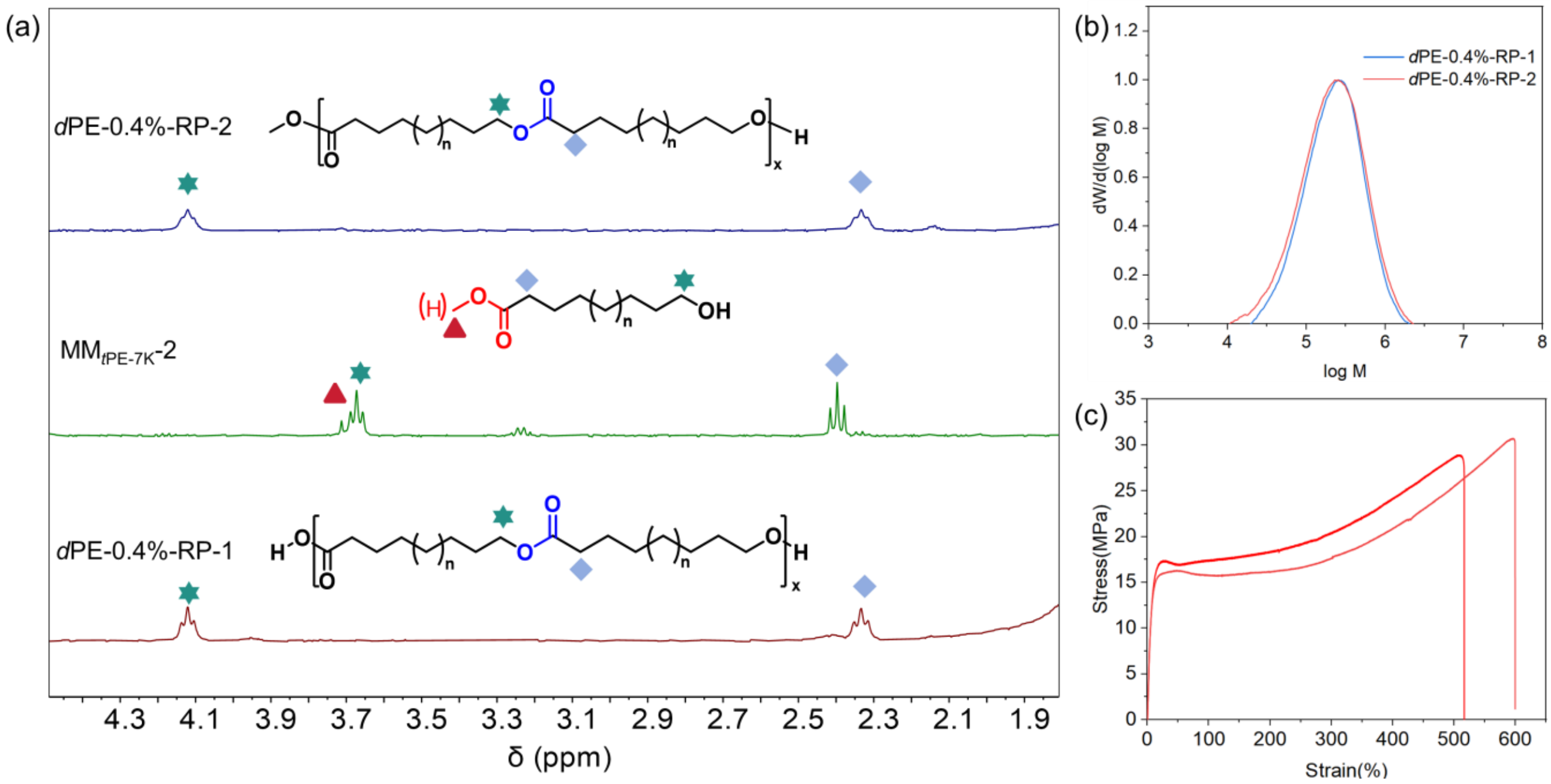

Figure S58. Second recycling of *d*PE. (a) Stacked ^{1}H NMR spectra. (b) Stacked gel permeation chromatography (GPC) traces of the original (***d*PE-0.4%-RP-1**, M_n = 156 kDa, PDI = 1.91) and secondary recycled *d*PE (***d*PE-0.4%-RP-2**, M_n = 135.5 kDa, PDI = 2.23). (c) Stress–strain curves of ***d*PE-0.4%-RP-2** (Stress at break = 29.4±1.6 MPa).

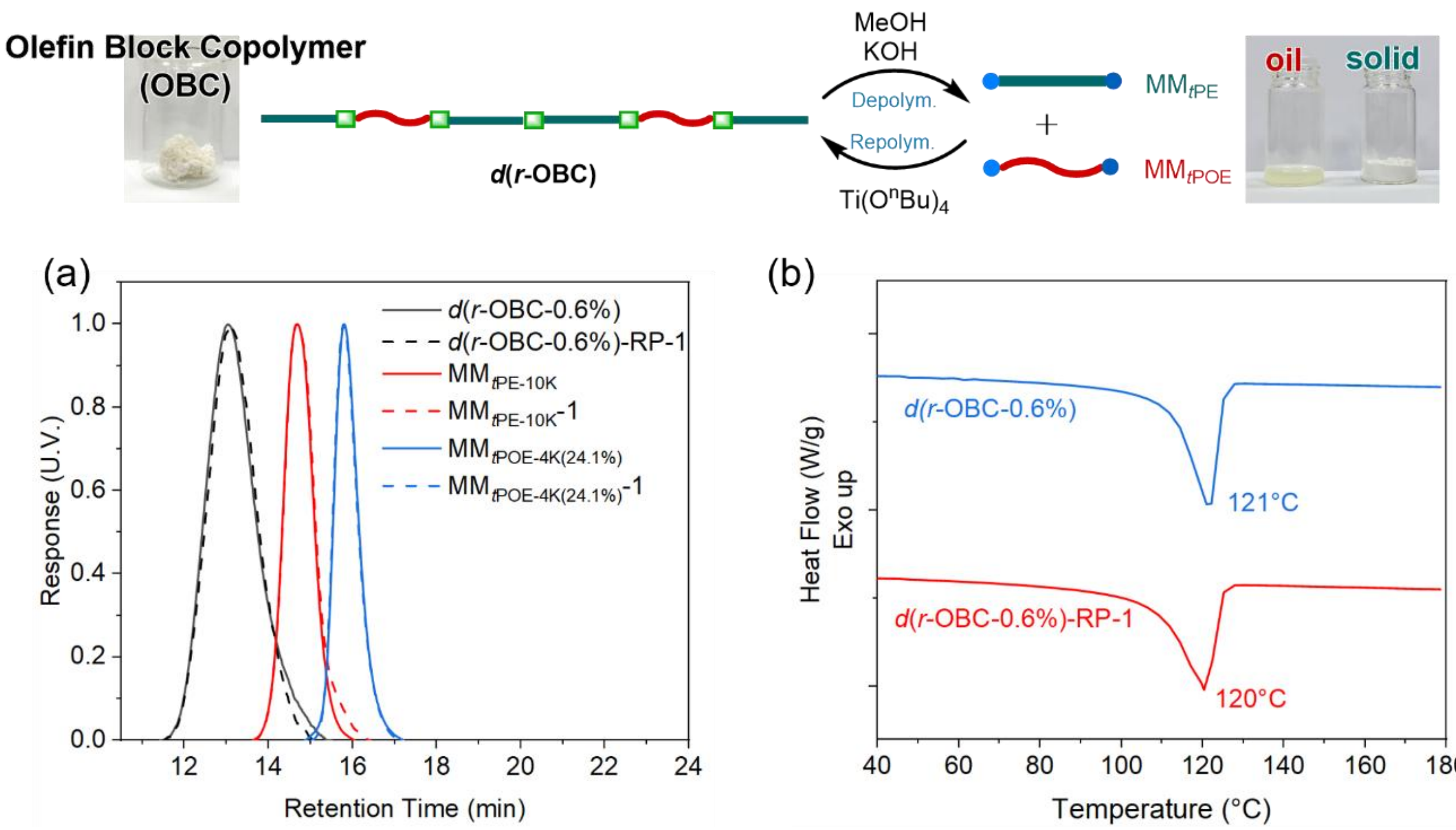

Figure S59. Closed-loop recycling of OBC with in-chain esters [*d*(*r*-OBC-0.6%)]. Photos of original copolymer [***d*(*r*-OBC-0.6%)**] and degradation products of macromonomers (solid: **MM$_{tPE-10K}$-1**; oil: **MM$_{tPOE-4K(24.1\%)}$-1**). (a) GPC traces of original and recycled OBCs and macromonomers. (b) DSC traces of original and recycled OBCs.

5. Supplementary References

[1] Busch, B. B.; Staiger, C. L.; Stoddard, J. M.; Shea, K. J. Living Polymerization of Sulfur Ylides. Synthesis of Terminally Functionalized and Telechelic Polymethylene. *Macromolecules* **2002**, *35*, 8330-8337.

[2] Busch, B. B.; Paz, M. M.; Shea, K. J.; Staiger, C. L.; Stoddard, J. M.; Walker, J. R.; Zhou, X.; Zhu, H. The Boron-Catalyzed Polymerization of Dimethylsulfoxonium Methylide. A Living Polymethylene Synthesis. *J. Am. Chem. Soc.* **2002**, *124*, 3636-3646.

[3] N. Patil, N. Albarbari, N. Hadjichristidis, Perfectly Linear α,ω-Hydroxy-Terminated Polyethylene with Near-Ideal Crystallinity. Angew. Chem. Int. Ed. **2026**, *65*, e22171.

[4] Sulc, R.; Zhou, X.; Shea, K. J. Repetitive sp^3-sp^3 Carbon-Carbon Bond-Forming Copolymerizations of Primary and Tertiary Ylides. Synthesis of Substituted Carbon Backbone Polymers: Poly(cyclopropylidine-*co*-methylidine). *Macromolecules* **2006**, *39*, 4948-4952.
